# Multi-omics analysis unveils immunosuppressive microenvironment in the occurrence and development of multiple pulmonary lung cancers

**DOI:** 10.1038/s41698-024-00651-5

**Published:** 2024-07-23

**Authors:** Jiatao Zhang, Wenhao Zhou, Na Li, Huaming Li, Haitao Luo, Benyuan Jiang

**Affiliations:** 1 Guangdong Lung Cancer Institute, Guangdong Provincial People’s Hospital (Guangdong Academy of Medical Sciences), Southern Medical University, Guangzhou, Guangdong China; 2 Shenzhen Engineering Center for Translational Medicine of Precision Cancer Immunodiagnosis and Therapy, YuceBio Technology Co., Ltd, Shenzhen, China; 3 Department of Thoracic surgery, The Eighth Affiliated Hospital Sun Yat-sen University, Shenzhen, China

**Keywords:** Cancer microenvironment, Lung cancer, Cancer genomics, Tumour heterogeneity

## Abstract

Multiple pulmonary lung cancers (MPLCs) are frequently encountered on computed tomography (CT) scanning of chest, yet their intrinsic characteristics associated with genomic features and radiological or pathological textures that may lead to distinct clinical outcomes remain largely unexplored. A total of 27 pulmonary nodules covering different radiological or pathological textures as well as matched adjacent normal tissues and blood samples were collected from patients diagnosed with MPLCs. Whole-exome sequencing (WES) and whole-transcriptome sequencing were performed. The molecular and immune features of MPLCs associated with distinct radiological or pathological textures were comprehensively investigated. Genomics analysis unveiled the distinct branches of pulmonary nodules originating independently within the same individual. *EGFR* and *KRAS* mutations were found to be prevalent in MPLCs, exhibiting mutual exclusivity. The group with *KRAS* mutations exhibited stronger immune signatures compared to the group with *EGFR* mutations. Additionally, MPLCs exhibited a pronounced immunosuppressive microenvironment, which was particularly distinct when compared with normal tissues. The expression of the *FDSCP* gene was specifically observed in MPLCs. When categorizing MPLCs based on radiological or pathological characteristics, a progressive increase in mutation accumulation was observed, accompanied by heightened chromatin-level instability as ground-glass opacity component declined or invasive progression occurred. A close association with the immunosuppressive microenvironment was also observed during the progression of pulmonary nodules. Notably, the upregulation of B cell and regulatory T cell marker genes occurred progressively. Immune cell abundance analysis further demonstrated a marked increase in exhausted cells and regulatory T cells during the progression of pulmonary nodules. These results were further validated by independent datasets including nCounter RNA profiling, single-cell RNA sequencing, and spatial transcriptomic datasets. Our study provided a comprehensive representation of the diverse landscape of MPLCs originating within the same individual and emphasized the significant influence of the immunosuppressive microenvironment in the occurrence and development of pulmonary nodules. These findings hold great potential for enhancing the clinical diagnosis and treatment strategies for MPLCs.

## Introduction

Multiple pulmonary lung cancers (MPLCs) are defined in patients harboring two or more primary lung cancers simultaneously or sequentially^1^. MPLCs are often diagnosed during early stages, arising from the normal cells within the lung undergoing clonal transformation at multiple foci throughout the lung, resulting in the manifestation of multiple pulmonary nodules^2^. The detection of MPLCs has been increasingly facilitated by the implementation of low-dose computed tomography (CT)-guided lung cancer screening^3^. While surgical intervention represents a common therapeutic approach^4^, it may not be suitable for patients with limited pulmonary function or those with an abundance of pulmonary nodules^5,6^. For these patients, it is critically important to investigate novel systemic therapies, targeted therapy, and immunotherapy. The advancements in next-generation sequencing (NGS) have significantly contributed to the progress in diagnosing and treating MPLCs^1,7^. Previous studies have primarily focused on single-omics profiling, and the integration of molecular changes across multi-omics in the same samples remains largely unexplored, particularly at the transcriptomic levels. Therefore, a comprehensive molecular characterization of MPLCs is crucial for precise diagnosis and optimization of treatment strategy.

Based on radiological characteristics, pulmonary nodules are classified as pure ground-glass opacity (pGGO; non-solid), mixed GGO (mGGO; partly solid), and solid nodules (SNs, devoid of GGO component)^8^. Studies have shown that patients with GGO-associated nodules exhibit better survival outcomes, with nearly 100% 5-year overall survival following resection, while those with SNs are indicative of poorer prognosis^9^. Approximately 20% of pGGO and 40% of mGGO nodules will deteriorate into SNs^10^. Zhang et al. revealed that radiologically distinct GGO-associated lung nodules displayed varying responses to neoadjuvant immunotherapy^11^. The management of GGO-associated lung nodules exhibits significant heterogeneity, with clinicians often relying on their individual experience due to inconsistencies with available guidelines^5^. From the pathological perspective, the development of early-stage lung adenocarcinoma is believed to progress from adenocarcinoma in situ (AIS) to minimally invasive adenocarcinoma (MIA), and ultimately to fully invasive adenocarcinoma (IA)^12^. Surgical resection has been reported to yield a nearly 100% 5-year survival rate for AIS and MIA, which significantly drops for IA^13^. Non-indolent tumors (MIA and IA) harbor higher invasion potential and an increased risk of progressing into lung cancer^14^. Although recent studies have revealed the evolutionary and molecular features of early-stage lung cancers^15–17^, the progression, molecular features, and immune characteristics of radiologically and pathologically distinct lung cancers, particularly in MPLCs, remain largely unexplored.

In our study, we carried out a prospective investigation to examine the heterogeneity among pulmonary nodules and explore the molecular and immune characteristics differences between *EGFR* and *KRAS* mutated tumors. Additionally, our objective was to uncover the evolutionary patterns of molecular and immune contexture in pulmonary nodules that exhibit distinct radiological and pathological features.

## Results

### Mutational landscapes of pulmonary nodules revealed a high prevalence of interfocal heterogeneity

**Fig. 1:**
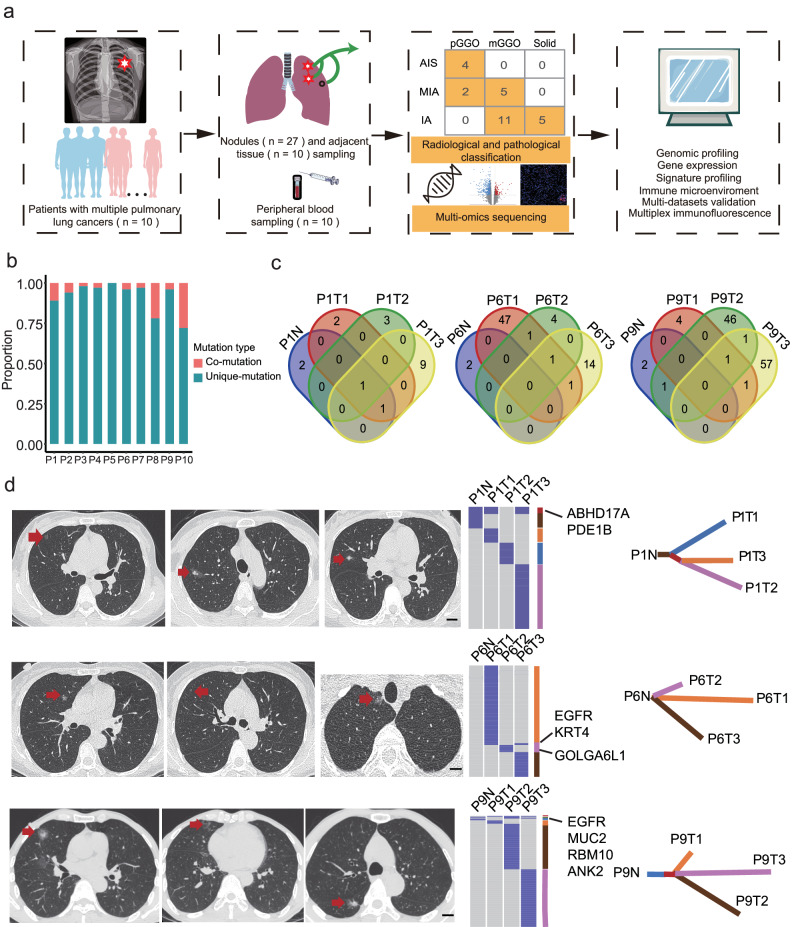
Overview of the study and characteristics of pulmonary nodules. **a** Flowchart of patients and samples included and overview of the methods. Tissue and matched blood samples were collected from patients clinically diagnosed with MPLCs for multi-omics analysis. The distribution of the nodules based on the radiological and pathological classification is shown. **b** Barplots represent the gene mutation types among different pulmonary nodules within each patient. **c** The number of overlapping non-synonymous mutation genes across samples in patients. **d** The representative CT cans, mutational analysis, and construction of phylogenetic trees. The CT scans showing multiple pulmonary cancers (arrows) for each patient. Heatmaps in the middle panel showed the presence (blue) or absence (gray) of non-synonymous somatic mutations. The overlapping mutation genes were indicated on the right side. Phylogenetic trees represented the clonal structure of samples. Scale bar, 1 cm. MPLCs multiple pulmonary lung cancers, CT computed tomography.

A total of 47 samples for sequencing were obtained from ten patients diagnosed with MPLCs who underwent surgical resections between September 2018 and September 2019 (Fig. 1a) (for clinicopathological characteristics, see Table 1). Based on GGO component evaluated by preoperative CT scans, the pulmonary nodules were divided into pGGO (*n* = 6), mGGO (*n* = 16), and solid (*n* = 5). Next, histological staining was used to estimate the invasive degree, which reclassified the 27 primary lung cancer samples into AIS (*n* = 4), MIA (*n* = 7), and IA (*n* = 16) (Supplementary Table 1). All samples were subjected to whole exome sequencing (WES) and whole transcriptome sequencing.

**Table 1:** Clinicopathological features of 10 patients with MPLCs

Characteristics	Number (frequency)
*Median Age at diagnosis (range), years*	64.5 (55 – 78)
*Histologic type*	
LUAD	10 (100%)
*Smoking*	
YES*	2 (20%)
NO	8 (80%)
*Nodule location*	
Same lobe	7 (70.0%)
Ipsilateral	3 (30.0%)
*Radiological type* (*n* = 27)	
pGGO	6 (22.22%)
mGGO	16 (59.26%)
Solid	5 (18.52%)
*Pathological type* (*n* = 27)	
AIS	4 (14.81%)
MIA	7 (25.92%)
IA	16 (59.25%)
*Nodules presentation time* (*n* = 27)	
Synchronous	27 (100%)
Metachronous	0 (0%)
Nodules size (mm) (*n* = 27)	
≤ 5	0 (0%)
5–8	6 (22.22%)
> 8	21 (77.78%)

Data are presented as number or *n* (%). *MPLCs* multiple pulmonary lung cancers, *LUAD* lung adenocarcinoma, *pGGO* pure ground-glass opacity, *mGGO* mixture ground-glass opacity, *AIS* adenocarcinoma in situ, *MIA* minimally invasive adenocarcinoma, *IA* invasive adenocarcinoma. “*” indicates that the two patients (P04 and P09) have the smoking history.

By analyzing WES data, we first compared the mutational landscapes of pulmonary nodules from the same individual (Supplementary Fig. 1). An average of 41 somatic alterations were identified per nodule (Supplementary Table 2). For each patient, only 2–29% (mean 7.8%) mutational sites were shared between any nodule pair, revealing a high prevalence of interfocal heterogeneity in MPLCs (Fig. 1b). The number of overlapping non-synonymous mutations genes across samples were shown in Fig. 1c and Supplementary Fig. 2. Notably, three co-mutated genes (*ABHD17A*, *KRT4*, and *EGFR*) were observed within three nodules in four patients, namely P1, P6, P8, and P9 (Supplementary Fig. 3). To further explore the detailed relationship of individual nodule from the same patients, phylogenetic trees were constructed based on mutational profiles (Fig. 1d and Supplementary Fig. 4). Nodular lesions derived from the same lobe of P1’s lung displayed minimal overlap in mutated genes, except for the aforementioned shared mutations. Similar phenomena were observed among the nodular lesions of other patients as well. This implies independent origins and profound heterogeneity among the pulmonary nodules, consistent with recent studies demonstrating the independent clonal origins of MPLCs^18,19^.

### *EGFR* and *KRAS* mutated tumors showed distinct immunogenomic features

**Fig. 2:**
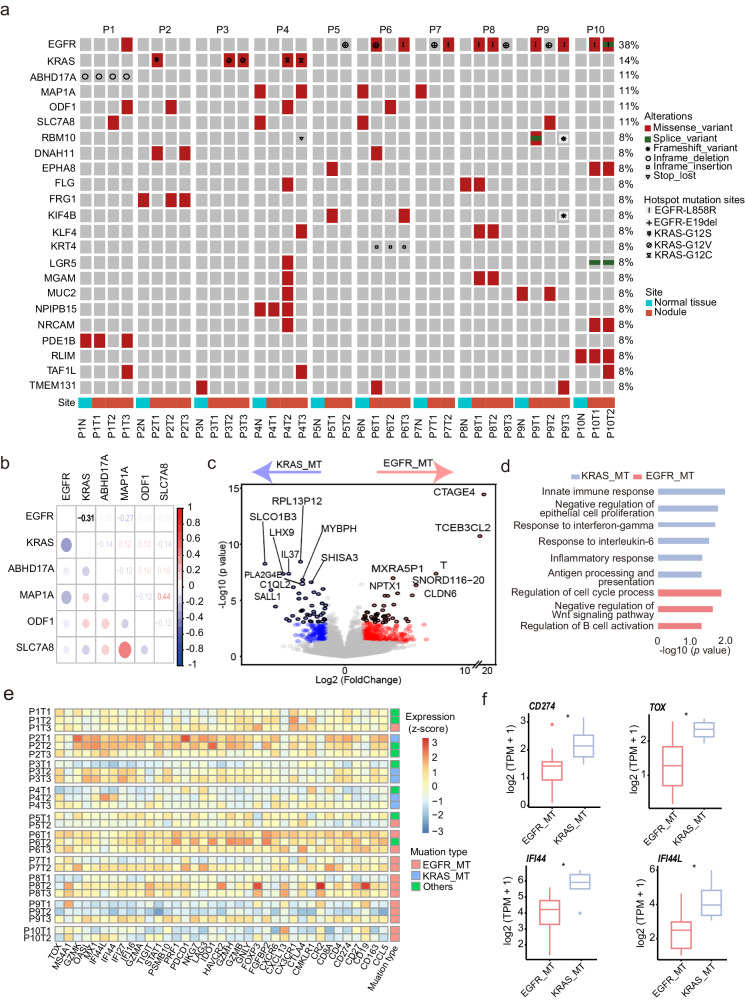
Genomic and transcriptomic profiling of *EGFR*_MT and *KRAS*_MT tumors. **a** Mutational landscape of 37 samples in 10 patients with MPLCs. **b** Correlation between genes with mutated frequencies more than 10%, assessed by Spearman’s rank correlation coefficient (Rho). **c** Volcano plot represents the comparison of DEGs between *KRAS*_MT and *EGFR*_MT groups. **d** The enriched functional pathways in *KRAS*_MT and *EGFR*_MT groups. **e** The heatmap displays the expression of immune-related genes across 27 samples. Expression levels are normalized (z-scores) to show relative differences among samples. **f** Comparison of expression of immune-related genes between *KRAS*_MT and *EGFR*_MT groups. *KRAS*_MT, *KRAS* mutation; *EGFR*_MT, *EGFR* mutation; DEGs, differentially expressed genes. *p < 0.05.

By detailed analysis of mutation profiling of MPLCs, we found that *EGFR* and *KRAS* were the most frequently mutated genes (Fig. 2a and Supplementary Table 2). A Spearman’s correlation coefficient analysis showed that *EGFR* and *KRAS* mutations (*EGFR*_MT and *KRAS*_MT) were mutually exclusive (Fig. 2b). Earlier clinical studies have highlighted that tumors with *EGFR*_MT are more resistant to immunotherapy than those with *KRAS*_MT^20–22^, which promoted us to investigate the underlying mechanisms.

**Fig. 3:**
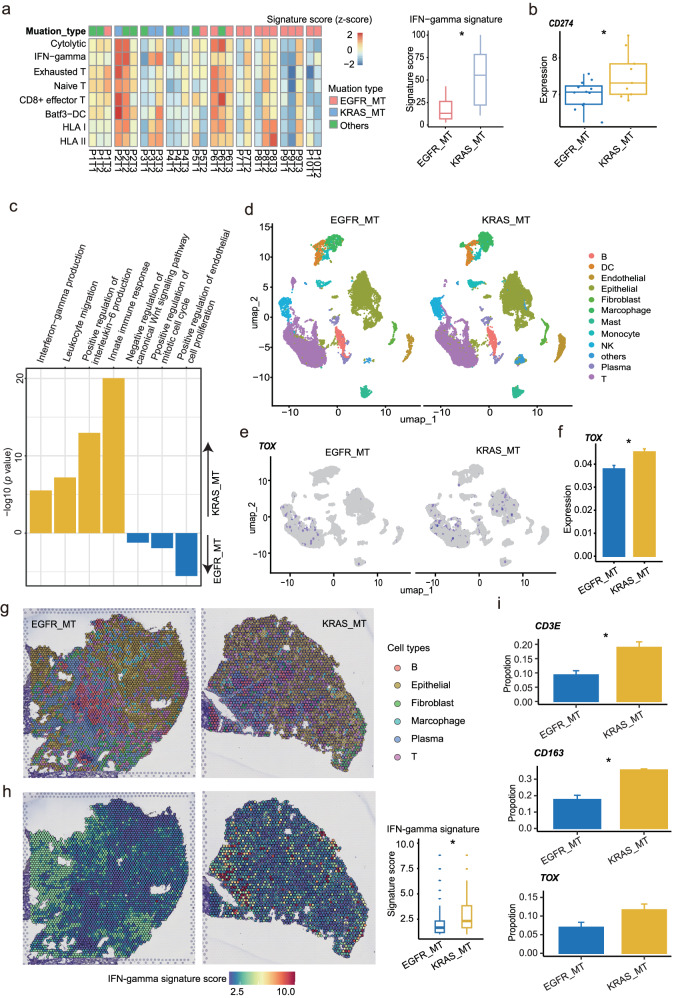
Analysis of immune characteristics of *KRAS*_MT and *EGFR*_MT groups based on different data types. **a** Estimation of immune-related signatures across 27 samples. Left panel showed the heatmap of scores related to immune-related signatures. Signature scores were normalized (z-scores) to show relative differences among samples. Right panel showed the comparison of IFN-gamma signature score between *KRAS*_MT and *EGFR*_MT groups. **b** Validation of the high expression of *CD274* in *KRAS*_MT group using an RNA profiling dataset generated by the NanoString nCounter Platform. **c** The enriched functional pathways in *KRAS*_MT and *EGFR*_MT groups using nCounter dataset. **d** UMAP plots of 26,710 single cells colored according to the major cell types in scRNA-seq dataset (HRA003826). **e** UMAP plots showing the expression of *TOX* gene between *EGFR*_MT and *KRAS*_MT groups. **f** Comparison of *TOX* gene expression between *EGFR*_MT and *KRAS*_MT groups. **g** Spatial plots colored according to the 6 major immune cell types in two representative spatial transcriptome sections. **h** Comparison of IFN-gamma signature score between *EGFR*_MT and *KRAS*_MT groups at spatial level. **i** The validation of the representative cell markers’ expression proportions within the cells between the *EGFR*_MT and *KRAS*_MT groups on spatial data. scRNA-seq, single cell RNA-sequencing; UMAP Uniform manifold approximation and projection. Bar graph represents mean ± standard error of the mean (SEM). *p < 0.05.

Analysis of genomic features such as tumor mutational burden (TMB), tumor neoantigen burden (TNB), and chromosomal instability failed to unveil meaningful indicators associated with immunotherapy response (Supplementary Fig. 5), which highlighted the potential role of transcriptome programs in leading to different treatment responses in the two types of mutational tumors. We found that the *KRAS*_MT group displayed upregulation of genes mainly participating in immune-related responses, while the *EGFR*_MT group showed enrichment of the cell cycle process (Fig. 2c, d). Next, we performed a deep assessment of immune-related genes and signatures. The results showed that the levels of *CD274* (encoding PD-L1), the exhausted T (Tex) cell marker *TOX*, and interferon-gamma (IFN-gamma) related genes *IFFI44* and *IFI44L*, were significantly upregulated in *KRAS*_MT group compared to *EGFR*_MT group (all *p* < 0.05) (Fig. 2e, f and Supplementary Table 3). In addition, pulmonary nodules harboring *KRAS*_MT exhibited significantly higher IFN-gamma signatures compared to those with *EGFR*_MT (*p* < 0.05) (Fig. 3a and Supplementary Table 4), implying that tumors harboring *EGFR*_MT might exhibit reduced capacity to suppress tumor proliferation and antigen presentation efficiency when compared to tumors with *KRAS*_MT, as indicated by Hastings et al.^23^. To further validate our results, we collected and analyzed an RNA profiling dataset generated by the NanoString nCounter Platform, including 28 early lung carcinogenesis samples with *KRAS* and *EGFR* mutations^24^. In accordance with the above findings, the expression of *CD274* and the genes involved in immune-related responses were significantly higher in *KRAS*_MT group (Fig. 3b), whereas the *EGFR*_MT group demonstrated enrichment in cell cycle processes (Fig. 3c). Moreover, by analyzing the single-cell RNA sequencing (scRNA-seq) data of *KRAS*_MT and *EGFR*_MT lung adenocarcinoma samples generated by our previous study^25^, we found that in *KRAS*_MT samples, *TOX* was expressed not only in T cells, but also in epithelial cells (Fig. 3d, e). Meanwhile, we further confirmed that the *TOX* gene was overexpressed in *KRAS*-MT samples (Fig. 3f). The *TOX* expression was reported to correlate with immune cells infiltration and T cells exhaustion in lung adenocarcinoma^26^. Next, we integrated the scRNA-seq data with the spatial RNA profiling data generated by our previous study^25^ to investigate the differences between the two mutational subtypes at the spatial level and characteristics of spatial distribution of immune cells (Fig. 3g). The results demonstrated an obvious infiltration and extensive distribution of immune cells, such as T cells and macrophages, within the tumor samples. Through quantitatively comparative analysis, we found that the IFN-gamma signature score and several immune cell marker genes including *TOX*, *CD3E*, and *CD168* were enriched in *KRAS*_MT group (Fig. 3h, i). Finally, we performed multiplex immunofluorescence (mIF) staining using re-collected samples, including 3 *KRAS*_MT and 3 *EGFR*_MT. It was observed that samples with *KRAS*_MT had a higher infiltrative abundance of Treg and B cells than *EGFR*_MT (Supplementary Figs. 6-7 and Supplementary Table 5). These results suggested that the prominent immune characteristics of *KRAS*_MT group may be a potential mechanism contributing to the better response to immunotherapy.

### Immunosuppressive microenvironment may facilitate the occurrence of pulmonary nodules

**Fig. 4:**
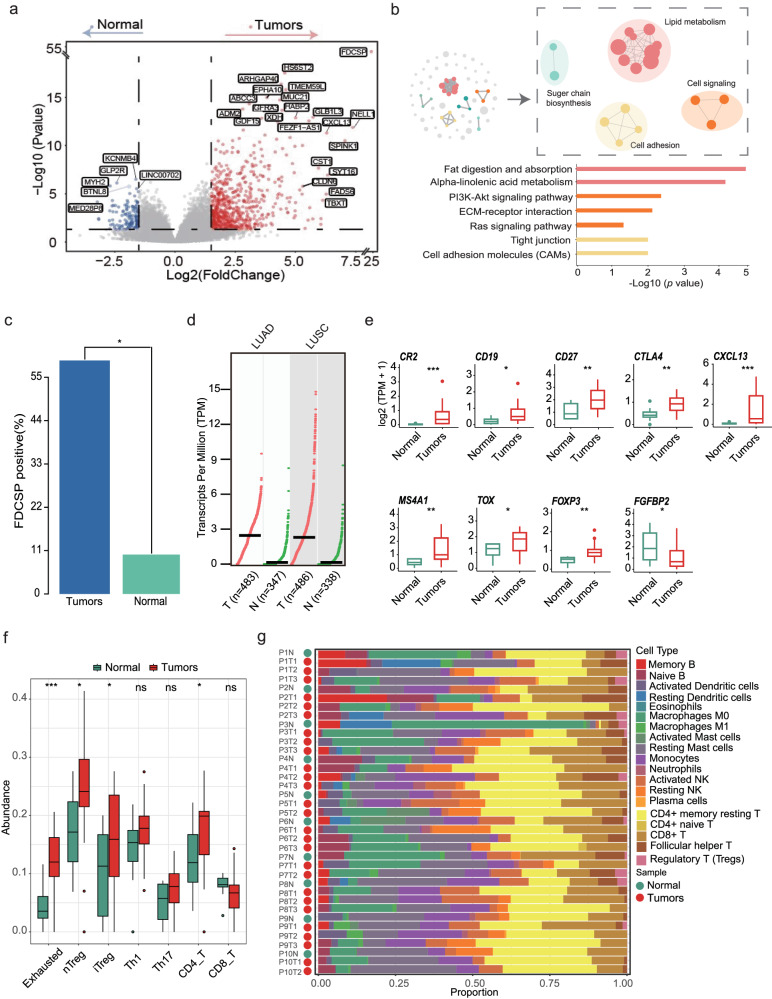
Transcriptomic features of MPLCs compared with normal tissues. **a** Volcano plot represents the comparison of DEGs between pulmonary nodules and normal adjacent tissues. **b** The enriched functional pathways based on the DEGs among pulmonary nodules and normal adjacent tissues. The similar pathway would be clustered in the network. **c** Barplot represented *FDCSP* positive rate (expression > 0) between tumors and normal tissues. **d** The TPM of *FDCSP* in tumor (LUAD and LUSC) and normal groups. Data sourced from TCGA. Black lines represent the median of TPM. **e** Comparison of immune-related genes expression between MPLCs and normal tissues. **f** Comparison of T cell abundance between tumors and normal tissues estimated by xCell. **g** The proportion of 21 types of immune cells was estimated by Cibersort in 10 patients. DEGs, differentially expressed genes; TPM, transcripts per million; nTreg, natural regulatory T cell; iTreg, induced regulatory T cell; Th1, first helper T cell; Th17, 17th helper T cell. *p < 0.05. **p < 0.01; ***p < 0.001.

Next, at transcriptional level, changes in signaling pathways and immune microenvironment that contribute to nodule initiation were investigated. Compared with normal tissues, a total of 828 genes were dysregulated in pulmonary nodules (Fig. 4a and Supplementary Table 6), which were mainly enriched in metabolism-related pathways, ECM-receptor interaction, and PI3K-Akt signaling pathways (Fig. 4b). Notably, we found *FDCSP* gene, which has been found to be expressed in follicular dendritic cell^27^, was specifically expressed in the nodule tissues (*p* = 0.0102). Further analysis based on TCGA database showed that *FDCSP* was also highly expressed in lung adenocarcinoma, indicating its potential role in the occurrence and development of pulmonary nodules (Fig. 4c, d). Moreover, we observed a dramatically increased expression of immune-related genes in MPLCs compared with the normal group (*p* < 0.05) (Fig. 4e). To gain insights into the immune microenvironment changes of nodule occurrence, the relative abundance of immune cells was estimated. Compared with normal tissues, the abundance of CD8 + T cells decreased in MPLCs, while CD4 + T cells increased significantly (Fig. 4f), which was mainly caused by the increase of regulatory T cell (Tregs) (Fig. 4f, Supplementary Fig. 8, and Supplementary Table 7). Meanwhile, we found that the exhausted cells were significantly increased in MPLCs. These results were further confirmed by an independent deconvolution algorithm (Fig. 4g, Supplementary Fig. 9, and Supplementary Table 8). Taken together, these results suggested that the formation of immunosuppressive microenvironment may facilitate the nodule occurrence.

### Genomic and immune features associated with radiologically or pathologically distinct clinical entities of pulmonary nodules

**Fig. 5:**
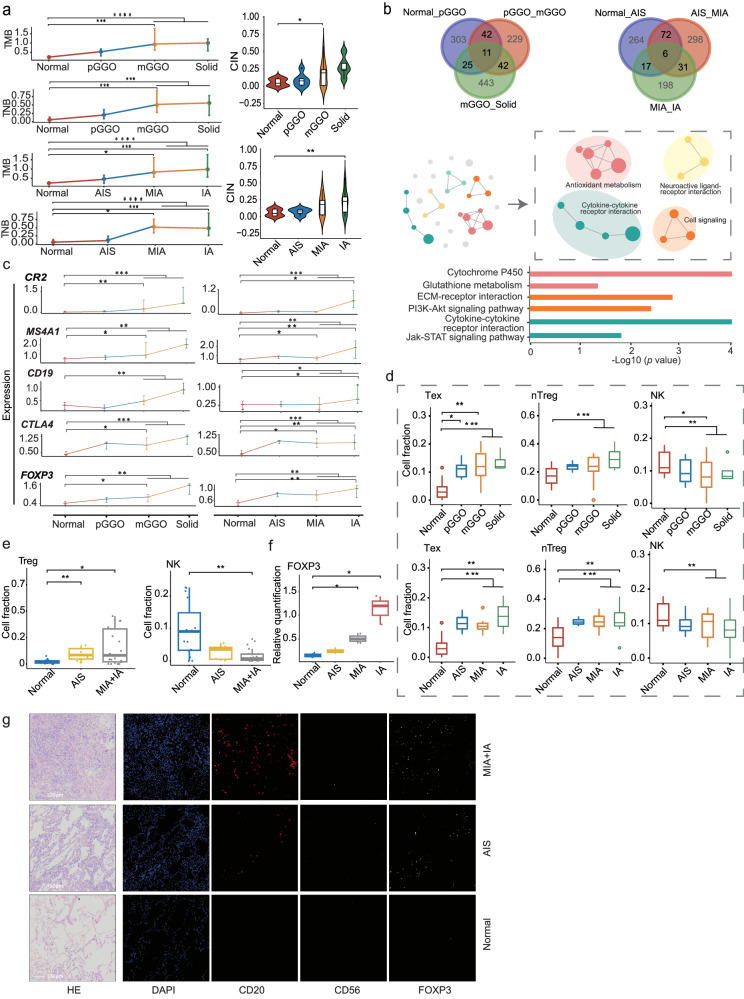
Alterations in genomic and transcriptomic characteristics during the progression of MPLCs. **a** Line graphs represent the change of TMB and TNB across each stage of radiological and pathological classification. Violin plot represents the comparison of chromosomal instability among each stage of radiological and pathological classification. **b** Analysis of DEGs and corresponding enriched functional pathways. The upper Venn panel shows the overlapping DEGs among the distinct stages of MPLCs. The middle panel shows the enriched pathways network and the similar pathways clusters. The bottom panel represents the selected functional pathways. **c** Changes in B cell-related and Treg-related genes in distinct stages of nodules. **d** Comparison of distinct immune cell fraction among each stage based on radiological and pathological classification. **e** The proportion of Treg and NK cells among normal lung tissues and cancer tissues analyzed using public scRNA-seq dataset (HRA001130). **f** Quantification of the relative expression level of *FOXP3* among tissues using IHC data. **g** Representative images from P17 with normal to invasive subregions for HE staining, CD20 B cells (magenta), CD56 NK cells (orange), FOXP3 Treg cells (cyan), and DAPI (blue), as determined by using multiplex immunofluorescence staining. Scale bar, 100μm. pGGO, pure ground-glass opacity; mGGO, mix ground-glass opacity; AIS, adenocarcinoma in situ; MIA, minimally invasive adnocarcinoma; IA, invasive adenocarcinoma; Treg, regulatory T cell; Tex, exhausted T cell; nTreg, natural T cell; IHC, immunohistochemistry. Error bar represents mean ± standard error of the mean (SEM). *p < 0.05. **p < 0.01; ***p < 0.001.

Radiologically or pathologically distinct clinical entities of pulmonary nodules often exhibited different therapeutic effects and clinical outcomes^28,29^. In view of this, we performed a systematic study to delineate the molecular and immune features of different radiological and pathological textures of MPLCs. First, at the genomic level, we found that the accumulation of mutations gradually increases accompanied by increased chromatin-level instability as GGO component decline or invasive progression (Fig. 5a). In addition, 246 dysregulated genes were identified across the distinct stages of MPLCs. Pathway enrichment analysis showed these dysregulated genes were primarily involved in metabolic pathways, cytokine-cytokine receptor interactions, and cell signaling cascades (Fig. 5b). Besides, an upregulation of B cell marker genes (*CR2*, *MS4A1*, and *CD19*) and Treg-related genes (*CTLA4* and *FOXP3*) were observed in the high-risk nodules, particularly in mGGO and solid nodules (all *p* < 0.01) or MIA and IA (all *p* < 0.05) (Fig. 5c). Furthermore, through immune cell abundance analysis we found that the content of immune-suppressed cells such as exhausted cells and Tregs markedly increased in the high-risk stages, while NK cells showed a decreasing trend (Fig. 5d and Supplementary Figs. 10-11). To further substantiate these findings, public scRNA-seq data^30^ of early lung carcinogenesis samples covering distinct pathological textures, such as AIS, MIA, and IA were analyzed. We found a significant increase in Treg cells and decrease in NK cells as nodules progressed (Fig. 5e and Supplementary Fig. 12). Additionally, the expression of *FOXP3* was increased in high-risk stages based on immunohistochemistry data^30^, which further supported the immunosuppressive microenvironment in the high-risk stages (Fig. 5f). Finally, we performed mIF experiments using MPLC samples covering normal, AIS, MIA/IA subregions. The results further verified that the invasive subregion was characterized by a higher proportion of *FOXP3* and *CD20* positive cells along with a lower proportion of *CD56* positive cells (Fig. 5g, Supplementary Figs. 13-14, and Supplementary Table 5). Overall, nodule progression showed a strong association with the immunosuppressive microenvironment, which may explain to a certain extent the differences in clinical outcomes of different radiological or pathological textures of MPLCs.

## Discussion

The extensive utilization of CT-guided screening and availability of high-resolution diagnostic CT scans have led to a substantial increase in the identification of MPLCs^31,32^. The emergence of MPLCs poses a new challenge even in the era of precision medicine. Therefore, we conducted a thorough exploration of the genomic and transcriptomic profiles of MPLCs to facilitate precise diagnosis and therapy.

A mounting body of evidence substantiates the proposition that multiple pulmonary nodules within the lungs originate independently from each other^10,18,19^. Our study unveiled discernible variations in genomic profiles among the pulmonary nodules, further validating prior investigation^14,19,33^. The presence of such heterogeneity among the nodules indicates the ineffectiveness of a singular drug in targeting diverse nodules, as highlighted by Zhang et al.^11^. Consequently, additional exploration is imperative to identify shared genetic attributes across all tumors within an individual, which may advance the application of targeted therapy in MPLC patients. whole transcriptome data revealed the specific expression of *FDCSP* in pulmonary nodules, which encodes a tiny, secreted protein that was discovered in follicular dendritic cells^34^. *FDSCP* was significantly associated with immune-checkpoint inhibitors (ICIs) response and thought to contribute to tumor metastasis by promoting cancer cell migration and invasion^35^. Thus, *FDCSP* is a potential biomarker for diagnosis and treatment of patients with pulmonary nodules.

For the first time, we depicted the genomic landscape of MPLCs. Consistent with previous studies^10,14,36,37^, *EGFR* and *KRAS* mutations are common in pulmonary nodules. Our investigation revealed the exclusive presence of *EGFR*_MT and *KRAS*_MT within tumors. Tumors harboring *EGFR*_MT or *KRAS*_MT displayed a remarkable increase in both TMB and TNB compared to normal tissues. Notably, *EGFR*_MT cancers exhibited a higher level of chromosome instability in comparison to tumors with *KRAS*_MT. These insightful findings provide compelling evidence that tumors carrying *EGFR*_MT are more prone to progressing into aggressive manifestations. *EGFR* mutations are associated with inferior response to ICIs in patients with advanced NSCLCs^38,39^. Similarly, the efficacy of ICIs in MPLC patients may be affected by the status of driver gene mutations. Programmed-death ligand 1 (PD-L1) is regarded as a crucial biomarker for predicting the efficacy of ICIs in lung cancers^40^. Studies have revealed that heterogeneity in PD-L1 expression levels among independent lesions in MPLC patients^41,42^. However, little is known about the degree of PD-L1 expression between independent pulmonary nodules with *EGFR* or *KRAS* mutations in MPLC patients. Our findings indicate that the expression of *CD274* in *KRAS*_MT tumors was significantly elevated compared to *EGFR*_MT tumors. Moreover, *KRAS*_MT tumors enriched IFN-gamma signature, which has been reported to play an important role in cancer immunotherapy^23^. Taken together, *KRAS*_MT tumors may be more likely to benefit from ICIs than *EGFR*_MT ones. This is in line with a similar study conducted by Dejima et al., which demonstrated that *KRAS*_MT were correlated with a more active immune response, while *EGFR*_MT were associated with a cold immune microenvironment in indeterminate pulmonary nodules^24^. The characteristics of the tumor microenvironment in MPLC patients with distinct driver gene mutation status remain unclear. *KRAS*_MT tumors exhibited a higher infiltration of CD8 + T cells and a higher ratio of Tregs to CD8+ cells compared to *EGFR*_MT tumors. Therefore, future studies should consider the status of driver gene mutations before applying ICIs for the treatment of MPLCs.

The characterization of MPLCs through WES in our study provided molecular evidence supporting the progressive genomic evolution during the transition from pGGO to mGGO, and solid nodules as well as the evolution from normal tissue to AIS, MIA, and IA. Except for TMB, which has been noted to be significantly elevated in LUADs presenting as SNs compared to those with GGO components, and progressively escalating from normal to AIS, further to MIA and IA^43,44^, TNB and chromosomal instability also displayed the similar trend, in accordance with the progression of invasion and the decrease in GGO components. While specific pathways have been identified in certain stages or types of nodules^45,46^, the pathways that consistently contribute to nodule progression have been largely disregarded. Our study sheds light on the crucial role of metabolism-related, cell signaling and cytokine-cytokine receptor interaction, which become increasingly important as the GGO component decreases and cell adhesion and sugar chain biosynthesis become enriched in the high-risk stages. These biological processes represent continuously up-regulated pathways throughout the reduction of GGO and the invasion processes, thus serving as a robust foundation for further clinical investigation. Cells within the TME exhibit significant heterogeneity^47^. Aligning with recent findings^24,30,48,49^, exhausted cells and Tregs are predominantly observed in IA stage, serving as immune suppressive mechanisms during tumor progression. Chen et al. uncovered that GGO-associated lung cancers display a less active metabolism and immune microenvironment compared to lung cancers presenting as SNs^46^. In our study, we observed an upregulation of Treg-related genes and an increased presence of Tregs in SNs, while the number of NK cells was lower in SNs. Therefore, during nodule progression, the TME undergoes varying degrees of remodeling across different nodules, leading to heterogeneity in the TME between nodules. During TME remodeling, cytotoxic cells are significantly decreased, while immunosuppressive cells are markedly increased, thereby providing a suitable environment for nodule solidification and invasion.

This study has certain limitations, such as a small cohort size. Therefore, further experiments involving larger populations are necessary to validate the obtained results.

In conclusion, our study has unveiled significant heterogeneity and distinct origins of MPLCs within the same lung lobe at the genomic level. Throughout the initiation and progression, specific genetic alterations, such as *EGFR* and *KRAS* mutations shape the patterns and characteristics of immune gene expression, leading to varying immune microenvironments and responses to immunotherapy along the trajectory of progression. Concurrently, the increasing mutation burden and chromatin instability contribute to the remodeling of the microenvironment, particularly through the notable elevation of immunosuppressive cells. This process fosters the progression and deterioration of the nodules by establishing a favorable ecosystem.

## Methods

### Ethics Statement

This study was conducted according to the guidelines of the Ethics Committee of the Guangdong Provincial People’s Hospital (No.GDREC2020092H) and written informed consent was obtained from all participants and also complied with all relevant ethical regulations including the declaration of Helsinki.

### Sample collection

Patients diagnosed with MPLCs based on imaging and pathology at Guangdong Provincial People’s Hospital (Guangdong, China) between September 2018 and September 2019 were enrolled in this study. Eligible patients had no signs of distant metastasis based on CT scanning. Nodules were classified into pGGO, mGGO, and solid based on their radiological features on CT imaging. Histological nodule subtypes were defined according to the Lung Cancer/American Thoracic Society/European Respiratory Society (IASLC/ATS/ERS) classification. In brief, the differentiation of lesions is based on the invasive degree using pathological sections stained with hematoxylin and eosin. It is divided into three stages: adenocarcinoma in situ (AIS), minimally invasive adenocarcinoma (MIA), and invasive adenocarcinoma (IA). The lesion, adjacent normal tissue, and matched blood were collected from each patient. All patients signed informed consent.

### Whole-exome sequencing and read alignment

WES was implemented on the paraffin section tissues and matched peripheral blood samples. Mag-Bind Blood&Tissue DNA HDQ 96 Kit (QIAGEN, GER) was performed for fresh sections and blood samples extraction. The dsDNA HS Assay Kit (ThermoFisher Scientific, USA) was used for DNA quantification. Sequencing libraries were built by SureSelect XT Human All Exon V6 (Agilent), and sequencing procedures were utilized by NextSeq 550AR platform with 150-bp paired-end reads. SOAPnuke was implemented to cut adapter and remove low-quality raw reads^50^. Clean reads were aligned against the human reference genome (hg19) with BWA (v0.7.12) and duplicated reads were removed by sambamba (v0.5.4)^51,52^. Subsequently, BAM files were generated and used for downstream analysis.

### Somatic variant calling

We compared nodules, adjacent normal tissue and matched blood sequencing data to identify the somatic mutations, including single-base variants, small insertions, and deletions (indels) using the varscan (v2.4) software^53^. Both substitutions and indels with a low variant allelic fraction (VAF < 0.02) or that had a low total read coverage (<10 reads for tumor samples; < 10 reads for germline samples) were filtered out. In addition, the filtered mutations were annotated by snpEff (v4.3) with NCBIrefseq (https://www.ncbi.nlm.nih.gov/refseq/).

### Tumor mutational burden and tumor neoantigen burden calculation

TMB- was defined by the number of non-synonymous somatic mutation sites examined per megabase. The human leukocyte antigen (HLA) typing of tumor and paired blood sample were determined by POLYSOLVER (v1.0) and Bwakit (v0.7.11)^51,54^. 21-mer polypeptides centered on mutated residues were scanned to identify candidate peptides binding to class I HLAs. A sliding window approach was applied to create 9 ~ 11 mer peptides for major histocompatibility complex (MHC) class I binding affinity prediction. Next, NetMHCpan was performed on the HLA type and the selected peptides to calculate the MHC affinity^55^. Eventually, the peptide with IC50 < 500, which was represented the peptide had a strong binding affinity to patient-specific HLA allele, was selected. TNB was evaluated as the number of neoantigens of exome examined per megabase as previously described^56^.

### Chromosomal instability estimation

Ascatngs (v3.1.0) was implemented on the read alignments from WES data to identify copy number variants, by correcting for GC content, tumor purity and comparing to the matched blood sample. Tumor purity and ploidy were measured by ascatngs as well. Chromosomal instability was evaluated by the Weighted Chromosomal Instability Number^57^.

### Transcriptome sequencing

The mRNA of nodules and adjacent normal samples were isolated using RNeasy Plus Universal Mini Kits (Qiagen, GER) according to the manufacturer’s instructions. The concentrations of RNA were quantified using Qubit^TM^ RNA HS Assay Kit (ThermoFisher Scientific, USA). RNA purity and integrity were checked using Take3 (BioTek, USA) and the RNA Cartridge kit of the Qseq100 Bio-Fragment Analyzer (Bioptic, CHN), respectively. Then, RNA-seq libraries were constructed using VAHTS mRNA-seq V3 Library Prep Kit for Illumina (Vazyme, CHN). The libraries were sequenced on the NextSeq 550AR platform with 150 bp paired-end reads.

### Quality control of RNA-Seq raw data and differential expression genes analysis

Raw sequencing data (raw reads) from Illumina 550AR sequencer was processed to filter out low-quality reads. Clean reads from each sample were obtained and used in the following analysis. The RNA reads were aligned against reference hg19 and gencodev27lift37 database (download from https://www.gencodegenes.org) by STAR (v2.7.8a). Based on the aligned reads, raw counts and transcripts per million (TPM) values were calculated in Rsem (v1.3.0). Then, gene expression level was summarized from the transcript level. Differential expression genes (DEGs) were identified by DESeq2 package^58^. The genes with |log_2_FoldChange | > 1.5 and *p*-value < 0.05 were considered as significantly DEGs. Volcano and heatmap diagram were conducted in R with ggpubr and Complexheatmap package. KEGG pathway enrichment were analyzed on KOBAS-i webtool^59^.

### Immune signature and infiltration abundance of immune cells

A series of immune signatures were implemented to assess tumor immune signatures comprised of cytolytic, IFN-gamma, T-cell, Batf3-DC, and HLA^60^. In brief, each immune feature is scored based on the average expression of specific genes associated with the respective immune feature. Additionally, based on the gene expression matrix, ImmuCellAI and R packages Cibersort were used to estimate the infiltration abundance of immune cells^61,62^.

### multiplex immunofluorescence (mIF) Staining

Samples from 8 independent cohorts were subjected to the mIF staining. The 5 μm-thick slides cut from the FFPE blocks were dewaxed with xylene. Then the slides were rehydrated with a decreasing ethanol series. After rehydrating, the slides were fixed with 10% neutral buffered formalin for 10 min. Next, the slides were stained with markers of CD20, CD56 and Foxp3(akoyo biosciences), followed by incubation with blocking proteins for 10 min. After blocking, the slides were incubated with horseradish peroxidase (HRP)-conjugated secondary antibody and tyramide signal amplification (TSA). Finally, the slides were stained with 4’-6’-diamidino-2-phenylindole (DAPI) for 10 min and images were acquired with vectrapolaris (akoyo biosciences).

### Single-cell RNA-seq data processing

The generation of raw gene expression matrices was performed using CellRanger, followed by analysis using the Seurat R package (v5.0.0)^63^ in R (v4.0.0). Cells expressing less than 300 genes, as well as those with less than 3% ribosomal genes, more than 0.1% hemoglobin genes, and over 20% mitochondrial counts, were removed. Genes expressed in fewer than 3 cells were also excluded. Default parameters of Seurat were utilized, unless otherwise specified. Independent tissue sections’ data were normalized using the SCTransform function of Seurat. Principal component analysis (PCA) was employed for dimensionality reduction and clustering, with a resolution of 0.8 for the first 30 PCAs. The integration of data was achieved through the Seurat functions FindIntegrationAnchors and IntegrateData. Clustering was executed using the FindClusters function, incorporating 30 PCA components and a resolution parameter set to 1.2. Cell types were annotated using known cell-type markers^25^. Differentially expressed genes (DEGs) characterizing the clusters were determined using the default Wilcoxon rank-sum test in the FindAllMarkers function from Seurat, with parameters set to only.pos = TRUE, min.pct = 0.25, and logfc = 0.25. Genes with *p* values below 0.05 were considered differentially expressed.

### Spatial transcriptome data processing

Reads were demultiplexed and mapped to the reference genome GRCh38 using Space Ranger software (v.1.0.0) (10x Genomics). Count matrices were loaded into Seurat for subsequent data filtering, normalization, dimensional reduction, and visualization. Data from independent tissue sections were normalized using the SCTransform function of Seurat. Dimensionality reduction and clustering were performed using PCA at a resolution of 0.8 for the first 30 PCAs. We combined the data of single-cell sequencing as the cell type reference and then applied the ‘anchor’-based integration workflow introduced in Seurat v5 to enable the probabilistic transfer of cell type annotations from a reference to ST data.

### Statistical analysis

All statistical analyses were implemented by R (v4.0.0). Correlations between genes mutation status were assessed by the Spearman’s rank correlation coefficient. The comparison between groups was estimated by two-tailed wilcoxon’s rank-sum test, or two-tailed wilcoxon’s signed-rank test, as appropriate. The Fisher’s exact test was utilized for the category type data. The *p*-value less than 0.05 were regarded as statistical significance. The following convention for symbols indicating statistical significance: ns *p* > 0.05, *p < 0.05, **p < 0.01, ***p < 0.001, ****p < 0.0001. Bar graph represents mean ± standard error of the mean (SEM).

### Supplementary information

Supplementary information

## Data Availability

All data generated or analyzed during this study are included in this published article and its supplementary information files. The raw data generated in this study are available from the corresponding author on reasonable request in the Genome Sequence Archive for Human (GSA-H) repository with accession number (HRA004855). Other data that support the findings of this study can be obtained from the corresponding author upon reasonable request or in the following ways. RNA profiling dataset generated by the NanoString nCounter Platform was obtained from the NCBI Gene Expression Omnibus (GEO) with accession number GSE169033. The scRNA-seq data and the spatial RNA profiling data of KRAS_MT and EGFR_MT lung adenocarcinoma samples generated by our previous study can be obtained from GSA-H with accession number HRA003826. The scRNA-seq and immunohistochemistry data of early lung carcinogenesis samples covering distinct pathological textures can be obtained from the supplementary data of the previous study^30^.
