# Supplementary material for: Multi-omics analysis unveils immunosuppressive microenvironment in the occurrence and development of multiple pulmonary lung cancers
Source: NPJ Precis Oncol. 2024 Jul 23;8:155. doi: 10.1038/s41698-024-00651-5 (PMC11266694; doi:10.1038/s41698-024-00651-5)
Supplement: Supplementary file 1 — Supplementary information [file 41698_2024_651_MOESM1_ESM.pdf]

Supplementary Figures

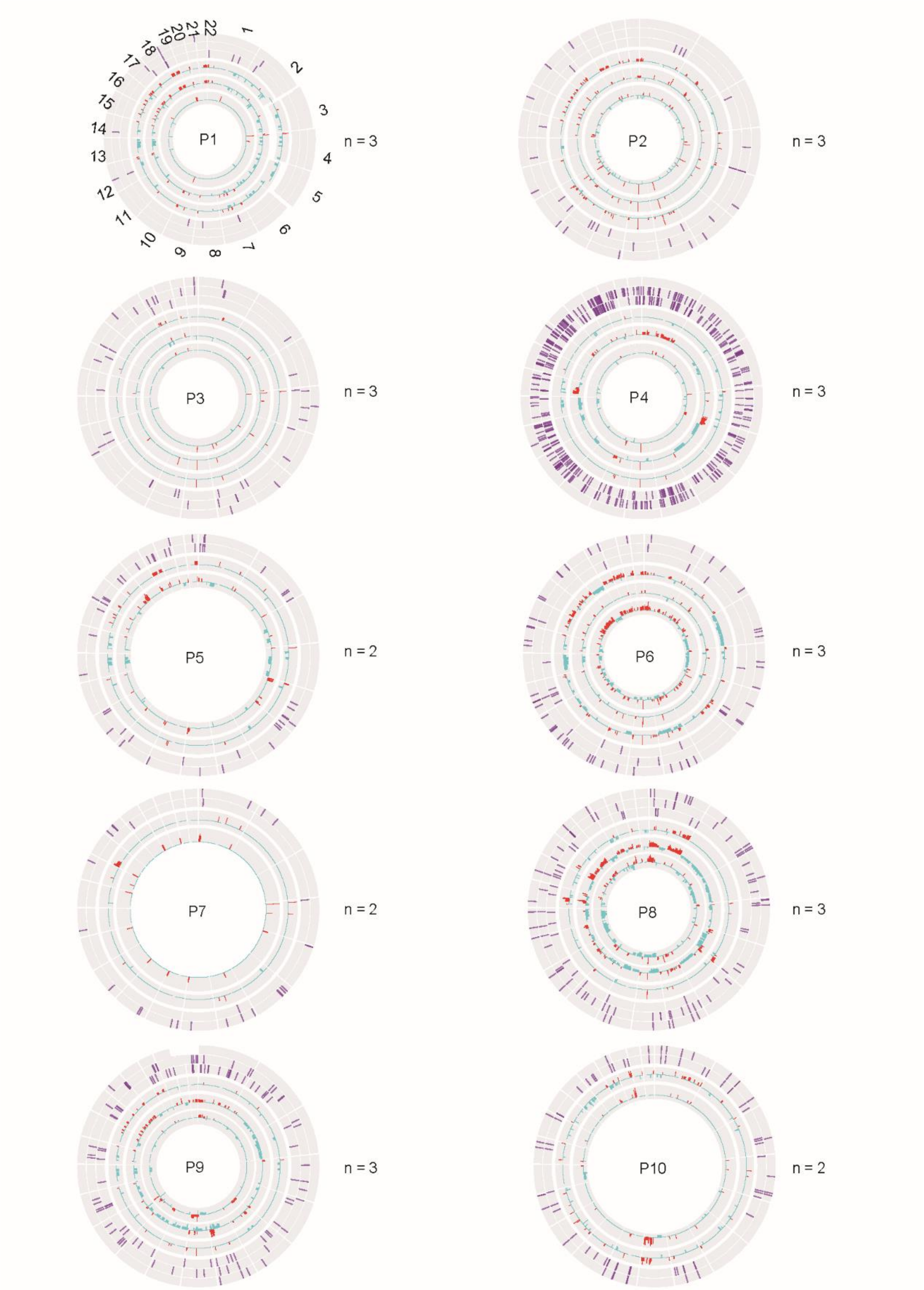

Supplementary Figure 1. Circos diagrams represent the distribution of mutation and copy number variants on autosome for each patient. Number of the cancers was shown on the top. Purple bar in outer tracks represent the mutation variants. Red and blue bars in inner tracks represent the duplications and deletions of segments events on chromosomal, respectively.

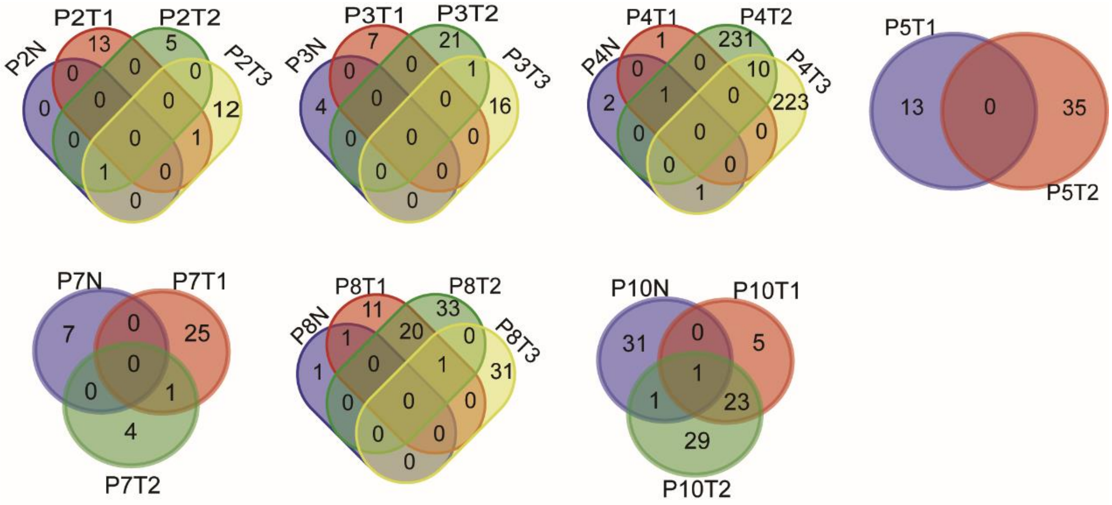

Supplementary Figure 2. Overlapping non-synonymous mutations across samples in each patient.

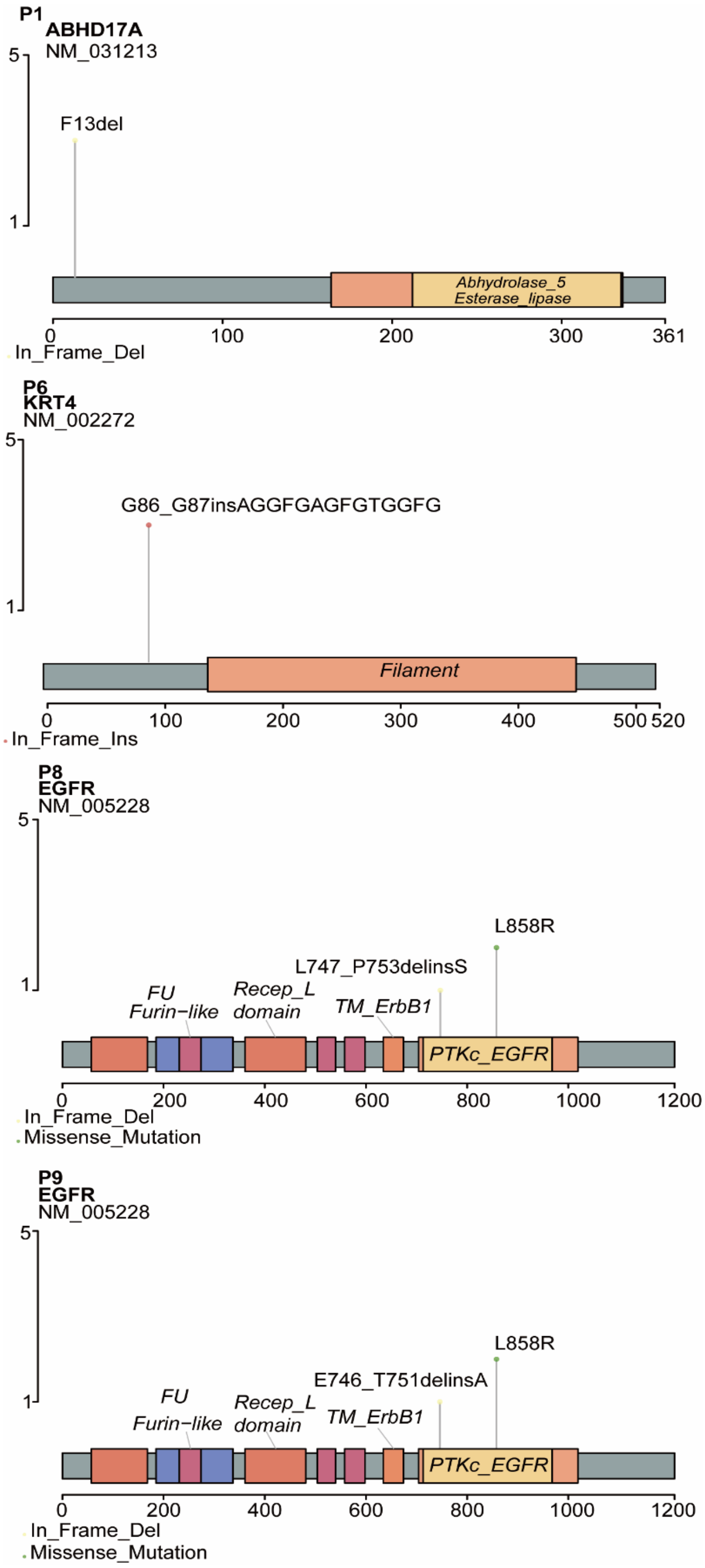

Supplementary Figure 3. The distribution of mutation sites for *ABHD17A*, *KRT4*, and *EGFR* co-mutated loci. The lollipop chart depicts the specific mutation sites and types of genes. The height of the bars represents the number of mutations.

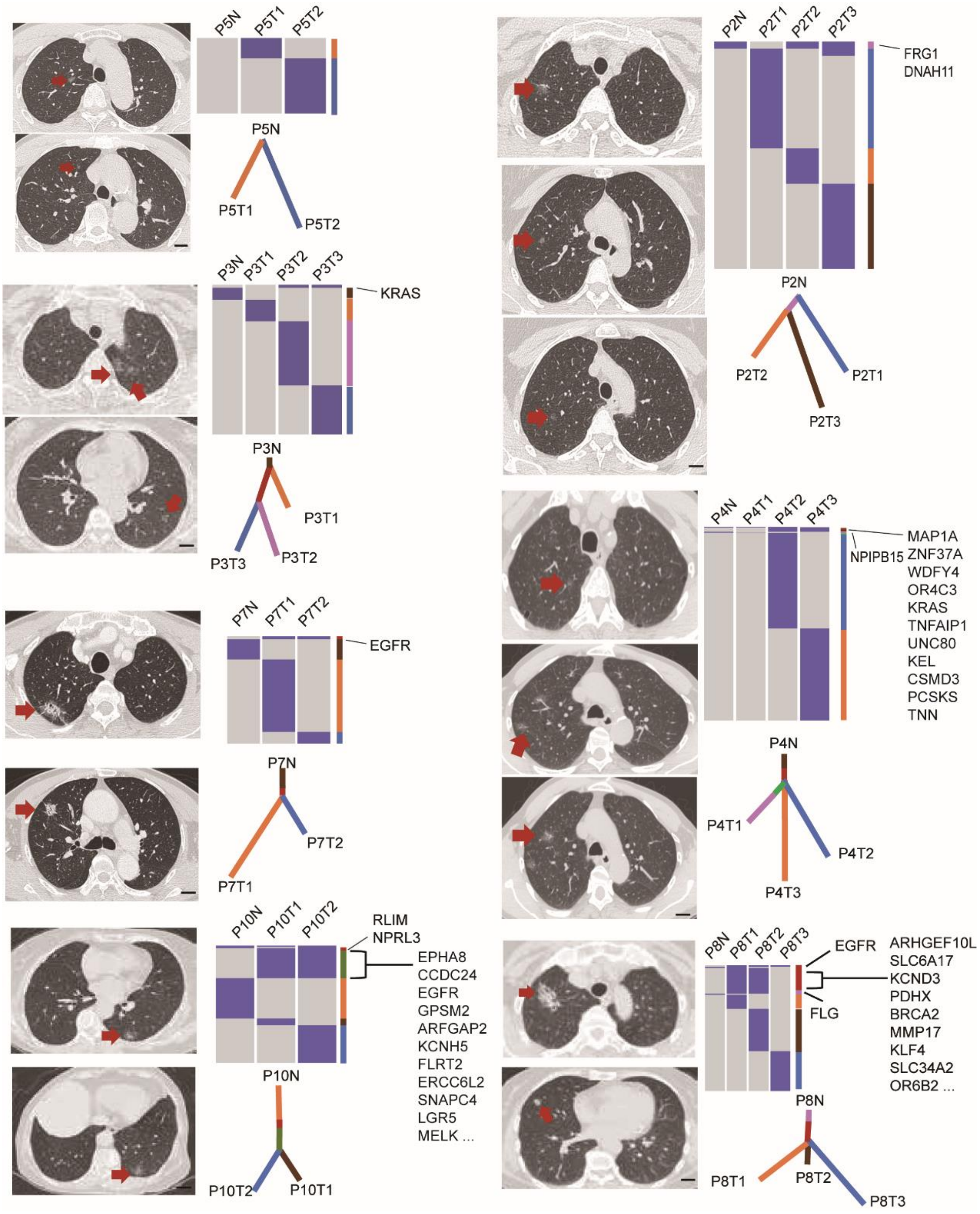

Supplementary Figure 4. CT diagnosis and clonal architecture of multiple pulmonary nodules in patients with MSLCs. CT scans showing multiple pulmonary nodules (arrows) for each patient. Heatmaps in middle panel showed the presence (blue) or absence (gray) of non-synonymous somatic mutations. The overlapping mutation genes were indicated. Phylogenetic trees represented the clonal structure of samples. Scale bar, 1 cm.

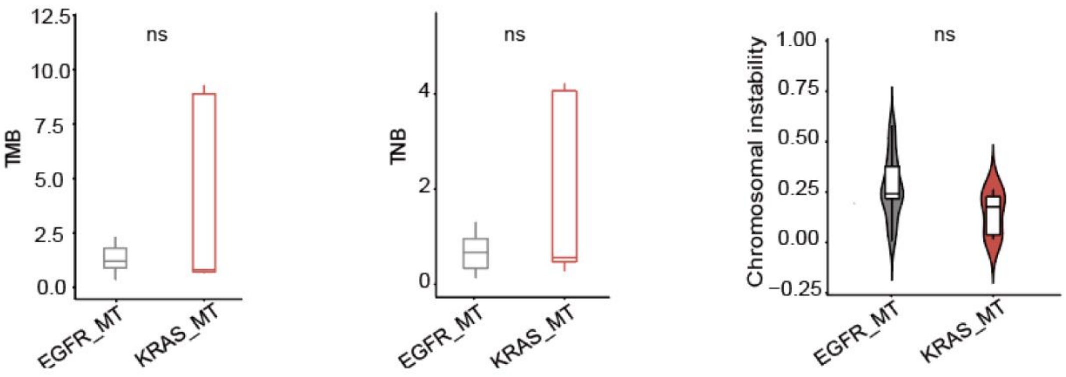

Supplementary Figure 5. Comparison of genomic indicators between *EGFR*_MT and *KRAS*_MT nodules. The comparison of TMB, TNB, Chromosomal instability between EGFR_MT and KRAS_MT groups were shown in boxplot. Centers, boxes, and whiskers indicate medians, quantiles, and minima/maxima, respectively. ns, $p > 0.05$. TMB, tumor mutational burden; TNB, tumor neoantigen burden.

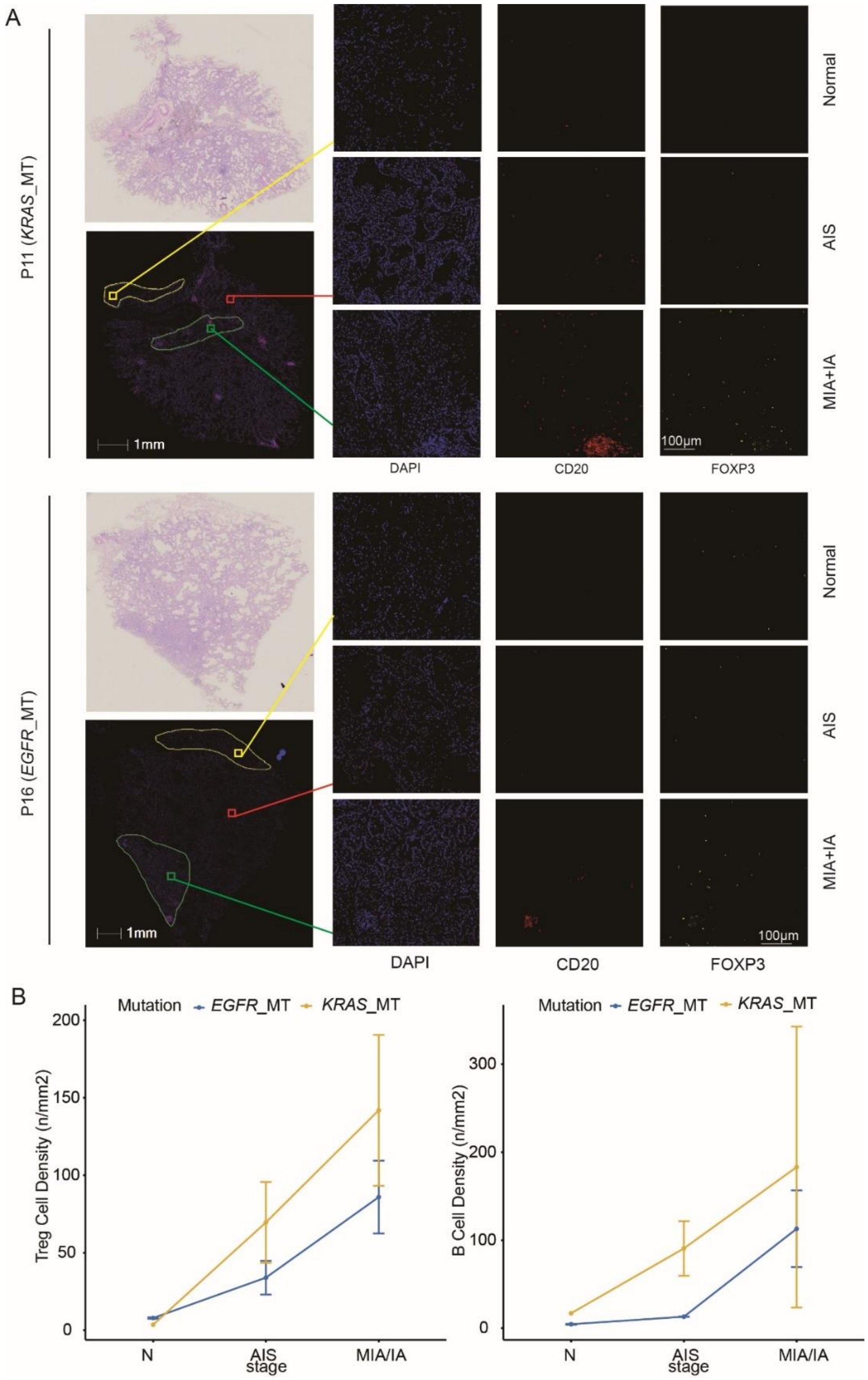

Supplementary Figure 6. Changes of B and Treg density among each stage of MPLCs between *EGFR*_MT and *KRAS*_MT MPLCs. A. Representative images from P11(KRAS_MT) and P16(EGFR_MT) with normal to invasive subregions for HE staining, CD20 B cells (magenta), FOXP3 Treg cells (cyan), and DAPI (blue), as determined by using multiplex immunofluorescence staining. Scale bar, 1mm and 100μm. **B.** Line graphs represent the change of Treg and B cell density across each stage between *EGFR*_MT and *KRAS*_MT MPLCs.

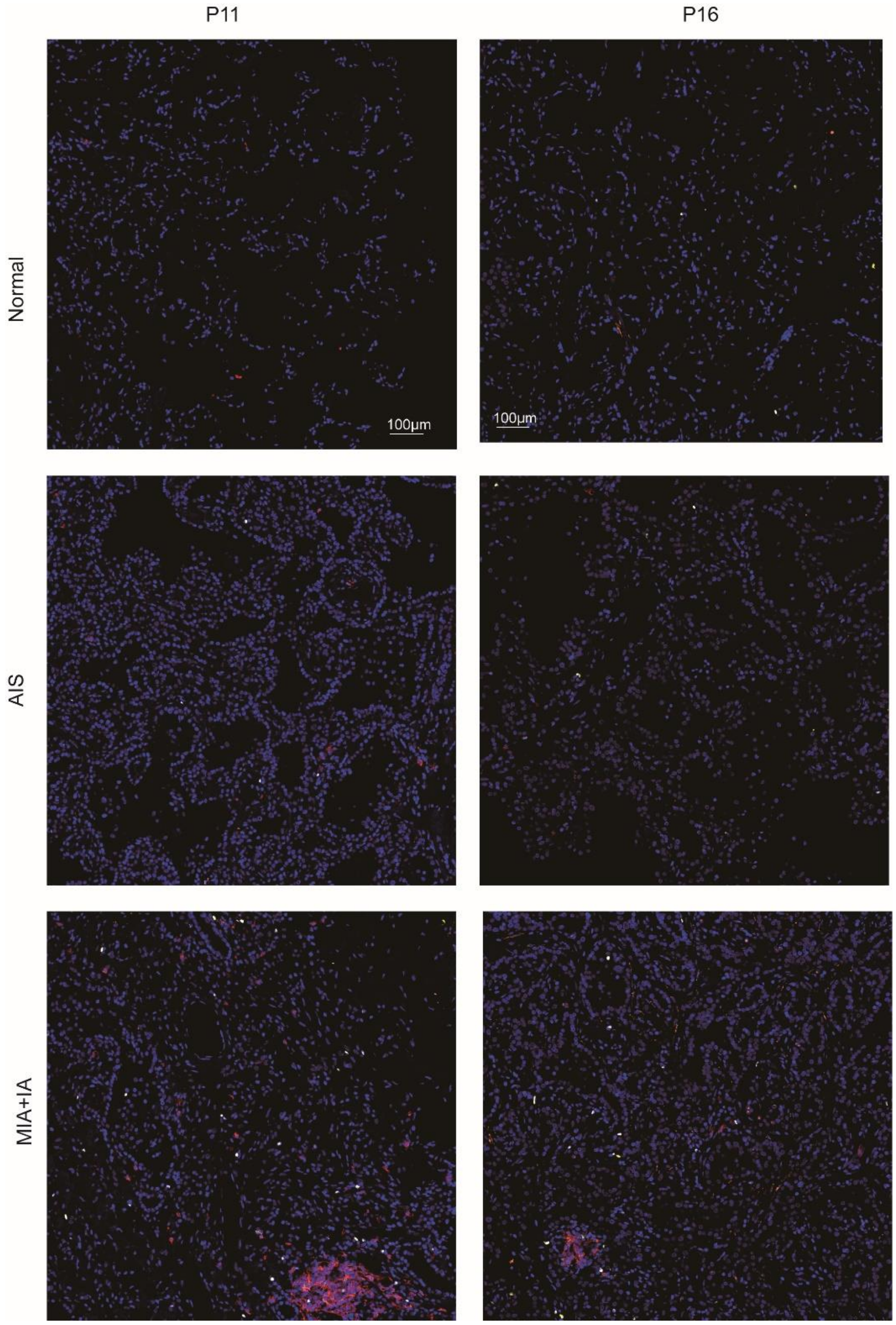

Supplementary Figure 7. Images of P11 (KRAS_MT) and P16 (EGFR_MT) showcasing normal to invasive subregions with merged multiplex immunofluorescence staining. The stains highlight CD20 B cells in magenta, FOXP3 Treg cells in yellow–, and nuclei with DAPI in blue. Scale bars, 1 mm and 100 μm.

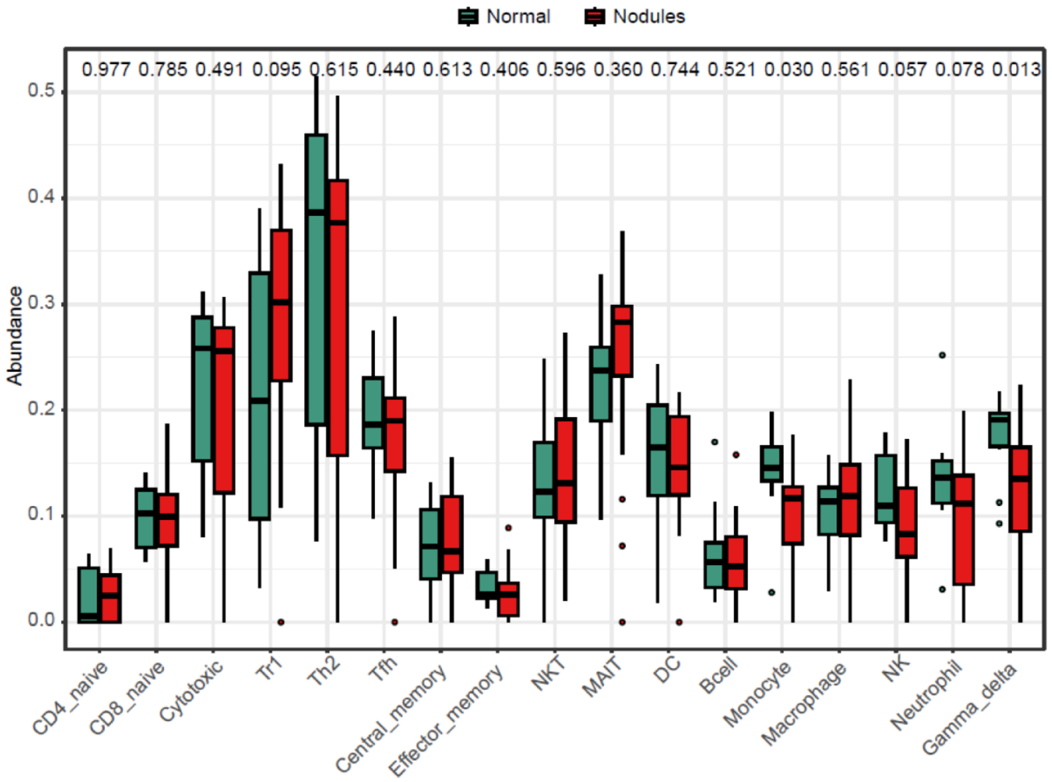

Supplementary Figure 8. The abundance of immune-related cells in nodules versus normal tissues was compared and estimated using ImmunCellAI. Centers, boxes, and whiskers indicate medians, quantiles, and minima/maxima, respectively

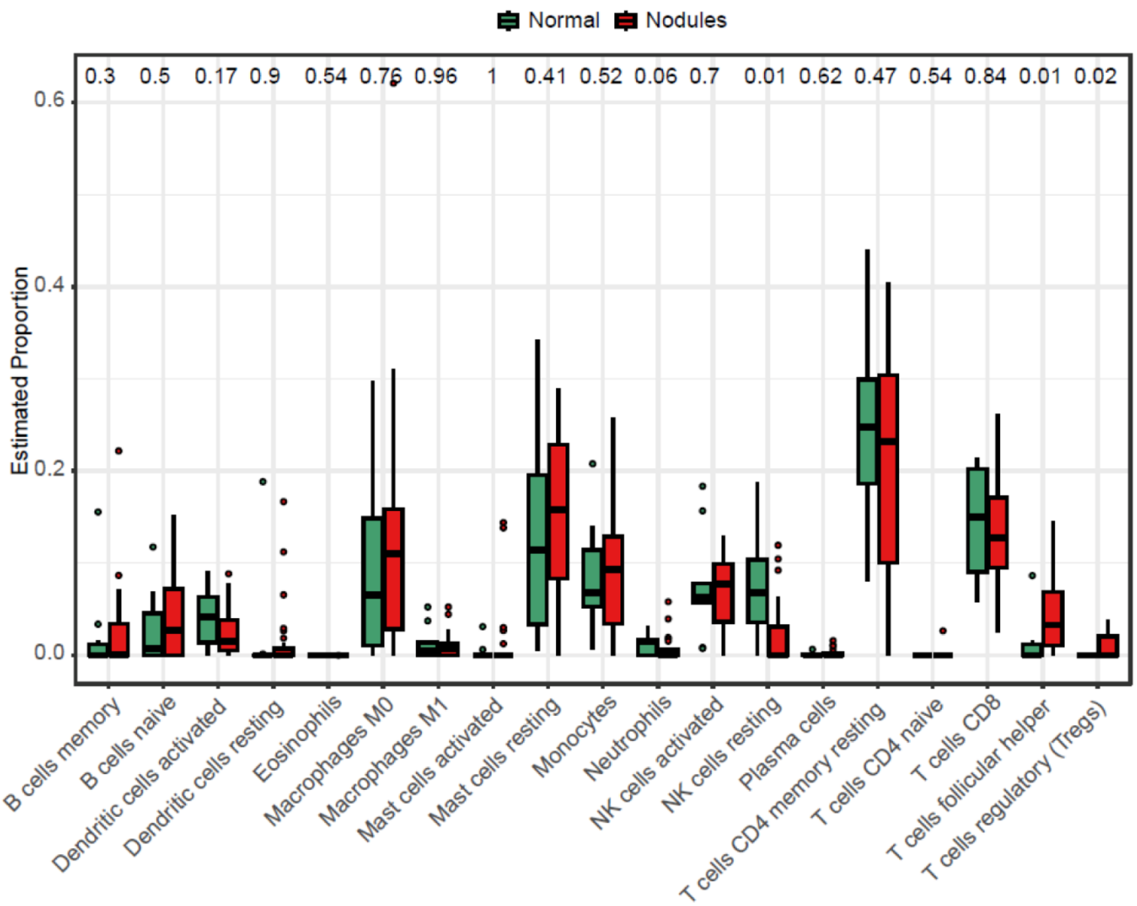

Supplementary Figure 9. The proportion of immune-related cells in nodules versus normal tissues was compared and estimated using Cibersort. Centers, boxes, and whiskers indicate medians, quantiles, and minima/maxima, respectively

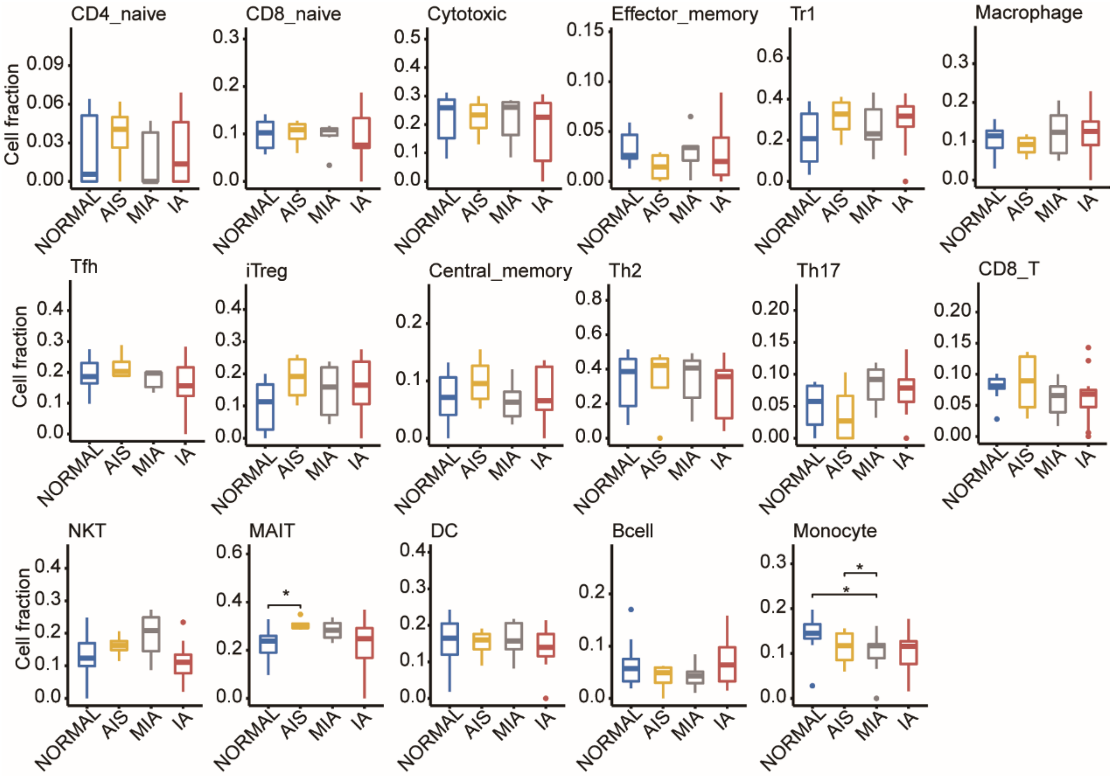

Supplementary Figure 10. Comparison of immune cell populations between each pathological stage. Comparison of distinct immune cell populations among each stage based on pathological classification. Centers, boxes, and whiskers indicate medians, quantiles, and minima/maxima, respectively. *, $p < 0.05$.

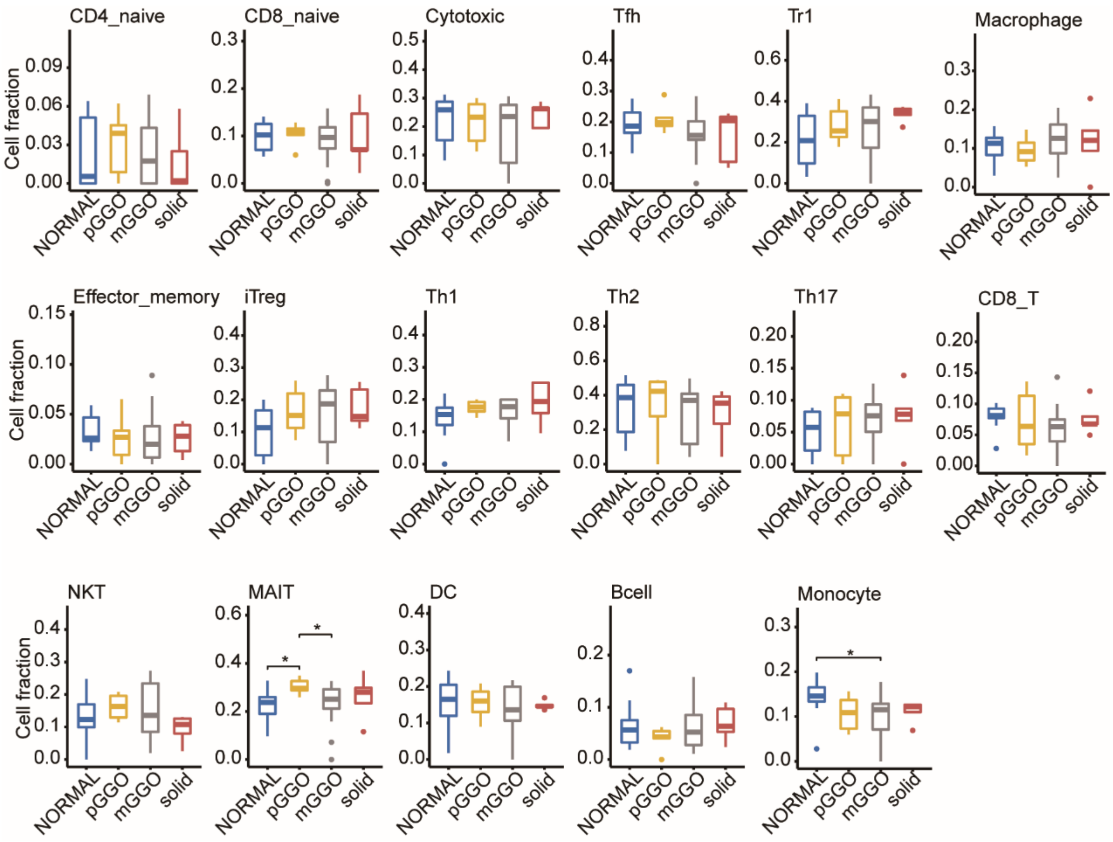

Supplementary Figure 11. Comparison of immune cell populations between each radiological stage. Comparison of distinct immune cell populations among each stage based on radiological classification. Centers, boxes, and whiskers indicate medians, quantiles, and minima/maxima, respectively. *, $p < 0.05$.

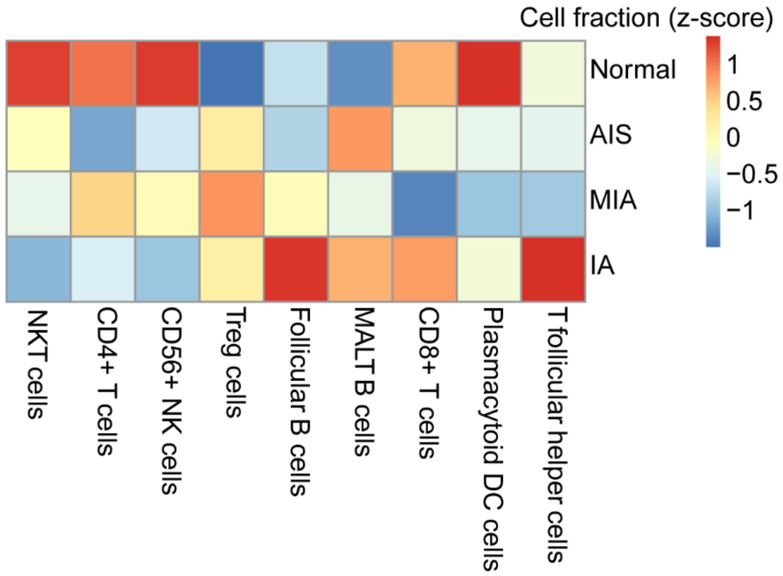

Supplementary Figure 12. Comparison of immune related cell populations among each stage of MPLCs. Cell fraction was normalized (z-scores) to show relative differences among samples.

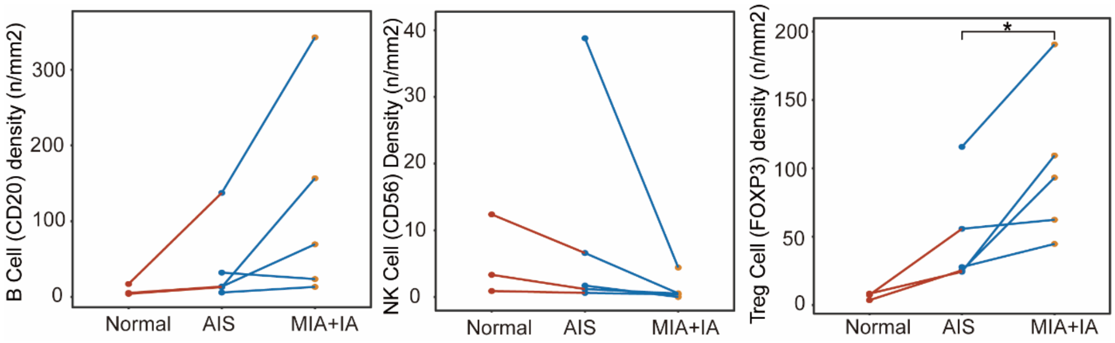

Supplementary Figure 13. Changes of B, NK and Treg cell density among each stage of MPLCs. Comparison of B, NK and Treg cell density among each stage based on mIF staining. mIF, multiplex immunofluorescence; *, $p < 0.05$.

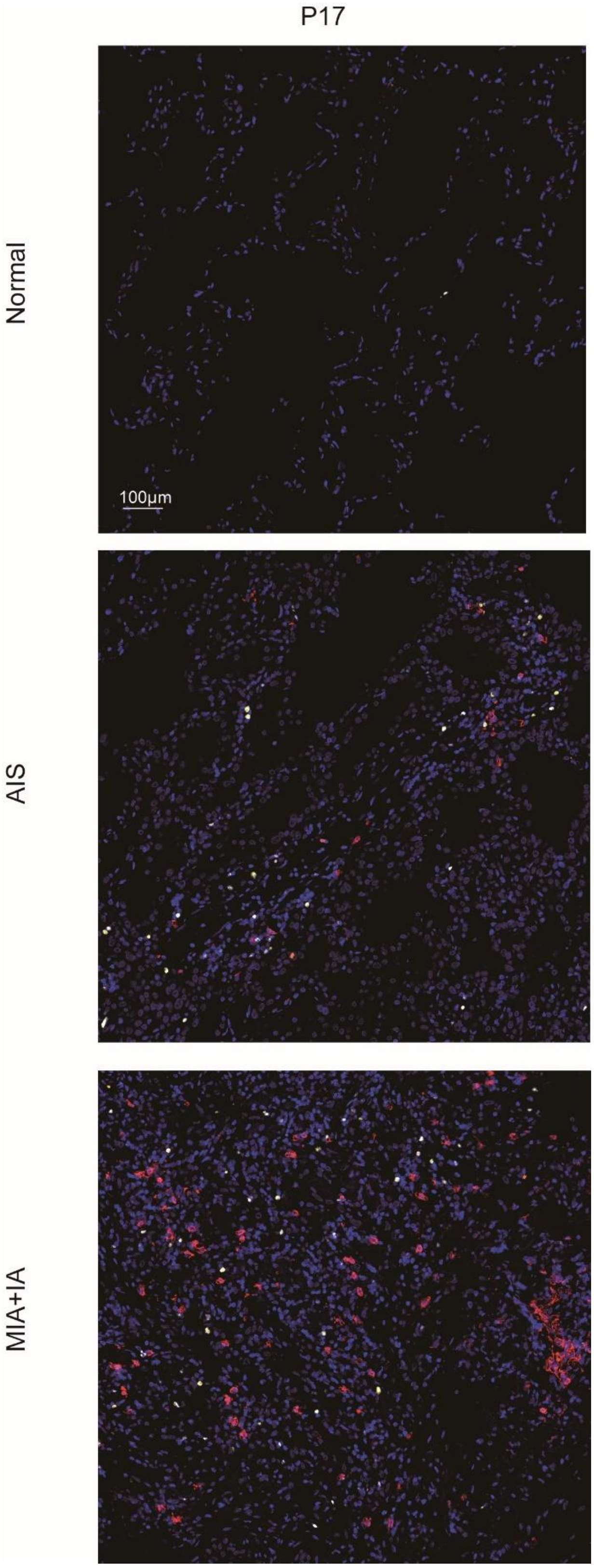

Supplementary Figure 14. Images of P17 showcasing normal to MIA/IA subregions with merged multiplex immunofluorescence staining. The stains highlight CD20 B cells in magenta, FOXP3 Treg cells in yellow, CD56 Macrophage cells in orange, and nuclei with DAPI in blue. Scale bars, 1 mm and 100 μm.

Supplementary Tables

Supplementary Table 1 Characteristics of MPLCs

Sample	TMB	TNB	MSI	Patients	Site	Pathology	Radiology	Size
P10N	1.19	0.56	0.82	P10	adjacent	NORMAL	NORMAL	NA
P10T1	1.35	0.81	0.81	P10	tumor	IA	mGGO	large
P10T2	1.85	0.91	1.65	P10	tumor	IA	mGGO	large
P1N	0.16	0.03	1.65	P1	adjacent	NORMAL	NORMAL	NA
P1T1	0.13	0.03	1.68	P1	tumor	AIS	pGGO	middle
P1T2	0.13	0	1.67	P1	tumor	IA	mGGO	large
P1T3	0.58	0.19	2.52	P1	tumor	IA	solid	large
P2N	0.31	0.03	1.65	P2	adjacent	NORMAL	EGFR_W/KRAS_W	NA
P2T1	0.66	0.28	2.48	P2	tumor	IA	mGGO	large
P2T2	0.41	0.22	1.68	P2	tumor	MIA	pGGO	middle
P2T3	0.69	0.41	1.72	P2	tumor	AIS	pGGO	middle
P3N	0.32	0.16	0.97	P3	adjacent	NORMAL	NORMAL	NA
P3T1	0.31	0.16	0.9	P3	tumor	MIA	mGGO	large
P3T2	0.81	0.56	2.7	P3	tumor	MIA	mGGO	large
P3T3	0.72	0.47	0	P3	tumor	MIA	mGGO	large
P4N	0.38	0.09	1.34	P4	adjacent	NORMAL	NORMAL	NA
P4T1	0.28	0.06	0.68	P4	tumor	AIS	pGGO	middle
P4T2	9.29	4.22	1.37	P4	tumor	MIA	pGGO	large
P4T3	8.88	4.07	2.74	P4	tumor	IA	mGGO	large
P5N	0.06	0	0.88	P5	adjacent	NORMAL	NORMAL	NA
P5T1	0.53	0.16	0.9	P5	tumor	IA	mGGO	large
P5T2	1.19	0.53	0	P5	tumor	MIA	mGGO	middle
P6N	0.28	0.22	1.44	P6	adjacent	NORMAL	NORMAL	NA
P6T1	2.03	0.97	0.74	P6	tumor	MIA	mGGO	large
P6T2	0.66	0.19	1.46	P6	tumor	AIS	pGGO	middle
P6T3	0.97	0.41	4.38	P6	tumor	IA	mGGO	large
P7N	0.22	0.03	0.72	P7	adjacent	NORMAL	NORMAL	NA
P7T1	0.88	0.31	1.48	P7	tumor	IA	mGGO	large
P7T2	0.34	0.13	1.47	P7	tumor	IA	solid	large
P8N	0.09	0	0	P8	adjacent	NORMAL	NORMAL	NA
P8T1	1.22	0.78	2.68	P8	tumor	IA	solid	large
P8T2	1.82	1.28	0.89	P8	tumor	IA	solid	large
P8T3	1	0.56	0.89	P8	tumor	IA	solid	large
P9N	0.19	0.09	3.79	P9	adjacent	NORMAL	NORMAL	NA
P9T1	0.44	0.28	1.5	P9	tumor	IA	mGGO	large
P9T2	1.75	1.06	1.5	P9	tumor	IA	mGGO	large
P9T3	2.31	1.31	3.03	P9	tumor	IA	mGGO	large

Supplementary Table 2 List of somatic mutations.

Symbol	Chr	End	Variant_Classification	Reference	Tumor	Sample
SIX2	chr2	45233332	Missense_Mutation	T	G	P1T1
PABPC3	chr13	25671451	Missense_Mutation	A	G	P1T1
PABPC3	chr13	25671456	Missense_Mutation	C	T	P1T1
SLC7A8	chr14	23612372	Missense_Mutation	T	G	P1T1
PGS1	chr17	76399863	Missense_Mutation	T	G	P1T1
ABHD17A	chr19	1881528	In_Frame_Del	AGA	-	P1T1
SIX2	chr2	45233332	Missense_Mutation	T	G	P1T2
PABPC3	chr13	25671451	Missense_Mutation	A	G	P1T2
PABPC3	chr13	25671456	Missense_Mutation	C	T	P1T2
SLC7A8	chr14	23612372	Missense_Mutation	T	G	P1T2
PGS1	chr17	76399863	Missense_Mutation	T	G	P1T2
ABHD17A	chr19	1881528	In_Frame_Del	AGA	-	P1T2
HSPA6	chr1	161495788	Missense_Mutation	G	T	P1N
SLC9A4	chr2	103095615	Missense_Mutation	A	C	P1N
PDE1B	chr12	54968998	Missense_Mutation	G	A	P1N
ABHD17A	chr19	1881528	In_Frame_Del	AGA	-	P1N
CFAP74	chr1	1878458	Nonsense_Mutation	C	A	P1T3
LAMC2	chr1	183192273	Missense_Mutation	A	G	P1T3
GALNT14	chr2	31360922	Missense_Mutation	G	A	P1T3
EGFR	chr7	55241654	Missense_Mutation	A	T	P1T3
EGFR	chr7	55259515	Missense_Mutation	T	G	P1T3
ODF1	chr8	103572804	Missense_Mutation	T	G	P1T3
ODF1	chr8	103572806	Missense_Mutation	T	G	P1T3
TAF1L	chr9	32634722	Missense_Mutation	G	A	P1T3
PDE1B	chr12	54968998	Missense_Mutation	G	A	P1T3
MYL12B	chr18	3272946	Missense_Mutation	G	A	P1T3
DPP9	chr19	4703957	Missense_Mutation	T	A	P1T3
LILRA5	chr19	54823843	Missense_Mutation	C	T	P1T3
NUDT17	chr1	145588611	Splice_Site	CCT	-	P1T3
ABHD17A	chr19	1881528	In_Frame_Del	AGA	-	P1T3
FRG1	chr4	190876268	Missense_Mutation	A	G	P2N
FRG1	chr4	190876272	Missense_Mutation	G	A	P2N
ARHGAP26	chr5	142602010	Nonstop_Mutation	A	C	P2T1
VNN3	chr6	133045855	Missense_Mutation	C	G	P2T1
DNAH11	chr7	21640477	Missense_Mutation	G	A	P2T1
KIAA2026	chr9	5920360	Missense_Mutation	T	C	P2T1
CFAP58	chr10	106128205	Nonsense_Mutation	G	T	P2T1
MUC6	chr11	1016871	Missense_Mutation	G	T	P2T1
KRAS	chr12	25398285	Missense_Mutation	C	T	P2T1
FBN1	chr15	48755323	Missense_Mutation	C	T	P2T1

ZNF469	chr16	88499973	Missense_Mutation	G	A	P2T1
NOL4	chr18	31599388	Missense_Mutation	G	A	P2T1
MAGEB2	chrX	30237347	Nonsense_Mutation	C	G	P2T1
ARAF	chrX	47426131	Missense_Mutation	C	A	P2T1
TEX13A	chrX	104464912	Missense_Mutation	C	T	P2T1
XPNPEP2	chrX	128890496	Missense_Mutation	T	G	P2T1
GK2	chr4	80329070	Missense_Mutation	T	G	P2T2
GK2	chr4	80329071	Missense_Mutation	T	G	P2T2
FRG1	chr4	190876268	Missense_Mutation	A	G	P2T2
FRG1	chr4	190876272	Missense_Mutation	G	A	P2T2
KHDRBS2	chr6	62390889	Missense_Mutation	T	G	P2T2
RELN	chr7	103301862	Missense_Mutation	T	G	P2T2
ODF1	chr8	103572804	Missense_Mutation	T	G	P2T2
ODF1	chr8	103572806	Missense_Mutation	T	G	P2T2
ATP12A	chr13	25281501	Missense_Mutation	A	C	P2T2
BRINP2	chr1	177249783	Missense_Mutation	T	G	P2T3
MAPKAPK2	chr1	206902158	Missense_Mutation	A	T	P2T3
OBSCN	chr1	228431153	Missense_Mutation	A	G	P2T3
SCN5A	chr3	38655464	Splice_Site	A	G	P2T3
SYNPR	chr3	63264210	Translation_Start_Site	A	T	P2T3
FRG1	chr4	190876268	Missense_Mutation	A	G	P2T3
FRG1	chr4	190876272	Missense_Mutation	G	A	P2T3
DNAH11	chr7	21640477	Missense_Mutation	G	A	P2T3
APBA1	chr9	72056016	Missense_Mutation	A	G	P2T3
LHX3	chr9	139091668	Missense_Mutation	G	A	P2T3
OPTN	chr10	13175546	Missense_Mutation	G	T	P2T3
LYZL1	chr10	29580887	Missense_Mutation	T	A	P2T3
RRAS2	chr11	14316390	Missense_Mutation	T	A	P2T3
OMP	chr11	76814291	Missense_Mutation	C	T	P2T3
DNAAF2	chr14	50101684	Missense_Mutation	C	T	P2T3
OR4D1	chr17	56232867	Missense_Mutation	T	A	P2T3
TMEM131	chr2	98392364	Missense_Mutation	T	C	P3N
C17orf107	chr17	4803259	Missense_Mutation	C	A	P3N
CPXM1	chr20	2778856	Missense_Mutation	C	A	P3N
CCR10	chr17	40832034	Frame_Shift_Del	CGCCA	-	P3N
GK2	chr4	80329070	Missense_Mutation	T	G	P3T1
KHDRBS2	chr6	62390889	Missense_Mutation	T	G	P3T1
RELN	chr7	103301862	Missense_Mutation	T	G	P3T1
A2ML1	chr12	9008180	Missense_Mutation	C	T	P3T1
KRTAP1-1	chr17	39197618	Missense_Mutation	A	T	P3T1
EIF3D	chr22	36920678	Missense_Mutation	T	A	P3T1
DPYD	chr1	97771791	Frame_Shift_Del	A	-	P3T1
CEPT1	chr1	111724899	Missense_Mutation	A	G	P3T2
ATP1A1	chr1	116939230	Missense_Mutation	T	C	P3T2

PNKD	chr2	219206298	Missense_Mutation	G	A	P3T2
BOD1L1	chr4	13602675	Missense_Mutation	G	A	P3T2
FAM13A	chr4	89653154	Missense_Mutation	T	C	P3T2
ZMAT2	chr5	140081596	Missense_Mutation	C	T	P3T2
ZNF679	chr7	63726865	Missense_Mutation	A	C	P3T2
ZNF679	chr7	63727170	Nonsense_Mutation	A	T	P3T2
HAS2	chr8	122626810	Missense_Mutation	G	A	P3T2
KRAS	chr12	25398284	Missense_Mutation	C	A	P3T2
STARD9	chr15	42985540	Missense_Mutation	G	A	P3T2
ZSCAN30	chr18	32834049	Missense_Mutation	T	C	P3T2
TIMM50	chr19	39978771	Missense_Mutation	T	G	P3T2
ZNF865	chr19	56126984	Missense_Mutation	T	G	P3T2
KRTAP13-1	chr21	31768853	Missense_Mutation	C	A	P3T2
KCTD17	chr22	37456867	Missense_Mutation	C	T	P3T2
CNKSR2	chrX	21627252	Nonsense_Mutation	C	T	P3T2
FGD1	chrX	54496709	Missense_Mutation	G	A	P3T2
CDX4	chrX	72667444	Missense_Mutation	G	A	P3T2
ABCE1	chr4	146042492	Frame_Shift_Ins	-	A	P3T2
SRRM2	chr16	2819050	Frame_Shift_Ins	-	C	P3T2
ZNF20	chr19	12244370	Frame_Shift_Del	G	-	P3T2
EPHB1	chr3	134670282	Missense_Mutation	G	A	P3T3
WFS1	chr4	6302888	Missense_Mutation	C	T	P3T3
WDR27	chr6	170064268	Nonsense_Mutation	G	A	P3T3
TSPAN13	chr7	16816672	Missense_Mutation	G	T	P3T3
SAXO1	chr9	18928264	Missense_Mutation	C	T	P3T3
GLIPR2	chr9	36148575	Missense_Mutation	C	T	P3T3
FAM171A1	chr10	15256445	Missense_Mutation	C	T	P3T3
CUBN	chr10	16996472	Missense_Mutation	C	T	P3T3
KRAS	chr12	25398284	Missense_Mutation	C	A	P3T3
RAB3IP	chr12	70150303	Missense_Mutation	A	G	P3T3
EMC9	chr14	24608638	Missense_Mutation	T	A	P3T3
SCAMP5	chr15	75308988	Missense_Mutation	G	T	P3T3
ADCY9	chr16	4039058	Missense_Mutation	C	G	P3T3
RBPJL	chr20	43942234	Missense_Mutation	C	T	P3T3
AHRR	chr5	434520	Frame_Shift_Del	G	-	P3T3
BRAF	chr7	140453146	In_Frame_Del	AGCTAG	-	P3T3
BRD4	chr19	15383774	Frame_Shift_Del	G	-	P3T3
NEURL1	chr10	105344974	Missense_Mutation	G	A	P4N
SLC7A8	chr14	23612372	Missense_Mutation	T	G	P4N
MAP1A	chr15	43814170	Missense_Mutation	A	C	P4N
NPIPB15	chr16	74425961	Missense_Mutation	G	A	P4N
TROAP	chr12	49724412	Missense_Mutation	T	G	P4T1
NPIPB15	chr16	74425961	Missense_Mutation	G	A	P4T1
KLHL17	chr1	898192	Nonsense_Mutation	G	T	P4T2

KDM1A	chr1	23409832	Missense_Mutation	C	T	P4T2
PTAFR	chr1	28477001	Nonsense_Mutation	C	A	P4T2
COL16A1	chr1	32145420	Missense_Mutation	C	A	P4T2
SPOCD1	chr1	32256255	Missense_Mutation	C	A	P4T2
FOXE3	chr1	47882788	Missense_Mutation	C	A	P4T2
PTGER3	chr1	71477993	Missense_Mutation	A	C	P4T2
PTGER3	chr1	71478008	Missense_Mutation	G	T	P4T2
LRRIQ3	chr1	74507378	Missense_Mutation	C	G	P4T2
ERICH3	chr1	75038789	Missense_Mutation	C	G	P4T2
PHGDH	chr1	120283139	Missense_Mutation	A	G	P4T2
PDE4DIP	chr1	144909913	Missense_Mutation	T	C	P4T2
TMOD4	chr1	151143521	Missense_Mutation	G	A	P4T2
FLG	chr1	152285536	Missense_Mutation	G	A	P4T2
NOS1AP	chr1	162326769	Missense_Mutation	C	G	P4T2
PRRX1	chr1	170695539	Missense_Mutation	A	T	P4T2
CACNA1E	chr1	181767688	Missense_Mutation	A	T	P4T2
LGR6	chr1	202287470	Missense_Mutation	C	A	P4T2
LEFTY1	chr1	226074697	Nonsense_Mutation	C	T	P4T2
OBSCN	chr1	228504655	Missense_Mutation	G	A	P4T2
VSNL1	chr2	17830798	Missense_Mutation	G	A	P4T2
TTC27	chr2	32961848	Missense_Mutation	G	T	P4T2
REG1B	chr2	79312666	Missense_Mutation	G	C	P4T2
SMYD1	chr2	88387501	Missense_Mutation	G	T	P4T2
SH3RF3	chr2	109988132	Missense_Mutation	G	T	P4T2
CNTNAP5	chr2	125175149	Missense_Mutation	G	A	P4T2
PDE11A	chr2	178682623	Missense_Mutation	C	A	P4T2
MDH1B	chr2	207619888	Missense_Mutation	C	A	P4T2
UNC80	chr2	210683861	Missense_Mutation	T	A	P4T2
DNER	chr2	230411741	Missense_Mutation	G	C	P4T2
TRPM8	chr2	234851373	Missense_Mutation	T	A	P4T2
SLC6A1	chr3	11067528	Missense_Mutation	G	C	P4T2
CLASP2	chr3	33686375	Missense_Mutation	C	G	P4T2
STAC	chr3	36587768	Missense_Mutation	T	C	P4T2
MOBP	chr3	39543721	Missense_Mutation	A	T	P4T2
MOBP	chr3	39544337	Missense_Mutation	G	C	P4T2
MYRIP	chr3	40208418	Missense_Mutation	C	A	P4T2
CDCP1	chr3	45152249	Missense_Mutation	T	A	P4T2
RTP3	chr3	46542129	Missense_Mutation	G	T	P4T2
USP4	chr3	49335370	Nonsense_Mutation	G	A	P4T2
USF3	chr3	113378826	Missense_Mutation	C	A	P4T2
STXBP5L	chr3	120764281	Splice_Site	G	T	P4T2
COL6A5	chr3	130107734	Missense_Mutation	C	A	P4T2
MINDY4B	chr3	150608141	Missense_Mutation	G	T	P4T2
CCDC39	chr3	180332845	Missense_Mutation	G	A	P4T2

VWA5B2	chr3	183951028	Missense_Mutation	G	A	P4T2
BCL6	chr3	187447414	Missense_Mutation	G	A	P4T2
MUC4	chr3	195516845	Missense_Mutation	C	A	P4T2
BLOC1S4	chr4	6718096	Missense_Mutation	C	G	P4T2
GABRA2	chr4	46252537	Missense_Mutation	G	T	P4T2
TXK	chr4	48091804	Missense_Mutation	C	A	P4T2
NAA11	chr4	80246523	Missense_Mutation	A	C	P4T2
PDHA2	chr4	96762008	Missense_Mutation	C	A	P4T2
MTTP	chr4	100532383	Missense_Mutation	C	A	P4T2
DNAJB14	chr4	100825027	Missense_Mutation	T	C	P4T2
PCDH10	chr4	134084130	Splice_Site	A	T	P4T2
GYPE	chr4	144801575	Nonsense_Mutation	G	C	P4T2
ADAM29	chr4	175897169	Nonsense_Mutation	G	T	P4T2
DROSHA	chr5	31485000	Missense_Mutation	A	T	P4T2
SELENOP	chr5	42801099	Missense_Mutation	G	A	P4T2
SKIV2L2	chr5	54639174	Missense_Mutation	G	T	P4T2
POLK	chr5	74807740	Splice_Site	G	T	P4T2
ELL2	chr5	95234154	Missense_Mutation	G	A	P4T2
FAT2	chr5	150942990	Missense_Mutation	A	T	P4T2
CNOT8	chr5	154252099	Splice_Site	A	T	P4T2
ATP10B	chr5	160114838	Missense_Mutation	G	T	P4T2
FAM196B	chr5	169310346	Missense_Mutation	C	T	P4T2
HIST1H4C	chr6	26104216	Missense_Mutation	G	A	P4T2
LRFN2	chr6	40360031	Missense_Mutation	A	T	P4T2
LRFN2	chr6	40399786	Missense_Mutation	G	T	P4T2
PEX6	chr6	42936653	Missense_Mutation	C	T	P4T2
DST	chr6	56462547	Missense_Mutation	C	A	P4T2
CD109	chr6	74519787	Missense_Mutation	G	T	P4T2
TNRC18	chr7	5352712	Missense_Mutation	C	A	P4T2
NEUROD6	chr7	31378196	Missense_Mutation	C	A	P4T2
ADCY1	chr7	45662328	Missense_Mutation	G	A	P4T2
ZNF479	chr7	57188832	Missense_Mutation	A	T	P4T2
HGF	chr7	81386604	Missense_Mutation	C	A	P4T2
SAMD9	chr7	92733037	Missense_Mutation	C	G	P4T2
COL1A2	chr7	94049703	Missense_Mutation	G	T	P4T2
CNPY4	chr7	99717390	Missense_Mutation	T	C	P4T2
MUC3A	chr7	100609804	Splice_Site	G	T	P4T2
LAMB4	chr7	107746393	Missense_Mutation	C	T	P4T2
NRCAM	chr7	107820799	Missense_Mutation	G	T	P4T2
MGAM	chr7	141722196	Missense_Mutation	G	T	P4T2
KEL	chr7	142640936	Missense_Mutation	A	T	P4T2
KCNH2	chr7	150646110	Missense_Mutation	A	T	P4T2
PTPRN2	chr7	157985140	Missense_Mutation	C	T	P4T2
UNC5D	chr8	35544089	Nonsense_Mutation	G	T	P4T2

UNC5D	chr8	35544093	Missense_Mutation	G	T	P4T2
SNTG1	chr8	51314885	Missense_Mutation	G	C	P4T2
CHD7	chr8	61773535	Nonsense_Mutation	G	T	P4T2
TERF1	chr8	73921261	Missense_Mutation	G	A	P4T2
ZFHX4	chr8	77618013	Missense_Mutation	G	A	P4T2
KCNS2	chr8	99440720	Missense_Mutation	G	C	P4T2
UBR5	chr8	103282310	Missense_Mutation	G	T	P4T2
ODF1	chr8	103572804	Missense_Mutation	T	G	P4T2
CSMD3	chr8	113697832	Missense_Mutation	G	A	P4T2
CYP11B1	chr8	143957676	Missense_Mutation	G	T	P4T2
PTPRD	chr9	8376648	Nonsense_Mutation	C	A	P4T2
ADAMTSL1	chr9	18504835	Missense_Mutation	G	T	P4T2
TESK1	chr9	35606008	Missense_Mutation	A	T	P4T2
ZNF658	chr9	40773185	Missense_Mutation	T	C	P4T2
TRPM3	chr9	73151179	Missense_Mutation	C	A	P4T2
PCSK5	chr9	78710939	Missense_Mutation	A	G	P4T2
BRINP1	chr9	121976359	Missense_Mutation	C	G	P4T2
TSC1	chr9	135787782	Missense_Mutation	G	A	P4T2
FCN2	chr9	137774401	Missense_Mutation	G	A	P4T2
ZNF37A	chr10	38407065	Missense_Mutation	G	A	P4T2
FRMPD2	chr10	49392886	Missense_Mutation	C	A	P4T2
WDFY4	chr10	49982603	Missense_Mutation	C	T	P4T2
A1CF	chr10	52603845	Missense_Mutation	C	A	P4T2
DCLRE1A	chr10	115610039	Missense_Mutation	C	A	P4T2
EIF3A	chr10	120803654	Missense_Mutation	C	A	P4T2
GPR26	chr10	125447528	Missense_Mutation	C	G	P4T2
MUC2	chr11	1090898	Missense_Mutation	C	A	P4T2
OR56A3	chr11	5968729	Missense_Mutation	G	T	P4T2
SLC17A6	chr11	22364809	Missense_Mutation	G	T	P4T2
OR4C3	chr11	48347393	Missense_Mutation	G	T	P4T2
OR5AS1	chr11	55798105	Missense_Mutation	A	T	P4T2
OR8J1	chr11	56127964	Missense_Mutation	T	A	P4T2
APLNR	chr11	57003874	Missense_Mutation	C	A	P4T2
OR10Q1	chr11	57995804	Missense_Mutation	A	T	P4T2
GLYAT	chr11	58477505	Missense_Mutation	G	T	P4T2
VPS37C	chr11	60899609	Nonsense_Mutation	C	A	P4T2
CDC42BPG	chr11	64603898	Splice_Site	C	T	P4T2
SYT12	chr11	66811296	Missense_Mutation	G	T	P4T2
CAPN5	chr11	76830198	Missense_Mutation	G	T	P4T2
BIRC3	chr11	102195794	Missense_Mutation	C	T	P4T2
ROBO4	chr11	124765572	Missense_Mutation	G	T	P4T2
ACRBP	chr12	6754496	Missense_Mutation	A	T	P4T2
CD4	chr12	6909312	Missense_Mutation	G	A	P4T2
CD163	chr12	7653965	Missense_Mutation	C	A	P4T2

A2M	chr12	9259201	Missense_Mutation	G	T	P4T2
KRAS	chr12	25398285	Missense_Mutation	C	A	P4T2
RAB5B	chr12	56383790	Missense_Mutation	G	C	P4T2
PPM1H	chr12	63060977	Missense_Mutation	C	A	P4T2
LGR5	chr12	71977573	Missense_Mutation	T	A	P4T2
DCN	chr12	91546900	Missense_Mutation	C	A	P4T2
TBX3	chr12	115117423	Missense_Mutation	G	C	P4T2
RNFT2	chr12	117187929	Missense_Mutation	G	T	P4T2
CCDC60	chr12	119937969	Missense_Mutation	C	T	P4T2
DHX37	chr12	125470802	Missense_Mutation	G	A	P4T2
TMEM132B	chr12	126139071	Missense_Mutation	G	T	P4T2
PSPC1	chr13	20356650	Missense_Mutation	C	A	P4T2
DIS3	chr13	73342935	Missense_Mutation	C	A	P4T2
SLITRK6	chr13	86369659	Missense_Mutation	A	T	P4T2
FAM155A	chr13	108518122	Missense_Mutation	C	A	P4T2
RASA3	chr13	114789762	Nonsense_Mutation	C	A	P4T2
MDGA2	chr14	47343281	Nonsense_Mutation	C	A	P4T2
ACOT2	chr14	74035993	Missense_Mutation	T	A	P4T2
PROX2	chr14	75330147	Missense_Mutation	G	T	P4T2
NOXRED1	chr14	77873073	Missense_Mutation	G	A	P4T2
OR4N4	chr15	22383297	Missense_Mutation	C	A	P4T2
GOLGA6L2	chr15	23685802	Missense_Mutation	G	T	P4T2
SNRPN	chr15	25223377	Missense_Mutation	G	T	P4T2
SNRPN	chr15	25223379	Missense_Mutation	G	T	P4T2
GABRB3	chr15	26806091	Missense_Mutation	G	C	P4T2
TJP1	chr15	30019095	Missense_Mutation	T	A	P4T2
STARD9	chr15	42980692	Missense_Mutation	A	C	P4T2
NOX5	chr15	69324100	Missense_Mutation	C	A	P4T2
LOXL1	chr15	74220149	Missense_Mutation	C	A	P4T2
EFL1	chr15	82456252	Missense_Mutation	T	C	P4T2
LINS1	chr15	101114273	Missense_Mutation	G	A	P4T2
FAHD1	chr16	1877395	Missense_Mutation	G	T	P4T2
MEFV	chr16	3293485	Missense_Mutation	C	G	P4T2
TMC5	chr16	19488833	Missense_Mutation	G	T	P4T2
LYRM1	chr16	20927013	Missense_Mutation	A	T	P4T2
UQCRC2	chr16	21991969	Missense_Mutation	C	T	P4T2
KIAA0556	chr16	27720024	Splice_Site	A	G	P4T2
XPO6	chr16	28188654	Splice_Site	C	A	P4T2
ITGAM	chr16	31332569	Missense_Mutation	C	T	P4T2
SLC12A3	chr16	56913533	Missense_Mutation	G	T	P4T2
ZNF319	chr16	58031707	Missense_Mutation	T	G	P4T2
TANGO6	chr16	68894134	Missense_Mutation	G	A	P4T2
HYDIN	chr16	70917838	Missense_Mutation	C	T	P4T2
NPIPB15	chr16	74425961	Missense_Mutation	G	A	P4T2

CA5A	chr16	87925495	Missense_Mutation	C	A	P4T2
USP6	chr17	5072243	Missense_Mutation	C	A	P4T2
PHF23	chr17	7139287	Missense_Mutation	T	C	P4T2
TP53	chr17	7578457	Missense_Mutation	C	A	P4T2
RAI1	chr17	17698552	Nonsense_Mutation	G	T	P4T2
MAP2K3	chr17	21206516	Missense_Mutation	C	A	P4T2
TNFAIP1	chr17	26666698	Missense_Mutation	A	T	P4T2
TMEM132E	chr17	32964337	Missense_Mutation	C	A	P4T2
KRTAP4-9	chr17	39261762	Missense_Mutation	G	A	P4T2
KRTAP9-1	chr17	39346554	Missense_Mutation	G	T	P4T2
KRT13	chr17	39661729	Missense_Mutation	C	T	P4T2
STAT5B	chr17	40364147	Missense_Mutation	T	C	P4T2
PLEKHM1	chr17	43552814	Missense_Mutation	G	A	P4T2
AXIN2	chr17	63532631	Missense_Mutation	G	A	P4T2
OTOP2	chr17	72927130	Missense_Mutation	T	C	P4T2
EVPL	chr17	74011709	Missense_Mutation	C	A	P4T2
PSMG2	chr18	12720577	Missense_Mutation	A	T	P4T2
CELF4	chr18	34854362	Missense_Mutation	A	T	P4T2
ALPK2	chr18	56202602	Missense_Mutation	G	A	P4T2
ZNRF4	chr19	5456235	Missense_Mutation	G	C	P4T2
KIAA1683	chr19	18368808	Missense_Mutation	G	A	P4T2
ZNF43	chr19	21992271	Missense_Mutation	C	A	P4T2
CEP89	chr19	33422400	Missense_Mutation	C	G	P4T2
KMT2B	chr19	36223581	Missense_Mutation	C	T	P4T2
RYR1	chr19	38964306	Missense_Mutation	C	A	P4T2
PSG2	chr19	43579749	Missense_Mutation	A	T	P4T2
ZNF229	chr19	44933896	Missense_Mutation	A	T	P4T2
EHD2	chr19	48244289	Missense_Mutation	G	T	P4T2
ETFB	chr19	51857484	Missense_Mutation	C	A	P4T2
ZNF578	chr19	53014752	Missense_Mutation	G	T	P4T2
PRKCG	chr19	54387477	Missense_Mutation	G	T	P4T2
ZNF628	chr19	55993843	Missense_Mutation	G	T	P4T2
ZNF583	chr19	56925764	Missense_Mutation	G	A	P4T2
ZNF8	chr19	58806117	Missense_Mutation	T	A	P4T2
CSTL1	chr20	23420993	Missense_Mutation	G	T	P4T2
SLA2	chr20	35242798	Missense_Mutation	A	G	P4T2
JPH2	chr20	42788307	Missense_Mutation	G	T	P4T2
ZNFX1	chr20	47873967	Missense_Mutation	G	A	P4T2
SLCO4A1	chr20	61299241	Missense_Mutation	G	C	P4T2
OGFR	chr20	61443911	Missense_Mutation	G	T	P4T2
DIDO1	chr20	61525054	Missense_Mutation	G	C	P4T2
DIDO1	chr20	61526166	Nonsense_Mutation	G	C	P4T2
COL20A1	chr20	61944287	Splice_Site	G	T	P4T2
KCNQ2	chr20	62038298	Missense_Mutation	C	A	P4T2

KCNQ2	chr20	62050975	Missense_Mutation	G	T	P4T2
HELZ2	chr20	62195538	Missense_Mutation	G	A	P4T2
HELZ2	chr20	62195576	Missense_Mutation	A	C	P4T2
LINC00176	chr20	62669740	Missense_Mutation	C	T	P4T2
NCAM2	chr21	22658675	Missense_Mutation	C	T	P4T2
KRTAP25-1	chr21	31661771	Missense_Mutation	C	A	P4T2
KRTAP13-1	chr21	31768459	Missense_Mutation	C	A	P4T2
GAB4	chr22	17446146	Missense_Mutation	G	C	P4T2
RFPL2	chr22	32588948	Missense_Mutation	T	C	P4T2
ENTHD1	chr22	40139932	Missense_Mutation	G	T	P4T2
SBF1	chr22	50895521	Missense_Mutation	C	A	P4T2
MXRA5	chrX	3235182	Missense_Mutation	C	A	P4T2
MXRA5	chrX	3235183	Missense_Mutation	C	A	P4T2
CFAP47	chrX	36103528	Missense_Mutation	G	T	P4T2
RRAGB	chrX	55757825	Missense_Mutation	A	C	P4T2
SATL1	chrX	84363524	Missense_Mutation	G	T	P4T2
CHM	chrX	85233861	Missense_Mutation	C	A	P4T2
CPXCR1	chrX	88008694	Missense_Mutation	G	T	P4T2
PCDH11X	chrX	91132681	Missense_Mutation	C	A	P4T2
IGSF1	chrX	130408605	Missense_Mutation	G	T	P4T2
SAGE1	chrX	134989480	Missense_Mutation	G	T	P4T2
TNN	chr1	175086150	Frame_Shift_Del	G	-	P4T2
BECN2	chr1	242122049	Frame_Shift_Del	C	-	P4T2
ZNF107	chr7	64168858	Frame_Shift_Del	G	-	P4T2
AHNAK	chr11	62286598	Frame_Shift_Del	AGGTGATTTA	-	P4T2
CDH24	chr14	23524274	Splice_Site	CTGGGGTGCTCACCGACATT	-	P4T2
EML5	chr14	89178714	Frame_Shift_Del	C	-	P4T2
MUM1	chr19	1360402	Frame_Shift_Del	C	-	P4T2
OR10H1	chr19	15918388	Frame_Shift_Del	C	-	P4T2
COL18A1	chr21	46888298	Frame_Shift_Del	C	-	P4T2
AJAP1	chr1	4772291	Missense_Mutation	C	A	P4T3
CHD5	chr1	6188252	Missense_Mutation	T	C	P4T3
KPNA6	chr1	32635482	Splice_Site	G	T	P4T3
C1orf94	chr1	34666381	Missense_Mutation	C	A	P4T3
ZMYND12	chr1	42915714	Nonsense_Mutation	T	A	P4T3
PLK3	chr1	45269630	Missense_Mutation	G	T	P4T3
LRRC42	chr1	54432017	Nonsense_Mutation	G	T	P4T3
LRRC7	chr1	70225997	Missense_Mutation	T	A	P4T3
FPGT-TNNI3K	chr1	75009635	Missense_Mutation	G	T	P4T3
LHX8	chr1	75622753	Missense_Mutation	A	T	P4T3

IFI44L	chr1	79106744	Missense_Mutation	T	A	P4T3
HFM1	chr1	91841104	Missense_Mutation	C	A	P4T3
CHD1L	chr1	146766159	Missense_Mutation	A	T	P4T3
BCL9	chr1	147092746	Missense_Mutation	C	G	P4T3
MUC1	chr1	155160889	Missense_Mutation	G	A	P4T3
PEAR1	chr1	156876147	Missense_Mutation	C	A	P4T3
SPTA1	chr1	158590011	Missense_Mutation	T	A	P4T3
AIM2	chr1	159038489	Missense_Mutation	C	A	P4T3
PIGC	chr1	172411408	Missense_Mutation	C	G	P4T3
TNN	chr1	175097817	Missense_Mutation	C	A	P4T3
AXDND1	chr1	179347785	Missense_Mutation	C	A	P4T3
ARPC5	chr1	183604734	Nonsense_Mutation	C	A	P4T3
HHIPL2	chr1	222721340	Missense_Mutation	C	T	P4T3
TAF1A	chr1	222743984	Missense_Mutation	C	T	P4T3
SUSD4	chr1	223441900	Missense_Mutation	T	A	P4T3
C1orf101	chr1	244724181	Missense_Mutation	A	T	P4T3
C1orf101	chr1	244724183	Missense_Mutation	G	A	P4T3
OR2L5	chr1	248186093	Missense_Mutation	C	T	P4T3
OR2M3	chr1	248366877	Missense_Mutation	G	T	P4T3
OR2T1	chr1	248569743	Missense_Mutation	C	A	P4T3
OR2T3	chr1	248637275	Nonsense_Mutation	C	A	P4T3
SLC3A1	chr2	44508631	Missense_Mutation	G	T	P4T3
SLC3A1	chr2	44508632	Missense_Mutation	G	T	P4T3
NRXN1	chr2	50779925	Missense_Mutation	C	A	P4T3
REG3A	chr2	79385471	Missense_Mutation	C	T	P4T3
SLC5A7	chr2	108626946	Missense_Mutation	C	A	P4T3
SLC5A7	chr2	108626947	Missense_Mutation	C	A	P4T3
SULT1C4	chr2	108999570	Missense_Mutation	C	A	P4T3
POTEE	chr2	132021527	Nonsense_Mutation	C	A	P4T3
LCT	chr2	136566212	Missense_Mutation	C	A	P4T3
LRP1B	chr2	141474298	Missense_Mutation	G	T	P4T3
GALNT13	chr2	155098580	Missense_Mutation	A	G	P4T3
XIRP2	chr2	168100312	Missense_Mutation	G	T	P4T3
CERKL	chr2	182423392	Missense_Mutation	G	A	P4T3
UNC80	chr2	210704037	Missense_Mutation	C	A	P4T3
CXCR2	chr2	218999545	Missense_Mutation	G	T	P4T3
DOCK10	chr2	225698951	Missense_Mutation	C	A	P4T3
CNTN4	chr3	2944559	Splice_Site	G	T	P4T3
CNTN4	chr3	2944560	Missense_Mutation	G	T	P4T3
ITPR1	chr3	4715032	Missense_Mutation	G	T	P4T3
SYN2	chr3	12232029	Missense_Mutation	G	T	P4T3
LAMB2	chr3	49161498	Nonsense_Mutation	G	A	P4T3
CADPS	chr3	62535687	Missense_Mutation	C	A	P4T3
ZPLD1	chr3	102189341	Missense_Mutation	G	A	P4T3

KALRN	chr3	124180735	Splice_Site	A	G	P4T3
TF	chr3	133472444	Missense_Mutation	C	G	P4T3
ADIPOQ	chr3	186571011	Missense_Mutation	G	T	P4T3
NFXL1	chr4	47898646	Missense_Mutation	G	A	P4T3
FRAS1	chr4	79343056	Missense_Mutation	G	T	P4T3
NDST4	chr4	115760613	Missense_Mutation	G	A	P4T3
KIAA1109	chr4	123280853	Missense_Mutation	C	T	P4T3
SMARCA5	chr4	144445576	Missense_Mutation	C	G	P4T3
NEIL3	chr4	178243698	Missense_Mutation	A	T	P4T3
TENM3	chr4	183714140	Missense_Mutation	G	T	P4T3
CDH6	chr5	31323050	Missense_Mutation	C	G	P4T3
ADAMTS12	chr5	33576549	Missense_Mutation	C	A	P4T3
RANBP3L	chr5	36262041	Missense_Mutation	C	A	P4T3
C7	chr5	40962256	Missense_Mutation	G	C	P4T3
EDIL3	chr5	83549905	Missense_Mutation	C	A	P4T3
ARHGAP26	chr5	142602010	Nonstop_Mutation	A	C	P4T3
FAM71B	chr5	156590536	Missense_Mutation	G	T	P4T3
FABP6	chr5	159640818	Missense_Mutation	A	G	P4T3
HMMR	chr5	162911152	Missense_Mutation	A	T	P4T3
MGAT1	chr5	180219785	Missense_Mutation	C	A	P4T3
DEK	chr6	18258194	Missense_Mutation	C	A	P4T3
ITPR3	chr6	33652600	Missense_Mutation	C	G	P4T3
CDC5L	chr6	44358038	Missense_Mutation	G	T	P4T3
MYO6	chr6	76599775	Missense_Mutation	C	T	P4T3
FIG4	chr6	110083343	Missense_Mutation	G	T	P4T3
L3MBTL3	chr6	130425611	Missense_Mutation	G	T	P4T3
MOXD1	chr6	132649569	Missense_Mutation	C	A	P4T3
VNN2	chr6	133077157	Missense_Mutation	A	T	P4T3
UTRN	chr6	144832156	Missense_Mutation	T	C	P4T3
FAM188B	chr7	30811111	Translation_Start_Site	T	C	P4T3
ELMO1	chr7	36927207	Missense_Mutation	C	T	P4T3
ABCA13	chr7	48315346	Missense_Mutation	T	C	P4T3
ZNF735	chr7	63680486	Missense_Mutation	T	C	P4T3
ABCB4	chr7	87053326	Missense_Mutation	G	T	P4T3
STEAP2	chr7	89856613	Missense_Mutation	G	T	P4T3
KRIT1	chr7	91864785	Missense_Mutation	C	A	P4T3
PPP1R9A	chr7	94791231	Missense_Mutation	G	T	P4T3
DYNC1I1	chr7	95662014	Missense_Mutation	G	T	P4T3
SH2B2	chr7	101960913	Missense_Mutation	G	T	P4T3
LAMB1	chr7	107626714	Missense_Mutation	G	A	P4T3
GCC1	chr7	127224536	Missense_Mutation	T	C	P4T3
METTL2B	chr7	128141894	Missense_Mutation	G	A	P4T3
CPA4	chr7	129948201	Missense_Mutation	G	T	P4T3
NUP205	chr7	135282924	Missense_Mutation	G	T	P4T3

DGKI	chr7	137170139	Missense_Mutation	G	A	P4T3
KEL	chr7	142639564	Missense_Mutation	A	T	P4T3
ZNF862	chr7	149545238	Missense_Mutation	G	A	P4T3
CCAR2	chr8	22473403	Missense_Mutation	C	T	P4T3
PXDNL	chr8	52321566	Missense_Mutation	C	A	P4T3
RIMS2	chr8	105080823	Missense_Mutation	C	A	P4T3
PKHD1L1	chr8	110456903	Missense_Mutation	G	T	P4T3
CSMD3	chr8	113812465	Missense_Mutation	A	C	P4T3
ENPP2	chr8	120633750	Missense_Mutation	C	A	P4T3
DENND4C	chr9	19360308	Missense_Mutation	C	T	P4T3
IFNA2	chr9	21385139	Nonsense_Mutation	C	A	P4T3
TAF1L	chr9	32631934	Missense_Mutation	C	A	P4T3
PCSK5	chr9	78771967	Missense_Mutation	A	T	P4T3
SPATA31D1	chr9	84608091	Missense_Mutation	G	T	P4T3
NTRK2	chr9	87563441	Missense_Mutation	A	T	P4T3
KLF4	chr9	110250178	Missense_Mutation	C	A	P4T3
DDX31	chr9	135534080	Missense_Mutation	T	A	P4T3
ZNF37A	chr10	38406617	Missense_Mutation	G	T	P4T3
WDFY4	chr10	49929315	Missense_Mutation	G	T	P4T3
ADAMTS14	chr10	72492112	Missense_Mutation	A	G	P4T3
SORCS3	chr10	106970978	Missense_Mutation	G	T	P4T3
PRLHR	chr10	120354419	Missense_Mutation	C	A	P4T3
FAM196A	chr10	128973783	Missense_Mutation	G	T	P4T3
AP2A2	chr11	977133	Missense_Mutation	G	T	P4T3
OR51F2	chr11	4842724	Missense_Mutation	A	T	P4T3
OR52J3	chr11	5068004	Missense_Mutation	G	T	P4T3
SMPD1	chr11	6412727	Missense_Mutation	G	T	P4T3
OTOG	chr11	17578870	Missense_Mutation	G	T	P4T3
OTOG	chr11	17653686	Missense_Mutation	C	A	P4T3
DEPDC7	chr11	33054903	Missense_Mutation	G	T	P4T3
LRRC4C	chr11	40136066	Nonsense_Mutation	C	A	P4T3
OR4C3	chr11	48347133	Missense_Mutation	G	A	P4T3
CD5	chr11	60887023	Nonsense_Mutation	C	T	P4T3
ATG2A	chr11	64671082	Missense_Mutation	T	A	P4T3
KRTAP5-10	chr11	71276733	Missense_Mutation	G	C	P4T3
MRPL48	chr11	73536783	Missense_Mutation	A	G	P4T3
SLCO2B1	chr11	74880761	Missense_Mutation	G	T	P4T3
TMEM135	chr11	87006823	Splice_Site	G	A	P4T3
MMP10	chr11	102651278	Nonsense_Mutation	G	T	P4T3
APOA5	chr11	116661325	Missense_Mutation	T	C	P4T3
ESAM	chr11	124624174	Missense_Mutation	C	G	P4T3
CLEC7A	chr12	10275865	Missense_Mutation	C	A	P4T3
TAS2R46	chr12	11214365	Missense_Mutation	T	C	P4T3
ST8SIA1	chr12	22440145	Missense_Mutation	A	G	P4T3

KRAS	chr12	25398285	Missense_Mutation	C	A	P4T3
GALNT6	chr12	51773410	Missense_Mutation	G	T	P4T3
KRT5	chr12	52913941	Missense_Mutation	C	A	P4T3
HOXC11	chr12	54367185	Missense_Mutation	G	T	P4T3
OR9K2	chr12	55524064	Missense_Mutation	C	A	P4T3
TMTC2	chr12	83359383	Missense_Mutation	A	G	P4T3
RASSF9	chr12	86199548	Missense_Mutation	T	C	P4T3
TMTC3	chr12	88553954	Missense_Mutation	G	T	P4T3
TMCC3	chr12	94965256	Missense_Mutation	C	A	P4T3
CMKLR1	chr12	108686439	Missense_Mutation	T	A	P4T3
NAA25	chr12	112530860	Missense_Mutation	C	G	P4T3
OAS3	chr12	113382289	Missense_Mutation	G	T	P4T3
SDSL	chr12	113865840	Missense_Mutation	C	T	P4T3
SPPL3	chr12	121202870	Missense_Mutation	C	G	P4T3
AACS	chr12	125599062	Missense_Mutation	G	T	P4T3
TMEM132C	chr12	129190521	Missense_Mutation	G	T	P4T3
TPTE2	chr13	20012267	Missense_Mutation	G	T	P4T3
RNF17	chr13	25419184	Missense_Mutation	G	T	P4T3
FRY	chr13	32783741	Missense_Mutation	G	T	P4T3
CCDC168	chr13	103391720	Missense_Mutation	C	A	P4T3
CCDC168	chr13	103395288	Missense_Mutation	C	A	P4T3
OR4M1	chr14	20248993	Missense_Mutation	C	A	P4T3
OR4N2	chr14	20296167	Missense_Mutation	T	A	P4T3
OR4K15	chr14	20444006	Missense_Mutation	T	A	P4T3
ESRRB	chr14	76964644	Missense_Mutation	G	A	P4T3
NRXN3	chr14	79111729	Missense_Mutation	T	A	P4T3
AHNAK2	chr14	105408035	Missense_Mutation	G	A	P4T3
AHNAK2	chr14	105411161	Nonsense_Mutation	C	A	P4T3
TMEM121	chr14	105995953	Missense_Mutation	C	T	P4T3
NDN	chr15	23932066	Missense_Mutation	T	C	P4T3
NDN	chr15	23932067	Nonsense_Mutation	G	A	P4T3
RYR3	chr15	33895371	Missense_Mutation	A	G	P4T3
LTK	chr15	41797646	Missense_Mutation	A	T	P4T3
MAP1A	chr15	43814169	Missense_Mutation	A	C	P4T3
CSNK1G1	chr15	64506305	Missense_Mutation	C	A	P4T3
TBC1D2B	chr15	78305267	Missense_Mutation	T	C	P4T3
ACSBG1	chr15	78466762	Nonsense_Mutation	G	A	P4T3
NTRK3	chr15	88472547	Missense_Mutation	G	T	P4T3
TRAF7	chr16	2223556	Splice_Site	G	T	P4T3
GTF3C1	chr16	27549533	Missense_Mutation	C	A	P4T3
CD19	chr16	28948376	Missense_Mutation	G	T	P4T3
IST1	chr16	71956431	Missense_Mutation	G	T	P4T3
CMC2	chr16	81015413	Nonsense_Mutation	C	A	P4T3
PLCG2	chr16	81946282	Missense_Mutation	G	A	P4T3

GSE1	chr16	85688462	Missense_Mutation	G	T	P4T3
USP43	chr17	9590134	Missense_Mutation	A	T	P4T3
MYH8	chr17	10323527	Missense_Mutation	G	T	P4T3
MYH1	chr17	10401209	Missense_Mutation	C	A	P4T3
KCNJ12	chr17	21319333	Missense_Mutation	C	A	P4T3
KCNJ12	chr17	21319334	Missense_Mutation	A	T	P4T3
KRT12	chr17	39023417	Missense_Mutation	T	A	P4T3
ITGA2B	chr17	42454447	Missense_Mutation	C	T	P4T3
CACNA1G	chr17	48653687	Missense_Mutation	G	T	P4T3
POTEC	chr18	14542773	Missense_Mutation	C	G	P4T3
ASXL3	chr18	31322873	Missense_Mutation	C	A	P4T3
ASXL3	chr18	31324712	Missense_Mutation	C	G	P4T3
FHOD3	chr18	34232688	Missense_Mutation	C	A	P4T3
ST8SIA3	chr18	55024577	Missense_Mutation	C	A	P4T3
TMIGD2	chr19	4292714	Missense_Mutation	G	A	P4T3
ARHGEF18	chr19	7437772	Missense_Mutation	C	G	P4T3
CD209	chr19	7808085	Missense_Mutation	C	A	P4T3
ACTL9	chr19	8808476	Missense_Mutation	G	T	P4T3
MUC16	chr19	8976361	Missense_Mutation	C	A	P4T3
ICAM4	chr19	10398349	Nonsense_Mutation	G	T	P4T3
USHBP1	chr19	17373654	Missense_Mutation	C	A	P4T3
SLC5A5	chr19	17985011	Missense_Mutation	C	T	P4T3
ZNF98	chr19	22574572	Missense_Mutation	G	C	P4T3
ZNF98	chr19	22605005	Missense_Mutation	G	T	P4T3
ZNF98	chr19	22605006	Missense_Mutation	G	T	P4T3
ZNF99	chr19	22941116	Missense_Mutation	G	T	P4T3
ZNF99	chr19	22941783	Nonsense_Mutation	C	A	P4T3
ZNF536	chr19	30935863	Missense_Mutation	C	A	P4T3
ATP4A	chr19	36051485	Missense_Mutation	G	T	P4T3
TMEM145	chr19	42827865	Missense_Mutation	A	G	P4T3
PLAUR	chr19	44174266	Missense_Mutation	G	A	P4T3
EMC10	chr19	50983395	Missense_Mutation	C	T	P4T3
ZNF415	chr19	53612487	Missense_Mutation	G	T	P4T3
ZNF765-ZNF761	chr19	53959509	Missense_Mutation	G	A	P4T3
CACNG6	chr19	54496175	Missense_Mutation	G	A	P4T3
PEG3	chr19	57326799	Missense_Mutation	C	A	P4T3
USP29	chr19	57641166	Missense_Mutation	G	C	P4T3
TMEM239	chr20	2797252	Missense_Mutation	C	A	P4T3
PROKR2	chr20	5283101	Missense_Mutation	G	A	P4T3
SEC23B	chr20	18505625	Missense_Mutation	G	C	P4T3
FOXS1	chr20	30432567	Missense_Mutation	C	A	P4T3
CDK5RAP1	chr20	31975172	Missense_Mutation	C	T	P4T3
CHD6	chr20	40040869	Missense_Mutation	C	A	P4T3

KRTAP10-11	chr21	46067016	Missense_Mutation	C	T	P4T3
MYO18B	chr22	26159316	Missense_Mutation	G	A	P4T3
SLC5A1	chr22	32480517	Missense_Mutation	G	T	P4T3
TTLL12	chr22	43570321	Missense_Mutation	C	A	P4T3
PNPLA3	chr22	44332993	Missense_Mutation	G	T	P4T3
MAGEB3	chrX	30254346	Missense_Mutation	C	A	P4T3
FAM47C	chrX	37028540	Missense_Mutation	G	T	P4T3
RBM10	chrX	47044943	Nonsense_Mutation	C	T	P4T3
GPRASP1	chrX	101910289	Missense_Mutation	C	A	P4T3
FGF13	chrX	137939830	Missense_Mutation	C	G	P4T3
AFF2	chrX	147743521	Missense_Mutation	G	T	P4T3
TNFRSF4	chr1	1147103	Frame_Shift_Del	G	-	P4T3
ZNF852	chr3	44541660	Frame_Shift_Del	GAGATGAACA	-	P4T3
PRR23A	chr3	138724660	In_Frame_Del	GGC	-	P4T3
TRMT44	chr4	8443131	Frame_Shift_Del	C	-	P4T3
SLC12A2	chr5	127484552	In_Frame_Ins	-	TTGGATTCATCTTAATTGGTT	P4T3
MYH6	chr14	23874013	Frame_Shift_Del	C	-	P4T3
DNAH3	chr16	20986573	Frame_Shift_Del	G	-	P4T3
TNFAIP1	chr17	26666554	Translation_Start_Site	GGGAGATGTCGG	-	P4T3
UPK3A	chr22	45691548	Frame_Shift_Del	G	-	P4T3
EPHA8	chr1	22928223	Missense_Mutation	C	T	P5T1
KIF4B	chr5	154395032	Missense_Mutation	C	T	P5T1
MDN1	chr6	90359745	Missense_Mutation	G	C	P5T1
KLRG2	chr7	139164491	Missense_Mutation	C	G	P5T1
PDZD8	chr10	119044370	Missense_Mutation	G	A	P5T1
DDB2	chr11	47236707	Missense_Mutation	C	T	P5T1
PABPC3	chr13	25671451	Missense_Mutation	A	G	P5T1
PRSS22	chr16	2905614	Missense_Mutation	G	T	P5T1
PSMA7	chr20	60713282	Missense_Mutation	T	G	P5T1
NIPSNAP1	chr22	29957575	Missense_Mutation	G	A	P5T1
UBR4	chr1	19482781	Frame_Shift_Del	GTCTGCGG	-	P5T1
BRD3	chr9	136916781	Frame_Shift_Ins	-	T	P5T1
MEGF6	chr1	3411225	Missense_Mutation	C	T	P5T2
TMEM82	chr1	16070934	Missense_Mutation	C	T	P5T2
HNRNPR	chr1	23660009	Splice_Site	A	C	P5T2
SPTBN1	chr2	54874343	Missense_Mutation	G	A	P5T2
DNAH7	chr2	196726616	Missense_Mutation	C	G	P5T2
CTLA4	chr2	204735626	Missense_Mutation	A	G	P5T2
ILKAP	chr2	239092317	Missense_Mutation	T	C	P5T2
GFM2	chr5	74034402	Missense_Mutation	G	C	P5T2

SLCO4C1	chr5	101627163	Missense_Mutation	C	G	P5T2
JAKMIP2	chr5	147051318	Missense_Mutation	C	A	P5T2
CAGE1	chr6	7387334	Missense_Mutation	C	T	P5T2
NHLRC1	chr6	18122746	Missense_Mutation	T	G	P5T2
PLEC	chr8	144999785	Missense_Mutation	G	A	P5T2
ENTPD7	chr10	101458483	Missense_Mutation	T	G	P5T2
OR52L1	chr11	6007208	Missense_Mutation	C	T	P5T2
C2CD2L	chr11	118981347	Missense_Mutation	C	T	P5T2
KCNJ1	chr11	128709896	Nonsense_Mutation	A	C	P5T2
ISCA2	chr14	74961588	Missense_Mutation	A	C	P5T2
RASGRF1	chr15	79296243	Missense_Mutation	T	G	P5T2
TSC2	chr16	2130184	Missense_Mutation	T	G	P5T2
ACSM2A	chr16	20489974	Missense_Mutation	C	G	P5T2
KDM8	chr16	27221856	Missense_Mutation	C	G	P5T2
ATAD5	chr17	29161766	Missense_Mutation	G	A	P5T2
KAT7	chr17	47874288	Missense_Mutation	G	A	P5T2
ST8SIA5	chr18	44260450	Missense_Mutation	C	T	P5T2
SLC1A6	chr19	15061033	Missense_Mutation	G	A	P5T2
ZNF570	chr19	37975405	Missense_Mutation	G	A	P5T2
MEGF8	chr19	42839202	Missense_Mutation	C	T	P5T2
ASXL1	chr20	31024134	Missense_Mutation	G	T	P5T2
ATF4	chr22	39918080	Missense_Mutation	T	C	P5T2
AKAP4	chrX	49958667	Missense_Mutation	G	T	P5T2
FAT2	chr5	150925485	Frame_Shift_Del	ATGACATC AGTAAATG CACCTGCC AT	-	P5T2
HNRNPAB	chr5	177632880	Frame_Shift_Del	A	-	P5T2
EGFR	chr7	55242479	In_Frame_Del	GGAATTAA GAGAAGC	-	P5T2
TSHZ2	chr20	51872279	In_Frame_Del	CAG	-	P5T2
SLC7A8	chr14	23612372	Missense_Mutation	T	G	P6N
MAP1A	chr15	43814169	Missense_Mutation	A	C	P6N
MAP1A	chr15	43814170	Missense_Mutation	A	C	P6N
GRHL3	chr1	24669505	Missense_Mutation	G	A	P6T1
NFASC	chr1	204978787	Missense_Mutation	T	C	P6T1
PRKD3	chr2	37480492	Missense_Mutation	T	C	P6T1
TMEM131	chr2	98421615	Missense_Mutation	C	A	P6T1
FN1	chr2	216286817	Nonsense_Mutation	G	A	P6T1
DHX36	chr3	154007595	Missense_Mutation	T	C	P6T1
NLGN1	chr3	173999073	Missense_Mutation	C	T	P6T1
ANKRD17	chr4	74013032	Missense_Mutation	A	C	P6T1
DCTD	chr4	183836685	Missense_Mutation	A	C	P6T1
DEPDC1B	chr5	59899376	Missense_Mutation	G	A	P6T1

PCDHGA1	chr5	140711459	Missense_Mutation	G	A	P6T1
JAKMIP2	chr5	147051353	Missense_Mutation	C	T	P6T1
DNAH11	chr7	21779287	Missense_Mutation	T	C	P6T1
EGFR	chr7	55241708	Missense_Mutation	G	C	P6T1
EGFR	chr7	55249029	Missense_Mutation	G	A	P6T1
CALU	chr7	128394437	Missense_Mutation	G	A	P6T1
EXT1	chr8	118831998	Missense_Mutation	G	A	P6T1
ZNF510	chr9	99521309	Missense_Mutation	T	G	P6T1
SEC16A	chr9	139353675	Missense_Mutation	A	T	P6T1
DIP2C	chr10	329226	Missense_Mutation	G	C	P6T1
OPTN	chr10	13152294	Nonsense_Mutation	C	T	P6T1
TRIM8	chr10	104404586	Missense_Mutation	T	G	P6T1
CPXM2	chr10	125526622	Missense_Mutation	C	T	P6T1
KNDC1	chr10	135033473	Missense_Mutation	C	G	P6T1
SLC43A3	chr11	57176757	Missense_Mutation	T	G	P6T1
OR4D6	chr11	59225187	Missense_Mutation	G	A	P6T1
OVOL1	chr11	65554852	Missense_Mutation	G	T	P6T1
OR10G9	chr11	123894410	Nonsense_Mutation	G	T	P6T1
KRT7	chr12	52631309	Missense_Mutation	A	C	P6T1
SPRYD3	chr12	53460395	Missense_Mutation	C	A	P6T1
MYF5	chr12	81112140	Missense_Mutation	G	A	P6T1
CARMIL3	chr14	24530777	Missense_Mutation	C	A	P6T1
KLHL28	chr14	45414740	Missense_Mutation	T	C	P6T1
DLK1	chr14	101198381	Missense_Mutation	G	T	P6T1
AHNAK2	chr14	105419224	Nonsense_Mutation	G	T	P6T1
GOLGA6L1	chr15	22742670	Missense_Mutation	G	A	P6T1
IGFALS	chr16	1841982	Missense_Mutation	G	A	P6T1
GRIN2A	chr16	9858166	Missense_Mutation	T	A	P6T1
LRRC37B	chr17	30349799	Missense_Mutation	T	A	P6T1
TMEM132E	chr17	32964762	Missense_Mutation	T	G	P6T1
UNK	chr17	73816030	Missense_Mutation	G	A	P6T1
CACNA1A	chr19	13563723	Nonsense_Mutation	C	T	P6T1
BRSK1	chr19	55800916	Missense_Mutation	C	T	P6T1
U2SURP	chr3	142741833	In_Frame_Del	GCCTTTTA ATGCGCA	-	P6T1
UNC5C	chr4	96091465	Frame_Shift_Del	AA	-	P6T1
EGFR	chr7	55242479	In_Frame_Del	GGAATTAA GAGAAGC	-	P6T1
DMTF1	chr7	86824122	Frame_Shift_Del	AATCTGTC	-	P6T1
RUSC2	chr9	35546788	Frame_Shift_Del	GGAT	-	P6T1
KCNJ11	chr11	17408902	Frame_Shift_Del	C	-	P6T1
DDX11	chr12	31256581	Frame_Shift_Del	T	-	P6T1

KRT4	chr12	53207584	In_Frame_Ins	-	CACCAAAGCCACCAGTGCCGAAACCAGCTCCGAAGCCGCCGG	P6T1
VPS13D	chr1	12418630	Missense_Mutation	A	C	P6T2
MFHAS1	chr8	8749032	Missense_Mutation	G	A	P6T2
ODF1	chr8	103572804	Missense_Mutation	T	G	P6T2
ODF1	chr8	103572806	Missense_Mutation	T	G	P6T2
KRTAP5-5	chr11	1651615	Missense_Mutation	A	G	P6T2
GOLGA6L1	chr15	22742670	Missense_Mutation	G	A	P6T2
KRT4	chr12	53207584	In_Frame_Ins	-	CACCAAAGCCACCAGTGCCGAAACCAGCTCCGAAGCCGCCGG	P6T2
DISP1	chr1	223116396	Missense_Mutation	G	A	P6T3
MOGS	chr2	74689021	Missense_Mutation	G	A	P6T3
CEP120	chr5	122734970	Missense_Mutation	T	C	P6T3
KIF4B	chr5	154395374	Missense_Mutation	G	A	P6T3
ADAM19	chr5	156991423	Missense_Mutation	A	G	P6T3
EGFR	chr7	55259515	Missense_Mutation	T	G	P6T3
PIGO	chr9	35091449	Nonsense_Mutation	A	C	P6T3
HMCN2	chr9	133241057	Missense_Mutation	T	C	P6T3
NOP2	chr12	6670182	Missense_Mutation	C	T	P6T3
IRAK4	chr12	44180286	Missense_Mutation	T	A	P6T3
DACH1	chr13	72063203	Missense_Mutation	G	T	P6T3
TRPM4	chr19	49693520	Missense_Mutation	C	T	P6T3
SEC24B	chr4	110394186	Frame_Shift_Ins	-	A	P6T3
PPARGC1B	chr5	149225441	In_Frame_Del	TCCAGCTGAGCTACGGAGGGC	-	P6T3
KRT4	chr12	53207584	In_Frame_Ins	-	CACCAAAGCCACCAGTGCCGAAACCAGCTCCGAAGCCGCCGG	P6T3
NAV3	chr12	78400984	Frame_Shift_Del	GA	-	P6T3
CACNA1E	chr1	181706753	Missense_Mutation	C	T	P7N
STK36	chr2	219562686	Missense_Mutation	G	A	P7N
PCDHA6	chr5	140207965	Missense_Mutation	G	C	P7N
MAP1A	chr15	43814169	Missense_Mutation	A	C	P7N
EIF3J	chr15	44829415	Missense_Mutation	C	G	P7N
SPANXN2	chrX	142795238	Missense_Mutation	G	T	P7N
FRMD4A	chr10	13696452	Frame_Shift_Del	G	-	P7N
DISP3	chr1	11589728	Missense_Mutation	G	A	P7T1
USH2A	chr1	215901653	Missense_Mutation	C	T	P7T1
ATAD2B	chr2	23988489	Missense_Mutation	A	T	P7T1
RGPD2	chr2	88125215	Missense_Mutation	G	C	P7T1
BRD9	chr5	865541	Nonsense_Mutation	G	A	P7T1

BRD9	chr5	865601	Missense_Mutation	G	A	P7T1
NHLRC1	chr6	18121883	Missense_Mutation	T	G	P7T1
OR2B2	chr6	27879586	Missense_Mutation	T	A	P7T1
TRERF1	chr6	42231123	Missense_Mutation	C	T	P7T1
TDRD6	chr6	46656793	Missense_Mutation	C	T	P7T1
CHST12	chr7	2472653	Missense_Mutation	G	A	P7T1
PTPRZ1	chr7	121651489	Missense_Mutation	A	T	P7T1
LRP12	chr8	105509211	Missense_Mutation	C	A	P7T1
PTPRD	chr9	8501006	Missense_Mutation	T	C	P7T1
BMI1	chr10	22616894	Missense_Mutation	A	G	P7T1
KIAA1462	chr10	30315859	Missense_Mutation	G	T	P7T1
LDLRAD3	chr11	36248794	Missense_Mutation	C	T	P7T1
SAP18	chr13	21715110	Missense_Mutation	C	A	P7T1
RPL23	chr17	37009959	Missense_Mutation	G	A	P7T1
KRT37	chr17	39578578	Missense_Mutation	C	T	P7T1
TEX13A	chrX	104465015	Missense_Mutation	C	T	P7T1
PRRG3	chrX	150869164	Missense_Mutation	C	T	P7T1
TMLHE	chrX	154743681	Missense_Mutation	T	C	P7T1
EGFR	chr7	55242487	In_Frame_Del	TAAGAGAAGCAACATCTC	-	P7T1
KIF24	chr9	34255089	In_Frame_Del	TCA	-	P7T1
SIN3A	chr15	75715130	In_Frame_Del	TAGCGGGCC	-	P7T1
SOX15	chr17	7492872	Frame_Shift_Ins	-	G	P7T1
NBPF1	chr1	16902931	Missense_Mutation	C	G	P7T2
TMEM175	chr4	951653	Missense_Mutation	T	G	P7T2
EGFR	chr7	55259515	Missense_Mutation	T	G	P7T2
ANKRD62	chr18	12097661	Missense_Mutation	C	T	P7T2
FAM46D	chrX	79698872	Missense_Mutation	G	C	P7T2
DGKZ	chr11	46369300	Frame_Shift_Ins	-	C	P7T2
FLG	chr1	152279743	Missense_Mutation	C	T	P8N
PXDN	chr2	1677511	Nonsense_Mutation	G	A	P8N
DVL1	chr1	1273941	Missense_Mutation	G	A	P8T1
ARHGEF10L	chr1	17983081	Missense_Mutation	C	T	P8T1
SLC6A17	chr1	110714774	Missense_Mutation	G	A	P8T1
KCND3	chr1	112319765	Missense_Mutation	C	T	P8T1
FLG	chr1	152279743	Missense_Mutation	C	T	P8T1
DUSP27	chr1	167096793	Missense_Mutation	G	A	P8T1
PAPPA2	chr1	176526051	Missense_Mutation	G	A	P8T1
LRRTM4	chr2	77746684	Missense_Mutation	G	T	P8T1
AAMP	chr2	219131261	Missense_Mutation	T	C	P8T1
ALPI	chr2	233320967	Missense_Mutation	G	A	P8T1

OR6B2	chr2	240969090	Missense_Mutation	T	C	P8T1
ING5	chr2	242641508	Missense_Mutation	T	C	P8T1
ZBTB20	chr3	114070359	Missense_Mutation	C	T	P8T1
MXD4	chr4	2252348	Missense_Mutation	T	A	P8T1
MSX1	chr4	4864523	Missense_Mutation	C	T	P8T1
ADCYAP1R1	chr7	31124969	Missense_Mutation	T	C	P8T1
EGFR	chr7	55259515	Missense_Mutation	T	G	P8T1
MGAM	chr7	141759384	Missense_Mutation	G	A	P8T1
PTPRN2	chr7	157341689	Missense_Mutation	G	T	P8T1
IMPAD1	chr8	57905865	Missense_Mutation	A	C	P8T1
RASEF	chr9	85615921	Missense_Mutation	A	C	P8T1
KLF4	chr9	110249807	Missense_Mutation	C	A	P8T1
PDHX	chr11	34988189	Missense_Mutation	A	G	P8T1
TRPC6	chr11	101353884	Missense_Mutation	T	C	P8T1
MMP17	chr12	132335488	Missense_Mutation	G	A	P8T1
BRCA2	chr13	32914471	Missense_Mutation	A	C	P8T1
FARP1	chr13	99037079	Missense_Mutation	G	A	P8T1
OR4Q3	chr14	20216266	Missense_Mutation	G	T	P8T1
FMN1	chr15	33445323	Missense_Mutation	A	T	P8T1
KRTAP16-1	chr17	39464205	Missense_Mutation	C	T	P8T1
RTTN	chr18	67684705	Missense_Mutation	G	C	P8T1
SHROOM4	chrX	50381211	Missense_Mutation	C	T	P8T1
TMEM255A	chrX	119410847	Missense_Mutation	C	A	P8T1
SLC34A2	chr4	25676003	Frame_Shift_Ins	-	T	P8T1
ARHGEF10L	chr1	17983081	Missense_Mutation	C	T	P8T2
LRRC7	chr1	70505424	Missense_Mutation	C	A	P8T2
SLC6A17	chr1	110714774	Missense_Mutation	G	A	P8T2
KCND3	chr1	112319765	Missense_Mutation	C	T	P8T2
EHBP1	chr2	63091974	Missense_Mutation	A	T	P8T2
AAMP	chr2	219131261	Missense_Mutation	T	C	P8T2
ALPI	chr2	233320967	Missense_Mutation	G	A	P8T2
OR6B2	chr2	240969090	Missense_Mutation	T	C	P8T2
ING5	chr2	242641508	Missense_Mutation	T	C	P8T2
ZNF654	chr3	88190016	Missense_Mutation	G	A	P8T2
ZBTB20	chr3	114070359	Missense_Mutation	C	T	P8T2
MYLK	chr3	123457805	Missense_Mutation	T	C	P8T2
PRKCI	chr3	169985778	Missense_Mutation	G	A	P8T2
CD38	chr4	15841738	Missense_Mutation	C	T	P8T2
APC	chr5	112151261	Nonsense_Mutation	C	T	P8T2
PCDHB15	chr5	140625471	Missense_Mutation	T	A	P8T2
JARID2	chr6	15374381	Missense_Mutation	C	T	P8T2
SLC18B1	chr6	133094136	Missense_Mutation	C	T	P8T2

INTS1	chr7	1539980	Missense_Mutation	A	C	P8T2
ADCYAP1R1	chr7	31124969	Missense_Mutation	T	C	P8T2
EGFR	chr7	55259515	Missense_Mutation	T	G	P8T2
MUC12	chr7	100634874	Missense_Mutation	C	A	P8T2
MGAM	chr7	141759384	Missense_Mutation	G	A	P8T2
C7orf33	chr7	148311216	Nonsense_Mutation	G	A	P8T2
HR	chr8	21973938	Nonsense_Mutation	G	A	P8T2
BMP1	chr8	22022968	Missense_Mutation	C	A	P8T2
IMPAD1	chr8	57905865	Missense_Mutation	A	C	P8T2
PRUNE2	chr9	79320991	Missense_Mutation	C	T	P8T2
KLF4	chr9	110249807	Missense_Mutation	C	A	P8T2
HMCN2	chr9	133223943	Missense_Mutation	A	C	P8T2
FZD8	chr10	35928655	Missense_Mutation	G	A	P8T2
MRPS16	chr10	75011698	Missense_Mutation	G	A	P8T2
GLUD1	chr10	88819031	Splice_Site	C	T	P8T2
PNLIPRP1	chr10	118354344	Missense_Mutation	G	A	P8T2
PDHX	chr11	34988189	Missense_Mutation	A	G	P8T2
TRPC6	chr11	101353884	Missense_Mutation	T	C	P8T2
DYNC2H1	chr11	103025313	Missense_Mutation	A	T	P8T2
SIK2	chr11	111590676	Missense_Mutation	C	T	P8T2
OTOGL	chr12	80732904	Missense_Mutation	T	A	P8T2
MMP17	chr12	132335488	Missense_Mutation	G	A	P8T2
BRCA2	chr13	32914471	Missense_Mutation	A	C	P8T2
FARP1	chr13	99037079	Missense_Mutation	G	A	P8T2
NEK9	chr14	75593520	Missense_Mutation	A	C	P8T2
FMN1	chr15	33445323	Missense_Mutation	A	T	P8T2
ARHGDIG	chr16	331914	Missense_Mutation	C	A	P8T2
KRTAP1-1	chr17	39197618	Missense_Mutation	A	T	P8T2
KRTAP16-1	chr17	39464205	Missense_Mutation	C	T	P8T2
BECN1	chr17	40970590	Missense_Mutation	C	T	P8T2
RTTN	chr18	67684705	Missense_Mutation	G	C	P8T2
ZNF556	chr19	2877513	Missense_Mutation	G	A	P8T2
ACVR2A	chr2	148683676	In_Frame_Del	GTT	-	P8T2
GOLGA4	chr3	37368314	Frame_Shift_Del	A	-	P8T2
SLC34A2	chr4	25676003	Frame_Shift_Ins	-	T	P8T2
DBF4	chr7	87536968	In_Frame_Del	CAACCAAAA	-	P8T2
CHD9	chr16	53338409	In_Frame_Ins	-	GTT	P8T2
PTAFR	chr1	28476685	Missense_Mutation	C	A	P8T3
RCC1	chr1	28863399	Missense_Mutation	G	A	P8T3
MYCBP	chr1	39338732	Missense_Mutation	T	C	P8T3
LPGAT1	chr1	211952379	Missense_Mutation	T	A	P8T3
OR2B11	chr1	247614737	Missense_Mutation	C	G	P8T3

INPP4B	chr4	143094892	Missense_Mutation	T	A	P8T3
DCHS2	chr4	155241658	Missense_Mutation	A	C	P8T3
FAM193B	chr5	176959508	Missense_Mutation	G	A	P8T3
PKHD1	chr6	51732740	Missense_Mutation	C	A	P8T3
C10orf82	chr10	118423470	Missense_Mutation	T	C	P8T3
ACP2	chr11	47267104	Missense_Mutation	C	A	P8T3
CBL	chr11	119144699	Missense_Mutation	G	T	P8T3
DNAH10	chr12	124330679	Missense_Mutation	G	A	P8T3
STOML3	chr13	39550920	Missense_Mutation	G	A	P8T3
DCT	chr13	95095702	Missense_Mutation	T	A	P8T3
PCNX4	chr14	60591495	Missense_Mutation	C	T	P8T3
WDR20	chr14	102606450	Missense_Mutation	C	G	P8T3
RYR3	chr15	33999207	Missense_Mutation	G	A	P8T3
ZNF609	chr15	64970525	Missense_Mutation	G	C	P8T3
ST8SIA2	chr15	92987984	Missense_Mutation	C	T	P8T3
FOXF1	chr16	86545001	Missense_Mutation	G	A	P8T3
TP53	chr17	7577130	Missense_Mutation	A	C	P8T3
CEBPB	chr20	48808357	Missense_Mutation	G	T	P8T3
GNAS	chr20	57478587	Missense_Mutation	G	A	P8T3
RPS6KA3	chrX	20284693	Missense_Mutation	C	T	P8T3
SUPT20HL2	chrX	24329641	Missense_Mutation	G	A	P8T3
ARR3	chrX	69496075	Missense_Mutation	C	A	P8T3
TCEAL2	chrX	101381971	Missense_Mutation	C	A	P8T3
EGFR	chr7	55242487	In_Frame_Del	TAAGAGAAGCAACATCTC	-	P8T3
TBC1D13	chr9	131565669	In_Frame_Del	GCTTATGTG	-	P8T3
SPG11	chr15	44859639	Frame_Shift_Del	TCAA	-	P8T3
SRPX2	chrX	99919846	In_Frame_Del	CTCTCG	-	P8T3
MUC2	chr11	1092889	Missense_Mutation	A	T	P9N
SUPT5H	chr19	39950553	Missense_Mutation	G	A	P9N
TERT	chr5	1294665	Frame_Shift_Del	G	-	P9N
OR4C5	chr11	48387286	Frame_Shift_Ins	-	GTCTTTAGTAG	P9N
EPB41L2	chr6	131184800	Missense_Mutation	T	C	P9T1
HOXA3	chr7	27150189	Missense_Mutation	A	C	P9T1
EGFR	chr7	55259515	Missense_Mutation	T	G	P9T1
SLC4A2	chr7	150763818	Missense_Mutation	C	G	P9T1
RIMS2	chr8	104664867	Missense_Mutation	T	G	P9T1
SREBF1	chr17	17740124	Missense_Mutation	T	A	P9T1
RBM10	chrX	47041725	Missense_Mutation	G	C	P9T1
TMCO4	chr1	20027310	Missense_Mutation	C	T	P9T2
CLK2	chr1	155239364	Missense_Mutation	T	C	P9T2
ACTN2	chr1	236881255	Missense_Mutation	T	C	P9T2

OR2L5	chr1	248185717	Nonsense_Mutation	T	A	P9T2
SLC9A4	chr2	103095615	Missense_Mutation	A	C	P9T2
DDX18	chr2	118579440	Missense_Mutation	A	G	P9T2
SCN1A	chr2	166896100	Missense_Mutation	T	C	P9T2
LRP2	chr2	170063706	Missense_Mutation	C	T	P9T2
CSRNP1	chr3	39186703	Missense_Mutation	C	A	P9T2
PTPRG	chr3	62118276	Missense_Mutation	G	A	P9T2
PDS5A	chr4	39929675	Missense_Mutation	T	C	P9T2
FBXW7	chr4	153250823	Splice_Site	C	T	P9T2
DCHS2	chr4	155411318	Missense_Mutation	A	G	P9T2
BRD9	chr5	891867	Missense_Mutation	T	C	P9T2
HAVCR1	chr5	156482457	Missense_Mutation	A	T	P9T2
RWDD2A	chr6	83904206	Missense_Mutation	G	C	P9T2
CBLL1	chr7	107398899	Missense_Mutation	A	T	P9T2
OR2A14	chr7	143826530	Missense_Mutation	G	A	P9T2
MCM4	chr8	48878767	Missense_Mutation	A	G	P9T2
SNX16	chr8	82736125	Missense_Mutation	C	A	P9T2
FRMPD1	chr9	37744956	Nonsense_Mutation	C	A	P9T2
SH3GLB2	chr9	131771452	Missense_Mutation	C	T	P9T2
MUC2	chr11	1092889	Missense_Mutation	A	T	P9T2
COPB1	chr11	14498512	Missense_Mutation	C	T	P9T2
GAS2	chr11	22833367	Missense_Mutation	G	T	P9T2
ARRB1	chr11	74979990	Missense_Mutation	C	T	P9T2
ZC3H12C	chr11	110036071	Missense_Mutation	C	G	P9T2
PUS7L	chr12	44124459	Missense_Mutation	T	C	P9T2
KRT5	chr12	52913603	Missense_Mutation	T	C	P9T2
PPP1R12A	chr12	80199984	Missense_Mutation	C	G	P9T2
SLC25A3	chr12	98987790	Missense_Mutation	A	C	P9T2
SLC7A8	chr14	23612372	Missense_Mutation	T	G	P9T2
RALGAPA1	chr14	36041924	Missense_Mutation	A	C	P9T2
LTBP2	chr14	75019694	Missense_Mutation	C	T	P9T2
PHKB	chr16	47727348	Missense_Mutation	A	G	P9T2
DNAI2	chr17	72278087	Missense_Mutation	G	A	P9T2
USH1G	chr17	72916503	Missense_Mutation	G	A	P9T2
PGS1	chr17	76388637	Missense_Mutation	A	T	P9T2
HEXDC	chr17	80386477	Missense_Mutation	G	A	P9T2
PLAUR	chr19	44153095	Missense_Mutation	T	C	P9T2
ZNF543	chr19	57839366	Missense_Mutation	A	G	P9T2
SPECC1L	chr22	24717940	Missense_Mutation	A	G	P9T2
PARVB	chr22	44553914	Missense_Mutation	T	G	P9T2
LMF2	chr22	50944499	Missense_Mutation	T	C	P9T2
TMSB4Y	chrY	15816243	Missense_Mutation	A	C	P9T2
EGFR	chr7	55242481	In_Frame_Del	AATTAAGA GAAGCAA	-	P9T2

AKT2	chr19	40762872	Frame_Shift_Del	C	-	P9T2
USP29	chr19	57641290	Frame_Shift_Del	C	-	P9T2
FBXO7	chr22	32894274	Frame_Shift_Ins	-	A	P9T2
CHD5	chr1	6214926	Missense_Mutation	G	T	P9T3
TMEM201	chr1	9658511	Missense_Mutation	G	C	P9T3
GPN2	chr1	27216470	Missense_Mutation	C	A	P9T3
PTPRF	chr1	44057090	Missense_Mutation	A	C	P9T3
ZSWIM5	chr1	45484448	Missense_Mutation	C	G	P9T3
PLPPR4	chr1	99753580	Missense_Mutation	C	A	P9T3
PDE4DIP	chr1	144871849	Missense_Mutation	T	C	P9T3
LCE3E	chr1	152538509	Missense_Mutation	C	T	P9T3
LCE2D	chr1	152636709	Missense_Mutation	C	A	P9T3
TTC24	chr1	156552154	Missense_Mutation	G	A	P9T3
BRINP3	chr1	190250725	Missense_Mutation	T	A	P9T3
TTC13	chr1	231069534	Missense_Mutation	T	G	P9T3
KLHL29	chr2	23865709	Missense_Mutation	G	A	P9T3
RTN4	chr2	55252749	Missense_Mutation	G	C	P9T3
TMEM131	chr2	98411492	Missense_Mutation	T	C	P9T3
C2orf40	chr2	106694362	Missense_Mutation	G	A	P9T3
CXCR4	chr2	136873101	Missense_Mutation	C	A	P9T3
ARIH2	chr3	49008116	Missense_Mutation	A	G	P9T3
CLDN18	chr3	137729185	Missense_Mutation	G	A	P9T3
SUCNR1	chr3	151598623	Missense_Mutation	A	C	P9T3
OTULIN	chr5	14681589	Missense_Mutation	C	A	P9T3
ADAMTS12	chr5	33881361	Missense_Mutation	T	C	P9T3
ACOT13	chr6	24698204	Missense_Mutation	T	G	P9T3
ITPR3	chr6	33652658	Missense_Mutation	A	G	P9T3
EYS	chr6	66094316	Missense_Mutation	T	A	P9T3
EGFR	chr7	55259515	Missense_Mutation	T	G	P9T3
DNAJC30	chr7	73097548	Missense_Mutation	T	G	P9T3
CALCR	chr7	93106974	Missense_Mutation	T	C	P9T3
MUC12	chr7	100635288	Missense_Mutation	G	A	P9T3
ZNF862	chr7	149545385	Missense_Mutation	C	T	P9T3
CTSB	chr8	11704606	Nonsense_Mutation	C	A	P9T3
FBXO18	chr10	5948107	Missense_Mutation	T	C	P9T3
ZCCHC24	chr10	81154045	Missense_Mutation	T	A	P9T3
ADRA2A	chr10	112839073	Missense_Mutation	T	C	P9T3
QSER1	chr11	32954070	Missense_Mutation	G	C	P9T3
OR8U1	chr11	56143728	Missense_Mutation	C	G	P9T3
PRICKLE1	chr12	42858560	Missense_Mutation	T	C	P9T3
TMPRSS12	chr12	51237759	Missense_Mutation	G	C	P9T3
KRT73	chr12	53004543	Missense_Mutation	A	T	P9T3
KRT73	chr12	53004544	Missense_Mutation	G	C	P9T3
PAN3	chr13	28771400	Missense_Mutation	A	G	P9T3

CCNB1IP1	chr14	20779822	Missense_Mutation	C	A	P9T3
RGS6	chr14	72945015	Missense_Mutation	A	G	P9T3
MKL2	chr16	14341332	Missense_Mutation	G	A	P9T3
CCP110	chr16	19547589	Missense_Mutation	A	G	P9T3
CNOT1	chr16	58572691	Missense_Mutation	T	C	P9T3
CARMIL2	chr16	67683474	Missense_Mutation	C	T	P9T3
ST3GAL2	chr16	70415600	Missense_Mutation	C	T	P9T3
CLEC4G	chr19	7795285	Missense_Mutation	C	T	P9T3
NXNL1	chr19	17571504	Missense_Mutation	C	T	P9T3
DMKN	chr19	36004200	Missense_Mutation	C	T	P9T3
GGTLC1	chr20	23966797	Missense_Mutation	T	G	P9T3
CHD6	chr20	40054739	Missense_Mutation	G	A	P9T3
EEF1A2	chr20	62121918	Missense_Mutation	C	T	P9T3
GGT1	chr22	25011007	Splice_Site	G	C	P9T3
ASPHD2	chr22	26839093	Missense_Mutation	T	C	P9T3
MTFP1	chr22	30823263	Missense_Mutation	A	C	P9T3
NDUFB11	chrX	47002130	Missense_Mutation	T	C	P9T3
KIF4B	chr5	154395760	Frame_Shift_Del	C	-	P9T3
ZNF623	chr8	144732356	Frame_Shift_Del	T	-	P9T3
OR4C5	chr11	48387286	Frame_Shift_Ins	-	GTCTTTAGTAG	P9T3
PPP2R5E	chr14	63920469	Frame_Shift_Del	TC	-	P9T3
RBM10	chrX	47038773	Frame_Shift_Del	ACTG	-	P9T3
EPHA8	chr1	22915660	Missense_Mutation	C	T	P10T1
CCDC24	chr1	44457892	Missense_Mutation	G	A	P10T1
GPSM2	chr1	109440650	Missense_Mutation	C	T	P10T1
CD1A	chr1	158226716	Nonsense_Mutation	C	T	P10T1
ALMS1	chr2	73828493	Missense_Mutation	G	A	P10T1
TMEM177	chr2	120438755	Missense_Mutation	G	A	P10T1
TMEM177	chr2	120438756	Missense_Mutation	T	G	P10T1
TMEM177	chr2	120438758	Missense_Mutation	T	C	P10T1
LOC200726	chr2	207508970	Missense_Mutation	C	A	P10T1
RAB6B	chr3	133557035	Missense_Mutation	G	T	P10T1
PTPN13	chr4	87662844	Nonsense_Mutation	A	T	P10T1
MAP9	chr4	156294399	Missense_Mutation	T	C	P10T1
TRIM60	chr4	165961379	Missense_Mutation	C	A	P10T1
EGFR	chr7	55259515	Missense_Mutation	T	G	P10T1
NRCAM	chr7	107830110	Missense_Mutation	C	T	P10T1
PIGO	chr9	35091449	Nonsense_Mutation	A	C	P10T1
ERCC6L2	chr9	98638349	Missense_Mutation	C	G	P10T1
SNAPC4	chr9	139273626	Missense_Mutation	C	T	P10T1
ARFGAP2	chr11	47198071	Missense_Mutation	T	C	P10T1
TMEM132C	chr12	128900108	Missense_Mutation	T	G	P10T1
TMEM132C	chr12	128900111	Missense_Mutation	T	G	P10T1
KCNH5	chr14	63174625	Missense_Mutation	G	T	P10T1

FLRT2	chr14	86088603	Missense_Mutation	C	A	P10T1
ACAN	chr15	89402370	Missense_Mutation	C	T	P10T1
IRGQ	chr19	44096447	Missense_Mutation	C	A	P10T1
HSCB	chr22	29141934	Missense_Mutation	A	G	P10T1
SH3KBP1	chrX	19554577	Splice_Site	C	T	P10T1
RLIM	chrX	73811792	Missense_Mutation	C	T	P10T1
TENM1	chrX	123526055	Nonsense_Mutation	A	T	P10T1
MELK	chr9	36665488	Frame_Shift_Del	TA	-	P10T1
FAM120A	chr9	96305727	Frame_Shift_Del	C	-	P10T1
VSIG10L2	chr11	125821904	Frame_Shift_Ins	-	A	P10T1
LGR5	chr12	71960482	Splice_Site	-	T	P10T1
PIGM	chr1	160001012	Missense_Mutation	T	C	P10N
AGAP1	chr2	236659094	Missense_Mutation	C	G	P10N
PXK	chr3	58376354	Missense_Mutation	A	C	P10N
PLOD2	chr3	145803047	Nonsense_Mutation	G	A	P10N
FAM193A	chr4	2632729	Nonsense_Mutation	G	T	P10N
DDX60	chr4	169158461	Missense_Mutation	C	A	P10N
RAI14	chr5	34796093	Missense_Mutation	A	T	P10N
AFDN	chr6	168352541	Missense_Mutation	C	G	P10N
C1GALT1	chr7	7274059	Missense_Mutation	A	T	P10N
SMO	chr7	128845460	Missense_Mutation	G	A	P10N
PLEC	chr8	144991561	Missense_Mutation	A	G	P10N
CPSF1	chr8	145623931	Missense_Mutation	C	T	P10N
PTCH1	chr9	98231215	Missense_Mutation	C	T	P10N
LAMC3	chr9	133954657	Missense_Mutation	C	T	P10N
MUC6	chr11	1017945	Missense_Mutation	A	G	P10N
EIF4G2	chr11	10822040	Missense_Mutation	G	A	P10N
PRR5L	chr11	36472856	Missense_Mutation	G	A	P10N
NAALADL1	chr11	64813322	Missense_Mutation	T	C	P10N
TMPRSS13	chr11	117776404	Missense_Mutation	G	A	P10N
SLCO1A2	chr12	21467532	Missense_Mutation	T	A	P10N
PARP4	chr13	25058867	Missense_Mutation	C	T	P10N
TNRC6C	chr17	76089802	Missense_Mutation	A	G	P10N
GIPC3	chr19	3586847	Missense_Mutation	G	C	P10N
ZNF493	chr19	21606828	Missense_Mutation	C	T	P10N
ZNF729	chr19	22498782	Missense_Mutation	G	C	P10N
PAK4	chr19	39663601	Missense_Mutation	C	T	P10N
ZNF418	chr19	58439369	Missense_Mutation	G	T	P10N
RALGAPA2	chr20	20475846	Missense_Mutation	A	C	P10N
ZDHHC8	chr22	20126742	Missense_Mutation	T	C	P10N
SBF1	chr22	50901038	Missense_Mutation	G	A	P10N
RLIM	chrX	73811792	Missense_Mutation	C	T	P10N
HSFX4	chrX	149098227	Missense_Mutation	G	A	P10N
IRF2BP2	chr1	234744234	Frame_Shift_Ins	-	A	P10N

UPF2	chr10	11971879	Frame_Shift_Ins	-	T	P10N
NPRL3	chr16	188335	Splice_Site	GAGGAGG ACGGAGCC GGAGGCG GAGGGGG CCT	-	P10N
EPHA8	chr1	22915660	Missense_Mutation	C	T	P10T2
CCDC24	chr1	44457892	Missense_Mutation	G	A	P10T2
GPSM2	chr1	109440650	Missense_Mutation	C	T	P10T2
LEFTY1	chr1	226075708	Missense_Mutation	A	G	P10T2
OR2B11	chr1	247615200	Missense_Mutation	G	T	P10T2
ALMS1	chr2	73828493	Missense_Mutation	G	A	P10T2
TMEM177	chr2	120438755	Missense_Mutation	G	A	P10T2
TMEM177	chr2	120438756	Missense_Mutation	T	G	P10T2
TMEM177	chr2	120438758	Missense_Mutation	T	C	P10T2
LOC200726	chr2	207508970	Missense_Mutation	C	A	P10T2
PRSS56	chr2	233386684	Missense_Mutation	C	A	P10T2
SLC12A8	chr3	124802757	Missense_Mutation	C	G	P10T2
RAB6B	chr3	133557035	Missense_Mutation	G	T	P10T2
ZIC4	chr3	147114239	Missense_Mutation	G	A	P10T2
MAP9	chr4	156294399	Missense_Mutation	T	C	P10T2
TRIM60	chr4	165961379	Missense_Mutation	C	A	P10T2
CYP4V2	chr4	187126444	Missense_Mutation	G	T	P10T2
AP3B1	chr5	77396850	Splice_Site	C	T	P10T2
EGFR	chr7	55242511	Missense_Mutation	G	T	P10T2
EGFR	chr7	55259515	Missense_Mutation	T	G	P10T2
SLC26A3	chr7	107408041	Missense_Mutation	G	A	P10T2
NRCAM	chr7	107830110	Missense_Mutation	C	T	P10T2
TTC26	chr7	138854041	Missense_Mutation	C	T	P10T2
ZNF783	chr7	148978673	Nonsense_Mutation	C	T	P10T2
BNC2	chr9	16832280	Missense_Mutation	C	T	P10T2
TAF1L	chr9	32634109	Missense_Mutation	T	C	P10T2
C9orf24	chr9	34382812	Missense_Mutation	C	T	P10T2
ERCC6L2	chr9	98638349	Missense_Mutation	C	G	P10T2
SNAPC4	chr9	139273626	Missense_Mutation	C	T	P10T2
TOR4A	chr9	140173317	Missense_Mutation	G	A	P10T2
PFKP	chr10	3109819	Missense_Mutation	G	A	P10T2
MBL2	chr10	54528079	Missense_Mutation	C	T	P10T2
HPSE2	chr10	100219432	Missense_Mutation	G	T	P10T2
ARFGAP2	chr11	47198071	Missense_Mutation	T	C	P10T2
ADAMTS20	chr12	43770101	Missense_Mutation	C	T	P10T2
FBRSL1	chr12	133084787	Missense_Mutation	C	T	P10T2
KCNH5	chr14	63174625	Missense_Mutation	G	T	P10T2
FLRT2	chr14	86088603	Missense_Mutation	C	A	P10T2

ACAN	chr15	89402370	Missense_Mutation	C	T	P10T2
FBXL16	chr16	745469	Missense_Mutation	G	A	P10T2
MYCBPAP	chr17	48594027	Missense_Mutation	C	T	P10T2
APCDD1	chr18	10471855	Nonsense_Mutation	C	T	P10T2
SYT4	chr18	40850467	Missense_Mutation	C	T	P10T2
MAPK4	chr18	48190513	Missense_Mutation	C	T	P10T2
IRGQ	chr19	44096447	Missense_Mutation	C	A	P10T2
ZNF835	chr19	57175257	Missense_Mutation	T	C	P10T2
SEMG1	chr20	43836514	Missense_Mutation	C	A	P10T2
OSBPL2	chr20	60835158	Missense_Mutation	A	T	P10T2
HSCB	chr22	29141934	Missense_Mutation	A	G	P10T2
RLIM	chrX	73811792	Missense_Mutation	C	T	P10T2
TENM1	chrX	123526055	Nonsense_Mutation	A	T	P10T2
SLITRK2	chrX	144906424	Missense_Mutation	A	T	P10T2
ZNF721	chr4	437966	In_Frame_Del	TTGTCTTA TCTTTGT	-	P10T2
MELK	chr9	36665488	Frame_Shift_Del	TA	-	P10T2
FAM120A	chr9	96305727	Frame_Shift_Del	C	-	P10T2
VSIG10L2	chr11	125821904	Frame_Shift_Ins	-	A	P10T2
LGR5	chr12	71960482	Splice_Site	-	T	P10T2
NPRL3	chr16	188335	Splice_Site	GAGGAGG ACGGAGCC GGAGGCG GAGGGGG CCT	-	P10T2
FRMD7	chrX	131212733	Frame_Shift_Ins	-	A	P10T2

Supplementary Table 3 Immune-related gene expression

Sample	Gene	Value
P1N	CCL5	8.03
P1T1	CCL5	15.17
P1T2	CCL5	18.26
P1T3	CCL5	25.58
P2N	CCL5	84.39
P2T1	CCL5	93.76
P2T2	CCL5	54.39
P2T3	CCL5	22.03
P3N	CCL5	60.15
P3T1	CCL5	7.1
P3T2	CCL5	20.3
P3T3	CCL5	34.78
P4N	CCL5	66.57
P4T1	CCL5	10.26
P4T2	CCL5	9.33
P4T3	CCL5	16.4
P5N	CCL5	9.68
P5T1	CCL5	30.6
P5T2	CCL5	8.45
P6N	CCL5	70.39
P6T1	CCL5	36.83
P6T2	CCL5	78.68
P6T3	CCL5	39.19
P7N	CCL5	17
P7T1	CCL5	9.75
P7T2	CCL5	50.78
P8N	CCL5	12.24
P8T1	CCL5	6.66
P8T2	CCL5	34.13
P8T3	CCL5	23.28
P9N	CCL5	9.45
P9T1	CCL5	5.7
P9T2	CCL5	1.18
P9T3	CCL5	28.4
P10N	CCL5	1.38
P10T1	CCL5	6.78
P10T2	CCL5	4.29
P1N	CD163	18.91
P1T1	CD163	41.72

P1T2	CD163	19.24
P1T3	CD163	14.19
P2N	CD163	52.67
P2T1	CD163	86.41
P2T2	CD163	69.52
P2T3	CD163	28.82
P3N	CD163	34
P3T1	CD163	6.62
P3T2	CD163	62.53
P3T3	CD163	50.15
P4N	CD163	71.56
P4T1	CD163	15.88
P4T2	CD163	5.56
P4T3	CD163	24.21
P5N	CD163	7.96
P5T1	CD163	24.06
P5T2	CD163	6.9
P6N	CD163	34.87
P6T1	CD163	93.54
P6T2	CD163	70.55
P6T3	CD163	50.43
P7N	CD163	18.15
P7T1	CD163	18.82
P7T2	CD163	27.37
P8N	CD163	14.7
P8T1	CD163	11.01
P8T2	CD163	64.77
P8T3	CD163	23.58
P9N	CD163	8.21
P9T1	CD163	8.96
P9T2	CD163	3.82
P9T3	CD163	42.99
P10N	CD163	0.86
P10T1	CD163	3.11
P10T2	CD163	11.48
P1N	CD19	0.08
P1T1	CD19	0.16
P1T2	CD19	0.25
P1T3	CD19	0.73
P2N	CD19	0.37
P2T1	CD19	0.42
P2T2	CD19	0.06
P2T3	CD19	0.27
P3N	CD19	0.49

P3T1	CD19	0.41
P3T2	CD19	0.22
P3T3	CD19	0.56
P4N	CD19	0.22
P4T1	CD19	0.09
P4T2	CD19	0.04
P4T3	CD19	0.34
P5N	CD19	0.04
P5T1	CD19	0.22
P5T2	CD19	0.03
P6N	CD19	0.27
P6T1	CD19	1.5
P6T2	CD19	0.51
P6T3	CD19	2
P7N	CD19	0
P7T1	CD19	0.37
P7T2	CD19	0.96
P8N	CD19	0.05
P8T1	CD19	0.06
P8T2	CD19	4.72
P8T3	CD19	1.45
P9N	CD19	0.23
P9T1	CD19	1.81
P9T2	CD19	0.26
P9T3	CD19	0.41
P10N	CD19	0.06
P10T1	CD19	1.45
P10T2	CD19	0.9
P1N	CD27	0.38
P1T1	CD27	3.59
P1T2	CD27	1.39
P1T3	CD27	3.27
P2N	CD27	2.24
P2T1	CD27	7.61
P2T2	CD27	1.85
P2T3	CD27	2.63
P3N	CD27	2.23
P3T1	CD27	2.27
P3T2	CD27	2.48
P3T3	CD27	4.01
P4N	CD27	2.42
P4T1	CD27	0.63
P4T2	CD27	0.74
P4T3	CD27	2.23

P5N	CD27	0.33
P5T1	CD27	3.8
P5T2	CD27	0.54
P6N	CD27	2.97
P6T1	CD27	6.22
P6T2	CD27	9.22
P6T3	CD27	8.55
P7N	CD27	0.43
P7T1	CD27	1.5
P7T2	CD27	6
P8N	CD27	0.43
P8T1	CD27	0.51
P8T2	CD27	11.43
P8T3	CD27	5.59
P9N	CD27	1.35
P9T1	CD27	3.2
P9T2	CD27	0.69
P9T3	CD27	5.82
P10N	CD27	0.33
P10T1	CD27	2.93
P10T2	CD27	1.26
P1N	CD274	1.98
P1T1	CD274	4.34
P1T2	CD274	4.14
P1T3	CD274	1.92
P2N	CD274	14.55
P2T1	CD274	7.93
P2T2	CD274	4.99
P2T3	CD274	2.98
P3N	CD274	3.56
P3T1	CD274	0.28
P3T2	CD274	3.42
P3T3	CD274	4.75
P4N	CD274	4.46
P4T1	CD274	0.24
P4T2	CD274	2.36
P4T3	CD274	1.78
P5N	CD274	1.26
P5T1	CD274	3.18
P5T2	CD274	1.28
P6N	CD274	2.15
P6T1	CD274	6.51
P6T2	CD274	3.22
P6T3	CD274	3.13

P7N	CD274	2.11
P7T1	CD274	0.86
P7T2	CD274	1.66
P8N	CD274	1.92
P8T1	CD274	1.7
P8T2	CD274	1.83
P8T3	CD274	1.99
P9N	CD274	1.66
P9T1	CD274	0.49
P9T2	CD274	0.11
P9T3	CD274	2.46
P10N	CD274	7.00E-02
P10T1	CD274	0.2
P10T2	CD274	0.99
P1N	CD4	12.8
P1T1	CD4	22
P1T2	CD4	25.15
P1T3	CD4	19.09
P2N	CD4	57.58
P2T1	CD4	77.17
P2T2	CD4	59.68
P2T3	CD4	27.93
P3N	CD4	50.11
P3T1	CD4	11.19
P3T2	CD4	48.46
P3T3	CD4	60.65
P4N	CD4	39.69
P4T1	CD4	15.13
P4T2	CD4	8.93
P4T3	CD4	18.5
P5N	CD4	6.16
P5T1	CD4	19
P5T2	CD4	7.47
P6N	CD4	28.14
P6T1	CD4	49.11
P6T2	CD4	50.38
P6T3	CD4	64.41
P7N	CD4	14.51
P7T1	CD4	10.41
P7T2	CD4	71.17
P8N	CD4	10.05
P8T1	CD4	10.18
P8T2	CD4	67.83
P8T3	CD4	34.31

P9N	CD4	11.84
P9T1	CD4	9.59
P9T2	CD4	6.06
P9T3	CD4	51.71
P10N	CD4	2.34
P10T1	CD4	11.31
P10T2	CD4	8.97
P1N	CD8A	1.15
P1T1	CD8A	2.91
P1T2	CD8A	3.8
P1T3	CD8A	4.26
P2N	CD8A	9.38
P2T1	CD8A	8.59
P2T2	CD8A	5.81
P2T3	CD8A	3.32
P3N	CD8A	7.04
P3T1	CD8A	3.78
P3T2	CD8A	2.23
P3T3	CD8A	4.12
P4N	CD8A	8.03
P4T1	CD8A	5.53
P4T2	CD8A	2.78
P4T3	CD8A	4.47
P5N	CD8A	2.17
P5T1	CD8A	5.6
P5T2	CD8A	2.14
P6N	CD8A	5.45
P6T1	CD8A	7.49
P6T2	CD8A	5.57
P6T3	CD8A	3.87
P7N	CD8A	4.71
P7T1	CD8A	3.43
P7T2	CD8A	10.07
P8N	CD8A	2.87
P8T1	CD8A	1.73
P8T2	CD8A	3.24
P8T3	CD8A	3.61
P9N	CD8A	2.56
P9T1	CD8A	2.14
P9T2	CD8A	1.84
P9T3	CD8A	4.46
P10N	CD8A	0.33
P10T1	CD8A	0.81
P10T2	CD8A	1.5

P1N	CR2	0
P1T1	CR2	0.11
P1T2	CR2	0.01
P1T3	CR2	0.56
P2N	CR2	7.00E-02
P2T1	CR2	0.06
P2T2	CR2	0.02
P2T3	CR2	0.03
P3N	CR2	0.01
P3T1	CR2	0.08
P3T2	CR2	0.05
P3T3	CR2	0.06
P4N	CR2	0.01
P4T1	CR2	0.03
P4T2	CR2	0.01
P4T3	CR2	0.79
P5N	CR2	0.03
P5T1	CR2	0.53
P5T2	CR2	0.05
P6N	CR2	0.01
P6T1	CR2	1.44
P6T2	CR2	0.11
P6T3	CR2	1.74
P7N	CR2	0
P7T1	CR2	0.41
P7T2	CR2	0.59
P8N	CR2	0
P8T1	CR2	0.38
P8T2	CR2	7.26
P8T3	CR2	2.59
P9N	CR2	0.06
P9T1	CR2	1.85
P9T2	CR2	0.29
P9T3	CR2	0.05
P10N	CR2	0
P10T1	CR2	0.96
P10T2	CR2	1.09
P1N	CMKLR1	4.59
P1T1	CMKLR1	4.56
P1T2	CMKLR1	10.56
P1T3	CMKLR1	7.36
P2N	CMKLR1	9.78
P2T1	CMKLR1	6.23
P2T2	CMKLR1	9.15

P2T3	CMKLR1	7.5
P3N	CMKLR1	6.46
P3T1	CMKLR1	4.31
P3T2	CMKLR1	2.27
P3T3	CMKLR1	4.52
P4N	CMKLR1	5.31
P4T1	CMKLR1	10.69
P4T2	CMKLR1	4.16
P4T3	CMKLR1	6.68
P5N	CMKLR1	5.44
P5T1	CMKLR1	1.61
P5T2	CMKLR1	4.41
P6N	CMKLR1	7.94
P6T1	CMKLR1	9.33
P6T2	CMKLR1	8.25
P6T3	CMKLR1	3.94
P7N	CMKLR1	8.82
P7T1	CMKLR1	2.16
P7T2	CMKLR1	7.52
P8N	CMKLR1	5.86
P8T1	CMKLR1	2.35
P8T2	CMKLR1	4.9
P8T3	CMKLR1	1.64
P9N	CMKLR1	6.04
P9T1	CMKLR1	3.08
P9T2	CMKLR1	0.75
P9T3	CMKLR1	6.65
P10N	CMKLR1	1.77
P10T1	CMKLR1	1.17
P10T2	CMKLR1	2.72
P1N	CTLA4	0.29
P1T1	CTLA4	0.99
P1T2	CTLA4	0.54
P1T3	CTLA4	1.57
P2N	CTLA4	1.07
P2T1	CTLA4	1.21
P2T2	CTLA4	0.84
P2T3	CTLA4	0.95
P3N	CTLA4	0.35
P3T1	CTLA4	0.9
P3T2	CTLA4	0.62
P3T3	CTLA4	0.6
P4N	CTLA4	0.25
P4T1	CTLA4	0.47

P4T2	CTLA4	0.5
P4T3	CTLA4	0.94
P5N	CTLA4	0.32
P5T1	CTLA4	1.18
P5T2	CTLA4	1.5
P6N	CTLA4	0.49
P6T1	CTLA4	1.37
P6T2	CTLA4	1.06
P6T3	CTLA4	1.99
P7N	CTLA4	0.41
P7T1	CTLA4	1.49
P7T2	CTLA4	0.57
P8N	CTLA4	0.12
P8T1	CTLA4	0.82
P8T2	CTLA4	1.46
P8T3	CTLA4	1.33
P9N	CTLA4	0.66
P9T1	CTLA4	0.54
P9T2	CTLA4	0.16
P9T3	CTLA4	0.37
P10N	CTLA4	0
P10T1	CTLA4	0.32
P10T2	CTLA4	0.68
P1N	CX3CR1	5.12
P1T1	CX3CR1	6.14
P1T2	CX3CR1	11.74
P1T3	CX3CR1	8.52
P2N	CX3CR1	10.76
P2T1	CX3CR1	3.16
P2T2	CX3CR1	6.2
P2T3	CX3CR1	2.1
P3N	CX3CR1	5.63
P3T1	CX3CR1	1.11
P3T2	CX3CR1	1.53
P3T3	CX3CR1	5.1
P4N	CX3CR1	5.18
P4T1	CX3CR1	0.68
P4T2	CX3CR1	2.68
P4T3	CX3CR1	1.28
P5N	CX3CR1	5.85
P5T1	CX3CR1	2.16
P5T2	CX3CR1	2.79
P6N	CX3CR1	1.9
P6T1	CX3CR1	7.17

P6T2	CX3CR1	5.32
P6T3	CX3CR1	1.17
P7N	CX3CR1	0.72
P7T1	CX3CR1	0.3
P7T2	CX3CR1	1.87
P8N	CX3CR1	1.82
P8T1	CX3CR1	0.99
P8T2	CX3CR1	1.06
P8T3	CX3CR1	1.17
P9N	CX3CR1	4.19
P9T1	CX3CR1	0.39
P9T2	CX3CR1	0.25
P9T3	CX3CR1	3.19
P10N	CX3CR1	0.36
P10T1	CX3CR1	0.5
P10T2	CX3CR1	0.86
P1N	CXCL13	0.06
P1T1	CXCL13	0.09
P1T2	CXCL13	0.04
P1T3	CXCL13	0.26
P2N	CXCL13	0.09
P2T1	CXCL13	0.27
P2T2	CXCL13	0.46
P2T3	CXCL13	0.12
P3N	CXCL13	0.05
P3T1	CXCL13	0.09
P3T2	CXCL13	0.14
P3T3	CXCL13	0.59
P4N	CXCL13	0.02
P4T1	CXCL13	0
P4T2	CXCL13	0.08
P4T3	CXCL13	5.25
P5N	CXCL13	0
P5T1	CXCL13	2.99
P5T2	CXCL13	0.05
P6N	CXCL13	0.12
P6T1	CXCL13	13
P6T2	CXCL13	1.38
P6T3	CXCL13	16.44
P7N	CXCL13	0.02
P7T1	CXCL13	2.59
P7T2	CXCL13	10.61
P8N	CXCL13	0.02
P8T1	CXCL13	0.02

P8T2	CXCL13	17.64
P8T3	CXCL13	24.53
P9N	CXCL13	0.19
P9T1	CXCL13	4.28
P9T2	CXCL13	0.44
P9T3	CXCL13	0.34
P10N	CXCL13	0.04
P10T1	CXCL13	26.01
P10T2	CXCL13	7.21
P1N	CXCR6	2.02
P1T1	CXCR6	3.13
P1T2	CXCR6	4.17
P1T3	CXCR6	2.61
P2N	CXCR6	4.16
P2T1	CXCR6	5.61
P2T2	CXCR6	2.73
P2T3	CXCR6	4.28
P3N	CXCR6	1.94
P3T1	CXCR6	1.46
P3T2	CXCR6	1.81
P3T3	CXCR6	2.53
P4N	CXCR6	1.61
P4T1	CXCR6	1.02
P4T2	CXCR6	3.4
P4T3	CXCR6	4.15
P5N	CXCR6	2.44
P5T1	CXCR6	2.57
P5T2	CXCR6	2.31
P6N	CXCR6	2.52
P6T1	CXCR6	5.68
P6T2	CXCR6	4.46
P6T3	CXCR6	4.04
P7N	CXCR6	2.85
P7T1	CXCR6	1.93
P7T2	CXCR6	3.52
P8N	CXCR6	1.56
P8T1	CXCR6	1.19
P8T2	CXCR6	0.9
P8T3	CXCR6	1.61
P9N	CXCR6	2.61
P9T1	CXCR6	1.12
P9T2	CXCR6	0.35
P9T3	CXCR6	1.34
P10N	CXCR6	0.06

P10T1	CXCR6	0.15
P10T2	CXCR6	1.75
P1N	FGFBP2	1.23
P1T1	FGFBP2	1.18
P1T2	FGFBP2	3.65
P1T3	FGFBP2	1.56
P2N	FGFBP2	16.7
P2T1	FGFBP2	3.79
P2T2	FGFBP2	7.39
P2T3	FGFBP2	0.6
P3N	FGFBP2	12.4
P3T1	FGFBP2	0.2
P3T2	FGFBP2	0.77
P3T3	FGFBP2	1.97
P4N	FGFBP2	7.57
P4T1	FGFBP2	0.06
P4T2	FGFBP2	0.29
P4T3	FGFBP2	0
P5N	FGFBP2	3.84
P5T1	FGFBP2	2.33
P5T2	FGFBP2	1.55
P6N	FGFBP2	8.73
P6T1	FGFBP2	9.3
P6T2	FGFBP2	11.67
P6T3	FGFBP2	1.59
P7N	FGFBP2	0.68
P7T1	FGFBP2	7.00E-02
P7T2	FGFBP2	0.41
P8N	FGFBP2	0.47
P8T1	FGFBP2	0.46
P8T2	FGFBP2	0.22
P8T3	FGFBP2	0.23
P9N	FGFBP2	1.71
P9T1	FGFBP2	0.05
P9T2	FGFBP2	0.08
P9T3	FGFBP2	4.27
P10N	FGFBP2	0.19
P10T1	FGFBP2	0.42
P10T2	FGFBP2	0.03
P1N	FOXP3	0.24
P1T1	FOXP3	0.89
P1T2	FOXP3	0.69
P1T3	FOXP3	2.14
P2N	FOXP3	0.61

P2T1	FOXP3	1.04
P2T2	FOXP3	0.38
P2T3	FOXP3	0.85
P3N	FOXP3	0.54
P3T1	FOXP3	0.75
P3T2	FOXP3	0.95
P3T3	FOXP3	0.97
P4N	FOXP3	0.48
P4T1	FOXP3	0.44
P4T2	FOXP3	0.65
P4T3	FOXP3	1.2
P5N	FOXP3	0.11
P5T1	FOXP3	0.24
P5T2	FOXP3	0.42
P6N	FOXP3	0.4
P6T1	FOXP3	0.99
P6T2	FOXP3	0.76
P6T3	FOXP3	1.2
P7N	FOXP3	0.39
P7T1	FOXP3	0.66
P7T2	FOXP3	0.74
P8N	FOXP3	0.52
P8T1	FOXP3	1.04
P8T2	FOXP3	3.25
P8T3	FOXP3	1.96
P9N	FOXP3	0.56
P9T1	FOXP3	1.1
P9T2	FOXP3	0.24
P9T3	FOXP3	0.57
P10N	FOXP3	0.04
P10T1	FOXP3	0.34
P10T2	FOXP3	1.14
P1N	GNLY	3.08
P1T1	GNLY	7.07
P1T2	GNLY	14.17
P1T3	GNLY	17.01
P2N	GNLY	51.03
P2T1	GNLY	17.76
P2T2	GNLY	20.33
P2T3	GNLY	5.11
P3N	GNLY	45.2
P3T1	GNLY	3.26
P3T2	GNLY	7.68
P3T3	GNLY	12.06

P4N	GNLY	47.43
P4T1	GNLY	2.83
P4T2	GNLY	1.49
P4T3	GNLY	2.58
P5N	GNLY	18.03
P5T1	GNLY	17.25
P5T2	GNLY	14.15
P6N	GNLY	54.58
P6T1	GNLY	15.09
P6T2	GNLY	41.08
P6T3	GNLY	5.23
P7N	GNLY	6.82
P7T1	GNLY	0.89
P7T2	GNLY	3.78
P8N	GNLY	5.65
P8T1	GNLY	4.22
P8T2	GNLY	7.58
P8T3	GNLY	1.74
P9N	GNLY	6.85
P9T1	GNLY	1.18
P9T2	GNLY	1.62
P9T3	GNLY	14.42
P10N	GNLY	0.67
P10T1	GNLY	0.5
P10T2	GNLY	1.01
P1N	GZMB	4.35
P1T1	GZMB	5.75
P1T2	GZMB	10.62
P1T3	GZMB	7.58
P2N	GZMB	38.03
P2T1	GZMB	13.75
P2T2	GZMB	16.93
P2T3	GZMB	1.33
P3N	GZMB	22.15
P3T1	GZMB	0.41
P3T2	GZMB	4.63
P3T3	GZMB	5.72
P4N	GZMB	17.21
P4T1	GZMB	0.2
P4T2	GZMB	0.58
P4T3	GZMB	0.59
P5N	GZMB	4.16
P5T1	GZMB	3.93
P5T2	GZMB	2.01

P6N	GZMB	31.44
P6T1	GZMB	12.79
P6T2	GZMB	24.88
P6T3	GZMB	4.73
P7N	GZMB	1.95
P7T1	GZMB	1.03
P7T2	GZMB	4.19
P8N	GZMB	0.93
P8T1	GZMB	0.88
P8T2	GZMB	5.21
P8T3	GZMB	2.5
P9N	GZMB	1.91
P9T1	GZMB	0.09
P9T2	GZMB	1.21
P9T3	GZMB	9.08
P10N	GZMB	0.23
P10T1	GZMB	0.91
P10T2	GZMB	0.44
P1N	GZMH	0.54
P1T1	GZMH	2.22
P1T2	GZMH	3.51
P1T3	GZMH	2.72
P2N	GZMH	32.12
P2T1	GZMH	14.63
P2T2	GZMH	17.59
P2T3	GZMH	1.02
P3N	GZMH	21.28
P3T1	GZMH	0.36
P3T2	GZMH	2.72
P3T3	GZMH	5.17
P4N	GZMH	12.8
P4T1	GZMH	0.44
P4T2	GZMH	0.83
P4T3	GZMH	0.73
P5N	GZMH	6.42
P5T1	GZMH	9.33
P5T2	GZMH	2.48
P6N	GZMH	23.69
P6T1	GZMH	12.29
P6T2	GZMH	24.15
P6T3	GZMH	4.16
P7N	GZMH	1.92
P7T1	GZMH	0.34
P7T2	GZMH	3.74

P8N	GZMH	2
P8T1	GZMH	1.08
P8T2	GZMH	1.12
P8T3	GZMH	1.84
P9N	GZMH	1.31
P9T1	GZMH	0.39
P9T2	GZMH	0.55
P9T3	GZMH	7.78
P10N	GZMH	0.53
P10T1	GZMH	0.88
P10T2	GZMH	0.21
P1N	HAVCR2	6.74
P1T1	HAVCR2	10.78
P1T2	HAVCR2	8.75
P1T3	HAVCR2	5.52
P2N	HAVCR2	8.33
P2T1	HAVCR2	13.11
P2T2	HAVCR2	10.91
P2T3	HAVCR2	7.99
P3N	HAVCR2	6.58
P3T1	HAVCR2	1.58
P3T2	HAVCR2	10.25
P3T3	HAVCR2	12.85
P4N	HAVCR2	14.93
P4T1	HAVCR2	3.38
P4T2	HAVCR2	3.02
P4T3	HAVCR2	8.26
P5N	HAVCR2	4.69
P5T1	HAVCR2	7.02
P5T2	HAVCR2	3.15
P6N	HAVCR2	6.92
P6T1	HAVCR2	12.64
P6T2	HAVCR2	7.98
P6T3	HAVCR2	8.86
P7N	HAVCR2	2.23
P7T1	HAVCR2	2.51
P7T2	HAVCR2	8.3
P8N	HAVCR2	4.15
P8T1	HAVCR2	3.12
P8T2	HAVCR2	27.32
P8T3	HAVCR2	5.89
P9N	HAVCR2	3.62
P9T1	HAVCR2	1.98
P9T2	HAVCR2	1.26

P9T3	HAVCR2	6.9
P10N	HAVCR2	0.89
P10T1	HAVCR2	2.45
P10T2	HAVCR2	4.53
P1N	IDO1	0.22
P1T1	IDO1	0.77
P1T2	IDO1	0.42
P1T3	IDO1	1.21
P2N	IDO1	30.69
P2T1	IDO1	9.92
P2T2	IDO1	22.04
P2T3	IDO1	1.85
P3N	IDO1	8.69
P3T1	IDO1	0.21
P3T2	IDO1	1.87
P3T3	IDO1	2.6
P4N	IDO1	33.26
P4T1	IDO1	0.61
P4T2	IDO1	1.66
P4T3	IDO1	0.84
P5N	IDO1	1.44
P5T1	IDO1	0.7
P5T2	IDO1	1.09
P6N	IDO1	6.83
P6T1	IDO1	6.5
P6T2	IDO1	13.97
P6T3	IDO1	2.29
P7N	IDO1	1.26
P7T1	IDO1	0.71
P7T2	IDO1	3.64
P8N	IDO1	2.15
P8T1	IDO1	1.45
P8T2	IDO1	2.15
P8T3	IDO1	1.59
P9N	IDO1	1.22
P9T1	IDO1	0.14
P9T2	IDO1	0.08
P9T3	IDO1	5.68
P10N	IDO1	0.9
P10T1	IDO1	0.7
P10T2	IDO1	0.99
P1N	LAG3	0.55
P1T1	LAG3	0.74
P1T2	LAG3	1.04

P1T3	LAG3	2.05
P2N	LAG3	4.26
P2T1	LAG3	3.7
P2T2	LAG3	2.36
P2T3	LAG3	1.75
P3N	LAG3	2.46
P3T1	LAG3	1.19
P3T2	LAG3	1.23
P3T3	LAG3	2.42
P4N	LAG3	2.65
P4T1	LAG3	2.45
P4T2	LAG3	1.81
P4T3	LAG3	1.24
P5N	LAG3	0.5
P5T1	LAG3	1.47
P5T2	LAG3	0.98
P6N	LAG3	2.55
P6T1	LAG3	2.1
P6T2	LAG3	1.58
P6T3	LAG3	2.48
P7N	LAG3	0.85
P7T1	LAG3	0.61
P7T2	LAG3	3.36
P8N	LAG3	1.3
P8T1	LAG3	1.1
P8T2	LAG3	1.47
P8T3	LAG3	0.76
P9N	LAG3	1.34
P9T1	LAG3	1.07
P9T2	LAG3	0.5
P9T3	LAG3	1.77
P10N	LAG3	0.16
P10T1	LAG3	0.33
P10T2	LAG3	0.79
P1N	NKG7	4.27
P1T1	NKG7	7.02
P1T2	NKG7	13.02
P1T3	NKG7	20.23
P2N	NKG7	102.98
P2T1	NKG7	38.54
P2T2	NKG7	46.68
P2T3	NKG7	7.25
P3N	NKG7	71.7
P3T1	NKG7	3.25

P3T2	NKG7	8.59
P3T3	NKG7	19.6
P4N	NKG7	73.04
P4T1	NKG7	4.59
P4T2	NKG7	2.09
P4T3	NKG7	4.3
P5N	NKG7	13.49
P5T1	NKG7	22.43
P5T2	NKG7	8.16
P6N	NKG7	92.77
P6T1	NKG7	29.63
P6T2	NKG7	57.35
P6T3	NKG7	11.74
P7N	NKG7	9.59
P7T1	NKG7	2.21
P7T2	NKG7	12.01
P8N	NKG7	8.55
P8T1	NKG7	5.89
P8T2	NKG7	7.46
P8T3	NKG7	5.05
P9N	NKG7	12.88
P9T1	NKG7	2.26
P9T2	NKG7	1.18
P9T3	NKG7	34.98
P10N	NKG7	1.31
P10T1	NKG7	2.19
P10T2	NKG7	1.27
P1N	PDCD1	0.13
P1T1	PDCD1	0.26
P1T2	PDCD1	0.22
P1T3	PDCD1	0.56
P2N	PDCD1	1.36
P2T1	PDCD1	1.91
P2T2	PDCD1	0.81
P2T3	PDCD1	0.46
P3N	PDCD1	0.29
P3T1	PDCD1	0.46
P3T2	PDCD1	0.3
P3T3	PDCD1	0.4
P4N	PDCD1	0.52
P4T1	PDCD1	0.89
P4T2	PDCD1	0.33
P4T3	PDCD1	0.4
P5N	PDCD1	7.00E-02

P5T1	PDCD1	0.43
P5T2	PDCD1	0.35
P6N	PDCD1	0.33
P6T1	PDCD1	0.88
P6T2	PDCD1	0.68
P6T3	PDCD1	0.65
P7N	PDCD1	0.64
P7T1	PDCD1	0.75
P7T2	PDCD1	1.28
P8N	PDCD1	0.62
P8T1	PDCD1	0.32
P8T2	PDCD1	1
P8T3	PDCD1	0.92
P9N	PDCD1	0.24
P9T1	PDCD1	0.34
P9T2	PDCD1	0.23
P9T3	PDCD1	0.32
P10N	PDCD1	0.05
P10T1	PDCD1	0.12
P10T2	PDCD1	0.37
P1N	PRF1	1.07
P1T1	PRF1	2.88
P1T2	PRF1	6.28
P1T3	PRF1	6.41
P2N	PRF1	32.18
P2T1	PRF1	11.95
P2T2	PRF1	14.53
P2T3	PRF1	3.57
P3N	PRF1	14.72
P3T1	PRF1	1.82
P3T2	PRF1	2.02
P3T3	PRF1	5.08
P4N	PRF1	16.66
P4T1	PRF1	2.22
P4T2	PRF1	1.43
P4T3	PRF1	2.18
P5N	PRF1	6.07
P5T1	PRF1	2.75
P5T2	PRF1	4.19
P6N	PRF1	15.44
P6T1	PRF1	14.48
P6T2	PRF1	17.99
P6T3	PRF1	4.41
P7N	PRF1	4.95

P7T1	PRF1	1.51
P7T2	PRF1	3.55
P8N	PRF1	4.26
P8T1	PRF1	3.73
P8T2	PRF1	8.68
P8T3	PRF1	2.41
P9N	PRF1	5.95
P9T1	PRF1	1.01
P9T2	PRF1	1.23
P9T3	PRF1	7.02
P10N	PRF1	0.44
P10T1	PRF1	0.77
P10T2	PRF1	0.55
P1N	PSMB10	3.55
P1T1	PSMB10	7.92
P1T2	PSMB10	11.3
P1T3	PSMB10	16.93
P2N	PSMB10	119.16
P2T1	PSMB10	111.81
P2T2	PSMB10	111.44
P2T3	PSMB10	13.39
P3N	PSMB10	105.41
P3T1	PSMB10	11.05
P3T2	PSMB10	106.49
P3T3	PSMB10	119.53
P4N	PSMB10	126.58
P4T1	PSMB10	11.36
P4T2	PSMB10	11.13
P4T3	PSMB10	12.92
P5N	PSMB10	6.26
P5T1	PSMB10	62.08
P5T2	PSMB10	5.93
P6N	PSMB10	153.89
P6T1	PSMB10	57.87
P6T2	PSMB10	96.22
P6T3	PSMB10	78.64
P7N	PSMB10	14.98
P7T1	PSMB10	7.35
P7T2	PSMB10	81.91
P8N	PSMB10	14.74
P8T1	PSMB10	14.51
P8T2	PSMB10	111.13
P8T3	PSMB10	86.91
P9N	PSMB10	16.45

P9T1	PSMB10	8.98
P9T2	PSMB10	4.18
P9T3	PSMB10	115.03
P10N	PSMB10	4.23
P10T1	PSMB10	27.95
P10T2	PSMB10	7.73
P1N	STAT1	28.78
P1T1	STAT1	43.43
P1T2	STAT1	37.33
P1T3	STAT1	28.65
P2N	STAT1	74.12
P2T1	STAT1	41.84
P2T2	STAT1	64.06
P2T3	STAT1	48.1
P3N	STAT1	20.53
P3T1	STAT1	11.12
P3T2	STAT1	16.66
P3T3	STAT1	30.71
P4N	STAT1	28.21
P4T1	STAT1	12.93
P4T2	STAT1	28.29
P4T3	STAT1	22.97
P5N	STAT1	22.34
P5T1	STAT1	19.3
P5T2	STAT1	16.56
P6N	STAT1	12.7
P6T1	STAT1	39.91
P6T2	STAT1	29.51
P6T3	STAT1	23.38
P7N	STAT1	32.08
P7T1	STAT1	12.42
P7T2	STAT1	47.56
P8N	STAT1	22.04
P8T1	STAT1	17.57
P8T2	STAT1	41.59
P8T3	STAT1	21.29
P9N	STAT1	23.55
P9T1	STAT1	7.14
P9T2	STAT1	3.57
P9T3	STAT1	22.16
P10N	STAT1	4.9
P10T1	STAT1	9.37
P10T2	STAT1	25.36
P1N	TIGIT	1

P1T1	TIGIT	1.75
P1T2	TIGIT	1.71
P1T3	TIGIT	1.42
P2N	TIGIT	1.8
P2T1	TIGIT	1.95
P2T2	TIGIT	1.3
P2T3	TIGIT	1.11
P3N	TIGIT	0.93
P3T1	TIGIT	0.68
P3T2	TIGIT	0.62
P3T3	TIGIT	0.77
P4N	TIGIT	1.01
P4T1	TIGIT	0.68
P4T2	TIGIT	0.73
P4T3	TIGIT	1.61
P5N	TIGIT	1.61
P5T1	TIGIT	1.16
P5T2	TIGIT	1.56
P6N	TIGIT	0.66
P6T1	TIGIT	1.62
P6T2	TIGIT	1.31
P6T3	TIGIT	1.53
P7N	TIGIT	0.56
P7T1	TIGIT	1.02
P7T2	TIGIT	1.63
P8N	TIGIT	0.92
P8T1	TIGIT	1.12
P8T2	TIGIT	2.22
P8T3	TIGIT	2.01
P9N	TIGIT	1.46
P9T1	TIGIT	0.85
P9T2	TIGIT	0.43
P9T3	TIGIT	0.71
P10N	TIGIT	0.12
P10T1	TIGIT	0.49
P10T2	TIGIT	1.19
P1N	GZMA	2.69
P1T1	GZMA	5.07
P1T2	GZMA	5.42
P1T3	GZMA	3.85
P2N	GZMA	24.96
P2T1	GZMA	20.61
P2T2	GZMA	15.98
P2T3	GZMA	2.76

P3N	GZMA	14.27
P3T1	GZMA	0.81
P3T2	GZMA	4.04
P3T3	GZMA	7.06
P4N	GZMA	13.26
P4T1	GZMA	0.53
P4T2	GZMA	0.85
P4T3	GZMA	2.25
P5N	GZMA	6.47
P5T1	GZMA	9.78
P5T2	GZMA	3.18
P6N	GZMA	11.16
P6T1	GZMA	13.23
P6T2	GZMA	15.86
P6T3	GZMA	7.31
P7N	GZMA	0.94
P7T1	GZMA	1.03
P7T2	GZMA	8.79
P8N	GZMA	1.26
P8T1	GZMA	0.78
P8T2	GZMA	5.56
P8T3	GZMA	4.77
P9N	GZMA	1.43
P9T1	GZMA	1.09
P9T2	GZMA	0.69
P9T3	GZMA	8.92
P10N	GZMA	0.43
P10T1	GZMA	1.56
P10T2	GZMA	0.49
P1N	IFI16	31.45
P1T1	IFI16	31.72
P1T2	IFI16	42.1
P1T3	IFI16	23.73
P2N	IFI16	182.13
P2T1	IFI16	150.1
P2T2	IFI16	92.9
P2T3	IFI16	21
P3N	IFI16	52.16
P3T1	IFI16	3.98
P3T2	IFI16	95.79
P3T3	IFI16	79.18
P4N	IFI16	61.41
P4T1	IFI16	7.15
P4T2	IFI16	10.3

P4T3	IFI16	18.63
P5N	IFI16	13.65
P5T1	IFI16	53.26
P5T2	IFI16	10.9
P6N	IFI16	54.18
P6T1	IFI16	62.09
P6T2	IFI16	59.06
P6T3	IFI16	36.25
P7N	IFI16	8.96
P7T1	IFI16	8.15
P7T2	IFI16	62.16
P8N	IFI16	4.88
P8T1	IFI16	6.12
P8T2	IFI16	66.02
P8T3	IFI16	30.6
P9N	IFI16	13.23
P9T1	IFI16	4.68
P9T2	IFI16	3.29
P9T3	IFI16	59.3
P10N	IFI16	5.89
P10T1	IFI16	18.46
P10T2	IFI16	6.73
P1N	IFI27	26.16
P1T1	IFI27	39.6
P1T2	IFI27	67.1
P1T3	IFI27	47.09
P2N	IFI27	1596.77
P2T1	IFI27	1213.25
P2T2	IFI27	1377.66
P2T3	IFI27	56.56
P3N	IFI27	797.27
P3T1	IFI27	14.51
P3T2	IFI27	1085.49
P3T3	IFI27	2333.56
P4N	IFI27	834.19
P4T1	IFI27	39.56
P4T2	IFI27	52.17
P4T3	IFI27	34.02
P5N	IFI27	57.23
P5T1	IFI27	352.64
P5T2	IFI27	52.2
P6N	IFI27	708.96
P6T1	IFI27	256.59
P6T2	IFI27	584.29

P6T3	IFI27	136.33
P7N	IFI27	93.35
P7T1	IFI27	12.71
P7T2	IFI27	664.06
P8N	IFI27	34.98
P8T1	IFI27	55.28
P8T2	IFI27	422.26
P8T3	IFI27	382.41
P9N	IFI27	76.32
P9T1	IFI27	13.41
P9T2	IFI27	16.86
P9T3	IFI27	1052.01
P10N	IFI27	32.13
P10T1	IFI27	53.58
P10T2	IFI27	40.5
P1N	IFI44	16.8
P1T1	IFI44	21.64
P1T2	IFI44	30.38
P1T3	IFI44	17.14
P2N	IFI44	121.5
P2T1	IFI44	103.11
P2T2	IFI44	99.63
P2T3	IFI44	44.03
P3N	IFI44	29.36
P3T1	IFI44	1.8
P3T2	IFI44	44.57
P3T3	IFI44	59.33
P4N	IFI44	32.89
P4T1	IFI44	2.57
P4T2	IFI44	83.57
P4T3	IFI44	14.86
P5N	IFI44	14.57
P5T1	IFI44	17.81
P5T2	IFI44	10.84
P6N	IFI44	20.16
P6T1	IFI44	21.58
P6T2	IFI44	26.08
P6T3	IFI44	18.08
P7N	IFI44	18.88
P7T1	IFI44	8.39
P7T2	IFI44	47.35
P8N	IFI44	10.21
P8T1	IFI44	10.22
P8T2	IFI44	38.1

P8T3	IFI44	20.49
P9N	IFI44	16.17
P9T1	IFI44	3.46
P9T2	IFI44	1.57
P9T3	IFI44	29.79
P10N	IFI44	2.33
P10T1	IFI44	8.96
P10T2	IFI44	28.29
P1N	IFI44L	8.6
P1T1	IFI44L	11.9
P1T2	IFI44L	13.48
P1T3	IFI44L	6.65
P2N	IFI44L	40.43
P2T1	IFI44L	27.36
P2T2	IFI44L	38.22
P2T3	IFI44L	36.85
P3N	IFI44L	4.51
P3T1	IFI44L	0.78
P3T2	IFI44L	9.25
P3T3	IFI44L	14.71
P4N	IFI44L	6.42
P4T1	IFI44L	0.98
P4T2	IFI44L	64.1
P4T3	IFI44L	7.36
P5N	IFI44L	7.56
P5T1	IFI44L	4.25
P5T2	IFI44L	5.9
P6N	IFI44L	2.22
P6T1	IFI44L	4.59
P6T2	IFI44L	3.23
P6T3	IFI44L	1.84
P7N	IFI44L	11.73
P7T1	IFI44L	4.36
P7T2	IFI44L	22.35
P8N	IFI44L	3.86
P8T1	IFI44L	4.67
P8T2	IFI44L	10.1
P8T3	IFI44L	1.65
P9N	IFI44L	5.11
P9T1	IFI44L	1.26
P9T2	IFI44L	1.01
P9T3	IFI44L	6.9
P10N	IFI44L	1.19
P10T1	IFI44L	2.42

P10T2	IFI44L	23.86
P1N	MX1	8.09
P1T1	MX1	16.44
P1T2	MX1	17.54
P1T3	MX1	17.11
P2N	MX1	181
P2T1	MX1	129.36
P2T2	MX1	148.03
P2T3	MX1	55.32
P3N	MX1	35.16
P3T1	MX1	7.77
P3T2	MX1	61.41
P3T3	MX1	94.99
P4N	MX1	59.76
P4T1	MX1	16.52
P4T2	MX1	39.61
P4T3	MX1	21.21
P5N	MX1	12.31
P5T1	MX1	22.31
P5T2	MX1	14.67
P6N	MX1	31.34
P6T1	MX1	36.34
P6T2	MX1	34.92
P6T3	MX1	23.43
P7N	MX1	27.94
P7T1	MX1	10.83
P7T2	MX1	42.97
P8N	MX1	16.24
P8T1	MX1	22.32
P8T2	MX1	38.91
P8T3	MX1	32.92
P9N	MX1	14.6
P9T1	MX1	7.79
P9T2	MX1	10.52
P9T3	MX1	41.87
P10N	MX1	3.49
P10T1	MX1	7.08
P10T2	MX1	25.95
P1N	OASL	1.22
P1T1	OASL	2.29
P1T2	OASL	0.9
P1T3	OASL	1.23
P2N	OASL	22.97
P2T1	OASL	15.23

P2T2	OASL	18.73
P2T3	OASL	2.73
P3N	OASL	15.1
P3T1	OASL	0.64
P3T2	OASL	10.92
P3T3	OASL	15.08
P4N	OASL	11.11
P4T1	OASL	0.82
P4T2	OASL	1.03
P4T3	OASL	0.92
P5N	OASL	2.83
P5T1	OASL	3.22
P5T2	OASL	1.65
P6N	OASL	11.66
P6T1	OASL	9.07
P6T2	OASL	7.87
P6T3	OASL	3.02
P7N	OASL	1.95
P7T1	OASL	0.96
P7T2	OASL	3.35
P8N	OASL	4.02
P8T1	OASL	1.96
P8T2	OASL	6.27
P8T3	OASL	4.18
P9N	OASL	1.58
P9T1	OASL	0.43
P9T2	OASL	0.41
P9T3	OASL	9.82
P10N	OASL	0.31
P10T1	OASL	1.05
P10T2	OASL	1.71
P1N	GZMK	2.65
P1T1	GZMK	4.76
P1T2	GZMK	1.5
P1T3	GZMK	2.27
P2N	GZMK	9.78
P2T1	GZMK	18.61
P2T2	GZMK	9.47
P2T3	GZMK	3.43
P3N	GZMK	2.95
P3T1	GZMK	0.19
P3T2	GZMK	2.2
P3T3	GZMK	1.92
P4N	GZMK	2.94

P4T1	GZMK	0.23
P4T2	GZMK	0.6
P4T3	GZMK	2.56
P5N	GZMK	1.41
P5T1	GZMK	5.17
P5T2	GZMK	1.18
P6N	GZMK	1.45
P6T1	GZMK	4.15
P6T2	GZMK	3.03
P6T3	GZMK	2.96
P7N	GZMK	0.29
P7T1	GZMK	0.98
P7T2	GZMK	9.45
P8N	GZMK	0.76
P8T1	GZMK	0.15
P8T2	GZMK	1.82
P8T3	GZMK	3.36
P9N	GZMK	0.35
P9T1	GZMK	0.53
P9T2	GZMK	0.43
P9T3	GZMK	2.09
P10N	GZMK	0.41
P10T1	GZMK	1.62
P10T2	GZMK	0.49
P1N	MS4A1	0.68
P1T1	MS4A1	0.94
P1T2	MS4A1	1.21
P1T3	MS4A1	3.54
P2N	MS4A1	0.66
P2T1	MS4A1	1
P2T2	MS4A1	0.52
P2T3	MS4A1	0.91
P3N	MS4A1	0.39
P3T1	MS4A1	0.67
P3T2	MS4A1	0.36
P3T3	MS4A1	1
P4N	MS4A1	0.21
P4T1	MS4A1	0.05
P4T2	MS4A1	0.35
P4T3	MS4A1	3
P5N	MS4A1	0.63
P5T1	MS4A1	0.7
P5T2	MS4A1	0.71
P6N	MS4A1	0.19

P6T1	MS4A1	6.18
P6T2	MS4A1	1.07
P6T3	MS4A1	6.02
P7N	MS4A1	0.22
P7T1	MS4A1	3.94
P7T2	MS4A1	2.4
P8N	MS4A1	0.31
P8T1	MS4A1	0.22
P8T2	MS4A1	8.66
P8T3	MS4A1	5.89
P9N	MS4A1	0.61
P9T1	MS4A1	8.44
P9T2	MS4A1	0.38
P9T3	MS4A1	0.43
P10N	MS4A1	0.02
P10T1	MS4A1	2.75
P10T2	MS4A1	4.97
P1N	TOX	1.95
P1T1	TOX	4.81
P1T2	TOX	2.91
P1T3	TOX	2.39
P2N	TOX	1.78
P2T1	TOX	2.81
P2T2	TOX	1.98
P2T3	TOX	4.48
P3N	TOX	1.33
P3T1	TOX	1.31
P3T2	TOX	4.8
P3T3	TOX	5.3
P4N	TOX	1.97
P4T1	TOX	0.19
P4T2	TOX	4.08
P4T3	TOX	3.36
P5N	TOX	1.42
P5T1	TOX	2.93
P5T2	TOX	1.03
P6N	TOX	0.98
P6T1	TOX	4.95
P6T2	TOX	3.31
P6T3	TOX	3.65
P7N	TOX	0.74
P7T1	TOX	1.92
P7T2	TOX	3.93
P8N	TOX	0.74

P8T1	TOX	0.64
P8T2	TOX	0.83
P8T3	TOX	2.63
P9N	TOX	1.98
P9T1	TOX	0.62
P9T2	TOX	0.24
P9T3	TOX	2.31
P10N	TOX	0.12
P10T1	TOX	1.37
P10T2	TOX	0.47

Supplementary Table 4 Immune signature estimation

Sample	Cytolytic signature	IFN-gamma signature	T-cell exhaustion signature	T-cell naive signature	CD8+ effector T signature
P1N	1.7076299	10.290123	0.5854406	4.2913901	0.4608356
P1T1	3.8316054	15.185289	1.2577368	10.323685	1.2976423
P1T2	5.8442023	16.263185	1.2295492	10.276116	1.4942035
P1T3	4.9780719	11.808486	1.6325044	13.611683	1.7986626
P2N	28.35109	134.59255	2.4861697	22.325462	4.8937535
P2T1	15.703987	100.22282	2.1474491	16.341839	3.6737707
P2T2	15.247774	105.16367	1.7282479	14.448647	2.9342551
P2T3	3.1490634	25.764458	1.1604974	10.299356	0.8720015
P3N	14.503255	37.837156	1.3079303	9.3642147	2.840178
P3T1	1.2249898	2.7354137	0.6488617	3.8611199	0.6177708
P3T2	2.8673158	55.370621	0.7863803	5.2857181	0.6748033
P3T3	5.9988582	78.35377	1.1723227	8.9881714	1.8556101
P4N	14.87316	43.946992	1.5039172	10.279419	4.4441995
P4T1	1.0973605	4.6360433	0.7830185	2.1437049	0.6688557
P4T2	1.1128342	22.17123	0.8787886	2.9849097	0.6792985
P4T3	2.2247247	10.541096	1.1319277	6.5614628	1.5288164
P5N	6.2768145	12.022334	0.8038922	5.2084628	1.3931441
P5T1	5.198115	21.644128	1.1071891	3.3074907	2.0294623
P5T2	3.6603279	9.8078008	1.0509605	4.3782906	1.1925702
P6N	13.136838	29.296182	1.2168437	8.1147308	2.3376587
P6T1	13.850906	28.378635	1.9055322	18.547947	2.7452049
P6T2	16.901479	30.487686	1.6559321	13.920299	3.7373772
P6T3	5.6880928	15.077995	1.4580334	12.87143	1.7112206
P7N	2.1707142	14.720251	0.7603213	4.2532167	0.7736219
P7T1	1.2572987	5.8495596	0.7793611	5.546821	0.738223
P7T2	5.5971421	42.991307	1.2867848	8.5040906	1.0748884
P8N	2.3287121	8.7316338	0.7828169	4.3639615	0.5317205
P8T1	1.718895	9.4536006	0.8644645	3.0564894	0.4016708
P8T2	6.957248	37.142889	1.4693196	14.593544	1.590526
P8T3	3.4011175	19.498982	1.1204088	7.1984844	1.1752911
P9N	2.9295733	11.172401	1.0148908	7.4194129	1.0583171
P9T1	1.059245	3.1364207	0.6143183	4.1567213	0.9033064
P9T2	0.9316652	2.7107782	0.2920895	1.1696889	0.2069764
P9T3	7.9232506	41.74147	0.9167403	7.2700287	1.8589239
P10N	0.4449719	2.9012523	0.0984285	0.6013866	0.1463704
P10T1	1.1066165	7.3844289	0.2993271	2.2689075	0.357715
P10T2	0.5291503	14.210707	0.7266401	4.2229733	0.3431644

Supplementary Table 5 Cell density estimated by multiplex immunofluorescence

label	Mutation	Pathology	Treg Cell Density (n/mm2)	B Cell Density (n/mm2)	NK cell Density (n/mm2)
P11	KRAS_MT	Normal	3.5877018	17.041583	0.8969254
P11	KRAS_MT	MIA+IA	93.206017	342.77739	0.3931022
P11	KRAS_MT	AIS	25.420611	137.35647	0.6131975
P12	KRAS_MT	MIA+IA	190.60194	23.498869	0
P12	KRAS_MT	AIS	115.71439	32.051359	1.7074088
P13	KRAS_MT	AIS	67.609538	102.70371	121.56823
P14	Others	MIA+IA	181.17432	75.748147	0.8091635
P15	EGFR_MT	AIS	21.692643	12.886718	11.812825
P16	EGFR_MT	Normal	8.349687	4.0401712	12.384032
P16	EGFR_MT	MIA+IA	109.36321	156.63184	0.5386895
P16	EGFR_MT	AIS	24.393157	12.914024	6.5949824
P17	EGFR_MT	Normal	7.4329533	5.1261747	3.3274529
P17	EGFR_MT	AIS	55.777088	13.528911	1.1867466
P17	EGFR_MT	MIA+IA	62.389742	69.417552	0.5126175
P18	Others	AIS	27.812227	5.7390309	38.797731
P18	Others	MIA+IA	44.712019	13.169148	4.4146392

Supplementary Table 6 Differentially expressed genes between nodule and normal groups

Genes	BaseMean	Log2FoldChange	LfcSE	Stat	Pvalue
FDCSP	32.483176	23.088087	1.4948997	15.444572	8.21E-54
HS6ST2	270.011	4.553756	0.5221598	8.7210003	2.76E-18
ARHGAP40	292.2089	4.4043941	0.5236763	8.4105279	4.08E-17
TMEM59L	141.66618	4.5965539	0.5571158	8.250626	1.58E-16
EPHA10	126.65848	3.8138657	0.4754504	8.0215857	1.04E-15
MUC21	392.64375	4.3475112	0.5467169	7.9520336	1.83E-15
ABCC3	4996.3514	3.0790732	0.3929729	7.8353331	4.68E-15
ADM2	127.08037	2.8070032	0.3655829	7.6781576	1.61E-14
GFRA3	160.50393	4.442061	0.5812062	7.642832	2.12E-14
GLB1L3	366.33011	5.7300606	0.7717558	7.4247072	1.13E-13
HABP2	429.54428	5.0099612	0.6765493	7.4051677	1.31E-13
GDF15	3747.7163	3.5921417	0.4858684	7.3932394	1.43E-13
FEZF1-AS1	63.698415	5.5582065	0.7609578	7.3042246	2.79E-13
XDH	181.8871	4.2687355	0.5883345	7.2556264	4.00E-13
NELL1	264.40524	7.3771317	1.0393333	7.0979463	1.27E-12
CDH3	470.82655	2.8304239	0.4040352	7.0053897	2.46E-12
CXCL13	158.90574	6.2985216	0.9119504	6.9066495	4.96E-12
CRLF1	583.46791	3.7296486	0.5455819	6.8360934	8.14E-12
CEACAM5	1561.4867	4.7732213	0.7070219	6.7511649	1.47E-11
ETV4	427.27906	2.6626111	0.3962304	6.7198559	1.82E-11
MNX1	52.374383	4.2164785	0.6301301	6.6914413	2.21E-11
SPINK1	750.93416	7.0545003	1.0628403	6.637404	3.19E-11
UNC5CL	127.5208	2.3846474	0.3590396	6.6417399	3.10E-11
IL2RA	64.03622	1.7966416	0.2709789	6.630191	3.35E-11
CRYM	535.62566	3.5137084	0.5330521	6.5916795	4.35E-11
KCNK5	972.34056	1.9862475	0.3053829	6.5041213	7.81E-11
CR2	79.016584	5.2916801	0.8180516	6.4686388	9.89E-11
RASAL1	64.307311	3.2866176	0.5078282	6.4719091	9.68E-11
CA10	41.217439	4.9786706	0.7732693	6.4384688	1.21E-10
PROM2	767.44074	2.3891318	0.373583	6.3951841	1.60E-10
COMP	481.97354	4.4658454	0.7026412	6.3557977	2.07E-10
ABCA4	185.30842	3.5795698	0.5655538	6.3293178	2.46E-10
GYLTL1B	218.09397	1.6921328	0.2676166	6.3229747	2.57E-10
PVT1	158.826	2.6115657	0.417584	6.2539882	4.00E-10
RTN4RL2	292.61696	2.1502864	0.343857	6.2534315	4.02E-10
PVRL4	514.46514	1.8907232	0.3031796	6.2363142	4.48E-10
CD1A	156.16482	4.1589709	0.6676446	6.2293185	4.68E-10
METTL7B	118.9028	2.616785	0.4207134	6.2198763	4.98E-10

ST6GALNAC1	400.21352	2.3684761	0.3811271	6.2143997	5.15E-10
B3GNT3	129.31434	3.2692009	0.5267414	6.2064629	5.42E-10
FUT2	133.30526	2.0392286	0.3295726	6.1874942	6.11E-10
AFAP1-AS1	901.43475	4.6254491	0.7554703	6.1226089	9.21E-10
TOX3	308.27657	3.1421503	0.514592	6.1061004	1.02E-09
MMP7	797.57893	3.6292789	0.5963246	6.08608	1.16E-09
CILP2	50.009454	3.3074756	0.5518245	5.9937093	2.05E-09
C2CD4A	84.190742	2.9405977	0.4996375	5.8854627	3.97E-09
GCNT3	53.864299	3.5614563	0.608391	5.8538938	4.80E-09
PLEKHN1	119.0856	3.0051458	0.5140118	5.846453	5.02E-09
ANKRD34B	22.54705	4.8741291	0.8423292	5.7864892	7.19E-09
CCDC64	252.82953	1.8360115	0.3174469	5.7836813	7.31E-09
CD207	269.04868	3.2057359	0.5562915	5.7626908	8.28E-09
MMP11	209.52637	3.3564886	0.5868051	5.7199377	1.07E-08
PYCR1	657.30633	2.1358252	0.3737618	5.7144019	1.10E-08
SPP1	436.69404	3.9208434	0.6872788	5.7048805	1.16E-08
MYBPC2	19.063791	3.869616	0.6786704	5.7017604	1.19E-08
ARHGEF19	698.77527	1.9187058	0.3378348	5.679421	1.35E-08
SALL4	65.288994	3.2531575	0.5724091	5.683274	1.32E-08
TMPRSS4	351.21952	3.2092529	0.5648919	5.6811811	1.34E-08
HOXD1	465.55559	2.8626636	0.5056419	5.6614451	1.50E-08
MUC3A	501.08745	3.7150393	0.656323	5.6603827	1.51E-08
STEAP3	1253.7447	1.7060368	0.3019436	5.6501843	1.60E-08
TMEM105	64.08478	2.7829159	0.494936	5.6227788	1.88E-08
CST1	145.23806	5.9576155	1.063582	5.6014635	2.13E-08
MANEAL	199.51278	1.6553581	0.2960565	5.5913579	2.25E-08
CDKN2A	316.38055	2.1990749	0.3950678	5.5663229	2.60E-08
SLC6A3	157.71284	3.8865251	0.6990573	5.5596661	2.70E-08
BLK	80.209427	2.7759188	0.5024777	5.5244615	3.30E-08
GLS2	173.70792	1.7249638	0.3126714	5.5168588	3.45E-08
DPP4	1073.8417	2.0319028	0.3698482	5.4938829	3.93E-08
CCL22	270.13471	1.8077727	0.3303273	5.4726717	4.43E-08
EPN3	112.75207	2.3129841	0.4235245	5.4612758	4.73E-08
AOC1	54.549514	3.1526015	0.5777899	5.456311	4.86E-08
ANKRD22	112.35964	3.2562534	0.5975264	5.449556	5.05E-08
CACNB1	951.89349	1.5496402	0.2844336	5.4481624	5.09E-08
SUSD4	248.55347	1.9202423	0.3535502	5.4313138	5.59E-08
CTSE	2123.6995	2.7340714	0.5051336	5.4125713	6.21E-08
EEF1A2	231.56078	4.9475522	0.9153206	5.4052669	6.47E-08
RAP1GAP	3025.7923	2.2759142	0.4211855	5.4035914	6.53E-08
GJB2	49.680991	2.7153636	0.5042565	5.3848859	7.25E-08
KLHDC8A	31.05737	2.6833793	0.500188	5.3647411	8.11E-08
MESP1	214.09942	2.5952037	0.4839132	5.3629534	8.19E-08
AQP3	7332.5235	1.8674276	0.3499638	5.3360597	9.50E-08

GGT5	1564.3989	2.1547789	0.4043386	5.3291445	9.87E-08
BAAT	76.547789	3.2779927	0.6157038	5.3239766	1.02E-07
FCRLA	33.662568	3.2727883	0.614675	5.3244205	1.01E-07
GPT2	292.49689	1.7229231	0.3248448	5.3038343	1.13E-07
MESP2	15.898163	3.542687	0.6678355	5.3047299	1.13E-07
SYT16	13.337838	6.3360304	1.1947181	5.3033685	1.14E-07
MROH6	741.30775	2.1344211	0.4026899	5.3004085	1.16E-07
SMPDL3B	763.68314	2.2400406	0.4228603	5.2973532	1.17E-07
VPREB3	26.348237	3.633258	0.6900746	5.2650222	1.40E-07
STMN2	30.591316	3.6574969	0.69821	5.238391	1.62E-07
MS4A1	168.56033	2.8992789	0.5541806	5.2316498	1.68E-07
FUT3	255.234	1.7981	0.3446926	5.2165318	1.82E-07
KIF1A	44.499993	3.3983356	0.6514311	5.2167227	1.82E-07
TMEM88B	29.489627	4.4311585	0.8491562	5.2183079	1.81E-07
CLIC6	2328.8274	2.8342228	0.5473261	5.1783077	2.24E-07
TUBB3	110.37443	2.810872	0.5448792	5.1587063	2.49E-07
FCGBP	2704.1544	2.1392292	0.4156657	5.1465132	2.65E-07
CRABP2	698.70333	4.3222142	0.8406277	5.1416506	2.72E-07
C1orf170	66.453942	2.2325745	0.4350927	5.1312616	2.88E-07
KCNK1	315.87657	1.6497674	0.3215516	5.1306455	2.89E-07
KCNMB4	338.03469	-1.622238	0.3169539	-5.118214	3.08E-07
hsa-mir-7162	45.277925	3.8841916	0.7591218	5.1166906	3.11E-07
ASPHD1	98.930337	2.3146593	0.4536066	5.1027899	3.35E-07
ATP10B	83.705188	4.290094	0.8410039	5.1011585	3.38E-07
LRAT	56.290166	2.0832544	0.4089239	5.0944797	3.50E-07
CD27	116.04598	1.5325967	0.3031363	5.0558001	4.29E-07
PAX5	71.676246	3.4729319	0.6867177	5.0572919	4.25E-07
FEZF1	13.362776	4.4235718	0.8794545	5.0299041	4.91E-07
SEZ6L2	818.34953	1.9166668	0.3812059	5.0279044	4.96E-07
SBK1	402.00781	1.8330373	0.3648018	5.0247492	5.04E-07
SPTBN2	636.8843	2.051629	0.4111578	4.989882	6.04E-07
TMEM63C	70.369594	2.8984444	0.5812312	4.9867326	6.14E-07
CPNE7	88.507116	2.9318632	0.5881322	4.9850411	6.19E-07
KRT16P1	431.24492	4.1459662	0.8324635	4.9803581	6.35E-07
IGSF9	172.21673	2.1633283	0.4346585	4.9770755	6.46E-07
RHBDL2	63.300415	1.8662754	0.3756106	4.9686439	6.74E-07
RBBP8NL	93.204191	1.7377536	0.350298	4.9607865	7.02E-07
CBLC	117.97994	2.0692334	0.4177078	4.9537822	7.28E-07
GLP2R	31.587809	-2.412963	0.487893	-4.945682	7.59E-07
CAPN8	2461.5619	1.9802965	0.4028757	4.9154032	8.86E-07
CIT	1877.4875	1.9919097	0.4056291	4.9106679	9.08E-07
COL9A2	369.8387	1.5390946	0.3133745	4.9113596	9.04E-07
KRT15	585.15521	2.6852675	0.5465981	4.9126907	8.98E-07
STRA6	43.18206	3.1366052	0.6382794	4.9141571	8.92E-07

HKDC1	223.31496	2.1518729	0.4387026	4.9050832	9.34E-07
PABPC1L	895.48782	1.5246251	0.3116898	4.8914819	1.00E-06
SCTR	1449.3808	2.1480559	0.4399693	4.8822856	1.05E-06
P2RY6	122.36299	1.6481242	0.3393464	4.856761	1.19E-06
SGPP2	621.16821	2.0330228	0.418608	4.8566269	1.19E-06
HLA-DQB2	694.86168	2.605471	0.536982	4.8520644	1.22E-06
COL17A1	182.32433	2.7101383	0.5627162	4.8161724	1.46E-06
MYH2	45.117438	-2.693144	0.5601666	-4.807755	1.53E-06
MMP13	17.176892	4.3500057	0.9059657	4.8015123	1.57E-06
CLDN6	38.924554	5.3049201	1.1078223	4.7886019	1.68E-06
CPA6	45.248892	3.0886343	0.6494397	4.7558449	1.98E-06
GYG2	79.165708	1.7455311	0.3679894	4.7434278	2.10E-06
C4B	4109.3014	1.7350092	0.3677834	4.7174753	2.39E-06
LAD1	1339.7404	1.6650402	0.3544203	4.6979254	2.63E-06
CD79A	270.84303	2.3659577	0.5047355	4.6875194	2.77E-06
FAM83A	69.175759	3.1582395	0.6753282	4.6765995	2.92E-06
CD19	39.617329	2.7546264	0.5893645	4.6738923	2.96E-06
CCL19	226.5957	2.2645146	0.4848803	4.670255	3.01E-06
LINC00702	118.56622	-1.589049	0.3401663	-4.671387	2.99E-06
MFSD6L	74.046092	1.9221659	0.4122856	4.6622188	3.13E-06
MST1L	518.31103	1.5988955	0.3430559	4.6607433	3.15E-06
FAM83H-AS1	489.48115	1.8794002	0.4040998	4.6508317	3.31E-06
GJB1	184.22627	1.9410798	0.4172845	4.651694	3.29E-06
SLC7A10	17.218057	3.1540858	0.6779636	4.6522936	3.28E-06
OSM	178.76556	2.6035462	0.5610867	4.6401848	3.48E-06
LEMD1	19.112135	3.4837545	0.7536373	4.6225876	3.79E-06
BTNL8	34.012775	-1.846488	0.3998591	-4.617847	3.88E-06
GJB6	10.893377	5.1765147	1.1222171	4.6127569	3.97E-06
DERL3	280.56432	1.7102859	0.3717616	4.6004903	4.21E-06
B3GNT6	63.171816	3.8310823	0.8332998	4.5974836	4.28E-06
RHPN1	1241.5482	1.7361917	0.3782396	4.59019	4.43E-06
KISS1R	7.6220949	3.6027642	0.7877077	4.5737322	4.79E-06
OTX1	25.873704	2.5514142	0.5593775	4.5611674	5.09E-06
CD24P4	53.783406	2.4822126	0.544439	4.5592116	5.13E-06
CYP27C1	13.207888	2.9466572	0.6482946	4.5452439	5.49E-06
SYT12	26.78048	2.7171171	0.5978926	4.5444902	5.51E-06
THBS4	109.00378	2.1027445	0.4631171	4.5404164	5.61E-06
C6orf141	53.419571	1.8399324	0.4059097	4.5328615	5.82E-06
FAM46B	477.9526	-1.51597	0.3350635	-4.524427	6.06E-06
CDCA7	163.25173	2.4989208	0.5532512	4.5167927	6.28E-06
IL13RA2	31.230232	3.1147154	0.6903096	4.5120559	6.42E-06
CLEC5A	269.62977	1.9954477	0.4433984	4.5003489	6.78E-06
SPDEF	204.31832	1.8901023	0.4207462	4.4922621	7.05E-06
TMEM132E	36.516124	2.4157103	0.5386289	4.4849252	7.29E-06

TNFRSF13B	39.648525	2.3945256	0.5339471	4.4845745	7.31E-06
BCL2L15	53.139586	1.8312083	0.408626	4.4813799	7.42E-06
PODXL2	513.14592	1.9107653	0.4266481	4.4785509	7.52E-06
PIGR	25756.967	1.8382407	0.4118885	4.4629571	8.08E-06
IRF4	310.58614	1.5279403	0.3424935	4.4612239	8.15E-06
CLDN1	714.14348	1.8008803	0.4042332	4.4550533	8.39E-06
KRT80	224.46349	1.7684654	0.3968848	4.4558657	8.36E-06
FAM107A	1379.1449	-1.877165	0.4236662	-4.430763	9.39E-06
EN2	15.772918	4.3218532	0.9769448	4.4238456	9.70E-06
ADAM8	1228.0341	1.9629075	0.4445597	4.4153964	1.01E-05
ALOX15B	1770.9332	1.8298544	0.4146573	4.4129314	1.02E-05
PLA2G2C	6.218814	3.1273256	0.7100726	4.4042337	1.06E-05
CTHRC1	172.48017	1.7830621	0.4050402	4.4021854	1.07E-05
FADS6	12.68844	6.2762996	1.4259979	4.4013385	1.08E-05
LY6D	15.574603	5.2721818	1.1984822	4.399049	1.09E-05
TNFRSF17	27.909179	1.781631	0.4055622	4.3929913	1.12E-05
STK32A	466.28611	1.8310595	0.4192704	4.3672523	1.26E-05
VSIG1	34.66214	2.6295438	0.6020446	4.3676894	1.26E-05
HLA-DOB	178.61077	1.8325369	0.4199964	4.3632209	1.28E-05
CYMP	4.5653889	4.26677	0.9795537	4.3558305	1.33E-05
MNX1-AS1	8.1949181	3.6355537	0.8357796	4.3498952	1.36E-05
XAGE1C	27.951934	4.4329289	1.0193311	4.3488607	1.37E-05
SRCIN1	299.30507	1.9943975	0.4587243	4.3477043	1.38E-05
CP	817.23165	2.5906143	0.5967864	4.3409405	1.42E-05
AZU1	218.00058	2.912722	0.6714265	4.3381098	1.44E-05
POM121L9P	78.042074	1.5716604	0.3625924	4.33451	1.46E-05
DUX4	68.376864	3.6706197	0.8504518	4.3160822	1.59E-05
SIX4	178.51885	2.0185755	0.467819	4.3148644	1.60E-05
DPP10	46.046326	2.6506173	0.6155899	4.3058166	1.66E-05
OVOL1	52.541871	2.1433653	0.4977348	4.3062395	1.66E-05
PRX	1726.8567	-1.672693	0.3886661	-4.303676	1.68E-05
CELF5	16.992034	3.1480358	0.7335921	4.2912615	1.78E-05
C1orf233	569.05927	1.6442124	0.3835894	4.2863858	1.82E-05
MUC1	16594.279	1.6403625	0.38282	4.2849445	1.83E-05
IGL-@-ext	5553.8572	1.520358	0.3555466	4.2761148	1.90E-05
IGL@-ext	5553.8572	1.520358	0.3555466	4.2761148	1.90E-05
MALL	7700.8197	1.5002063	0.3516468	4.2662308	1.99E-05
GPR37	25.661534	2.1651521	0.5079179	4.2627997	2.02E-05
IL37	13.092399	3.7079051	0.8700124	4.2618993	2.03E-05
RGS1	1080.6898	2.0127766	0.4727834	4.2572913	2.07E-05
ELANE	202.34478	3.0187362	0.7095102	4.254676	2.09E-05
RHBDL1	139.14468	2.4960505	0.5870588	4.2517898	2.12E-05
KIF12	344.85595	1.8178001	0.4283833	4.2433962	2.20E-05
PPP1R1B	1396.3072	1.6415308	0.3869378	4.2423639	2.21E-05

DNAJC22	16.170697	2.2328814	0.5283875	4.2258407	2.38E-05
S100B	149.36714	2.4117251	0.5706714	4.2261188	2.38E-05
MTNR1A	3.867925	4.782592	1.1323165	4.2237238	2.40E-05
SLC15A1	22.366111	3.1245593	0.7437644	4.2010066	2.66E-05
DMRTA2	91.238796	4.2525397	1.0138295	4.1945313	2.73E-05
IL22RA2	6.6269541	4.6541937	1.1096669	4.1942258	2.74E-05
CHRDL2	46.994471	2.0788818	0.4965914	4.1863027	2.84E-05
FGF11	56.164763	1.6393361	0.3930222	4.1711028	3.03E-05
CD1E	84.930596	1.7372906	0.4179259	4.1569344	3.23E-05
KIAA1324	1460.8229	2.1375924	0.5143051	4.1562732	3.23E-05
PON1	46.283787	2.0183456	0.4861585	4.1516206	3.30E-05
LGSN	93.875954	3.3728779	0.8154455	4.1362397	3.53E-05
SLC2A5	35.949667	1.7742133	0.4292041	4.1337287	3.57E-05
FCRL5	83.636907	2.0256311	0.490352	4.1309732	3.61E-05
CYP24A1	57.74224	3.5885004	0.8700943	4.124266	3.72E-05
SLC26A9	1534.2732	1.9817055	0.4804803	4.1244259	3.72E-05
SLC28A3	85.157631	1.9595274	0.47578	4.1185573	3.81E-05
NPTX1	154.88846	3.0322026	0.7391154	4.1024753	4.09E-05
ARSE	191.14062	2.1062814	0.5139183	4.0984752	4.16E-05
CYP21A1P	32.704756	2.3303872	0.5698727	4.0893121	4.33E-05
IGH-@-ext	22831.356	1.8631512	0.4554682	4.0906288	4.30E-05
IGH@-ext	22831.356	1.8631512	0.4554682	4.0906288	4.30E-05
CYP27B1	48.968649	1.7886397	0.4379933	4.0837149	4.43E-05
EFHC2	73.968861	1.9737411	0.4840781	4.0773199	4.56E-05
TBXT	9.4077913	6.1318177	1.5075959	4.0672819	4.76E-05
TMEM184A	788.28567	1.6267408	0.3999269	4.067595	4.75E-05
CHI3L1	2764.4768	2.8664341	0.7050719	4.0654495	4.79E-05
AKR1CL1	2.843638	-2.597744	0.6406077	-4.055124	5.01E-05
GSG1L	10.147253	-1.978518	0.4880144	-4.05422	5.03E-05
SPIB	60.509866	1.9309089	0.4763198	4.053808	5.04E-05
CLDN2	320.0522	2.8422035	0.7017332	4.0502624	5.12E-05
ATP6V0A4	77.350391	2.6541694	0.6555784	4.0485918	5.15E-05
KIF26B	735.65916	2.2348095	0.5521346	4.0475807	5.17E-05
SYT7	263.66501	1.5433115	0.3814186	4.0462409	5.20E-05
CSPG5	104.505	1.7275274	0.4279454	4.0367943	5.42E-05
PLA2G2D	54.967326	1.7865725	0.4426121	4.0364295	5.43E-05
WNT10A	97.81642	1.8004751	0.446012	4.036831	5.42E-05
MME	1216.9384	-1.620184	0.4019032	-4.03128	5.55E-05
TMEM229A	10.935215	3.6904605	0.9162545	4.0277679	5.63E-05
NCCRP1	37.960441	2.2238022	0.55446	4.0107534	6.05E-05
MYBPH	27.885913	3.2566649	0.8123012	4.0091838	6.09E-05
IGSF9B	812.61005	3.1739056	0.7932077	4.0013548	6.30E-05
MMP12	11.709581	2.8844464	0.7208113	4.0016664	6.29E-05
C16orf59	33.970544	1.5566899	0.3893774	3.9978948	6.39E-05

KRT16P3	48.849514	3.361072	0.8406654	3.9981089	6.39E-05
PCSK1N	1341.6977	2.2018707	0.5507142	3.9982094	6.38E-05
LPO	7.9881076	2.851119	0.713619	3.995296	6.46E-05
SFN	1745.9864	2.25921	0.5660493	3.9911893	6.57E-05
ARHGEF4	337.70954	-1.561175	0.3917896	-3.984729	6.76E-05
CDH15	47.584119	2.1159168	0.5310404	3.9844739	6.76E-05
FOXJ1	1490.4459	1.8554889	0.4659687	3.9820036	6.83E-05
ACTBP7	4.3874965	4.7427003	1.193402	3.974101	7.06E-05
CD5L	27.174012	-1.956823	0.4937441	-3.963234	7.39E-05
LIPM	3.1254459	3.4929613	0.8814679	3.9626643	7.41E-05
MTND1P23	1579.8462	2.9530386	0.7460394	3.9582879	7.55E-05
NPY1R	74.687708	-1.541379	0.3893605	-3.958746	7.53E-05
ADRB3	15.314588	2.2751834	0.5751095	3.9560872	7.62E-05
SULT1C2	602.38456	1.7066699	0.4313941	3.9561738	7.62E-05
ALPK2	20.634228	3.0566534	0.7728823	3.9548756	7.66E-05
FAM150A	29.069119	2.2470214	0.5683422	3.9536414	7.70E-05
GPR110	446.42065	2.4192419	0.6124942	3.94982	7.82E-05
ELFN2	652.99789	2.0045508	0.5083891	3.942946	8.05E-05
EFNA2	5.4512177	5.003842	1.2705783	3.9382397	8.21E-05
GREB1	195.46372	2.3691609	0.6020438	3.9351969	8.31E-05
ACHE	284.20292	1.8122055	0.4610619	3.9305033	8.48E-05
INMT	7153.4516	-1.557896	0.3967515	-3.926629	8.61E-05
CAMK2B	50.924027	2.5395389	0.6478713	3.9198198	8.86E-05
PON3	597.72709	1.7906228	0.457739	3.9118858	9.16E-05
SLC30A2	5.9680111	2.0518563	0.5283277	3.8836811	0.0001029
TRIM51BP	3.757789	4.1643678	1.0729451	3.8812498	0.0001039
SLC22A31	3508.1406	1.8339363	0.474127	3.8680276	0.0001097
ADORA1	170.45632	1.5867619	0.4116023	3.8550852	0.0001157
BHLHA15	15.985164	1.9674026	0.5114222	3.8469248	0.0001196
CNGB1	16.170935	1.7607463	0.4581808	3.842907	0.0001216
GRIN1	38.894152	2.2201422	0.5776899	3.8431385	0.0001215
CA4	344.79608	-1.994234	0.5205573	-3.83096	0.0001276
ENTPD8	28.810993	1.8268434	0.4810606	3.7975328	0.0001461
LRRC2	59.299137	-1.595514	0.4201533	-3.797456	0.0001462
RBP2	32.146054	-2.075186	0.5462913	-3.79868	0.0001455
IGJ	4565.5481	2.0503655	0.5403833	3.7942797	0.0001481
POM121L10P	73.977793	1.6814666	0.4434241	3.7920054	0.0001494
ACTC1	10.871388	-2.555993	0.6764042	-3.778796	0.0001576
GALE	1120.3082	1.5005554	0.3973046	3.7768387	0.0001588
MAP7D2	36.846739	2.2605442	0.5984942	3.7770527	0.0001587
SLC35F3	37.185939	2.0549155	0.5439523	3.7777494	0.0001583
TESC	405.70875	1.9325905	0.5129412	3.7676648	0.0001648
GRM8	17.982481	-2.471972	0.6562426	-3.766856	0.0001653
CYP2J2	91.979477	1.5409864	0.4095795	3.7623618	0.0001683

KRT18P8	6.383718	3.4090295	0.9065309	3.7605223	0.0001696
SLC52A1	93.634783	1.5585793	0.4148014	3.7574111	0.0001717
SPINK5	494.97059	2.1133582	0.5636074	3.7496988	0.000177
PROC	28.791669	1.7517921	0.4676831	3.7456821	0.0001799
CCL15	12.080007	-2.611046	0.6971955	-3.74507	0.0001803
RET	64.12067	1.7285492	0.4619515	3.7418411	0.0001827
S100A3	83.949776	-1.774579	0.4772287	-3.718509	0.0002004
SAPCD2	74.329882	1.5043297	0.4049162	3.7151634	0.0002031
ZBED2	234.0991	-1.752443	0.4722183	-3.711087	0.0002064
CHRNA6	3.4980633	3.233177	0.8729277	3.7038315	0.0002124
UPK3B	3183.5125	-2.012641	0.5434276	-3.703604	0.0002126
C1orf65	3.3528521	3.3665123	0.9094882	3.7015458	0.0002143
RGS9BP	85.317029	-1.61961	0.4378741	-3.698804	0.0002166
SOX11	16.122196	2.8070371	0.7597233	3.6948152	0.00022
PAH	2.9656527	3.2766666	0.8896108	3.6832584	0.0002303
C6orf222	7.1812835	4.0811505	1.1089519	3.6801871	0.0002331
PNMT	49.853179	-1.923385	0.522613	-3.680324	0.0002329
UPK1B	5.2407731	-2.341312	0.6365848	-3.677927	0.0002351
CLDN5	3483.3641	-1.695926	0.4612416	-3.676872	0.0002361
ESPNP	12.203259	2.5554973	0.6950071	3.6769368	0.0002361
PPAP2C	556.28333	1.6914906	0.4611911	3.667657	0.0002448
EGF	68.731675	1.7320968	0.4732538	3.6599747	0.0002522
MB	26.963073	3.1599294	0.8645757	3.6548905	0.0002573
IVL	10.248385	3.1039992	0.8496505	3.6532659	0.0002589
TBC1D27	13.843639	2.5724739	0.7059114	3.644188	0.0002682
DPEP1	25.803488	2.1595515	0.5928451	3.6426909	0.0002698
PSAT1	50.82931	1.6715492	0.4589003	3.6425104	0.00027
NPSR1-AS1	7.8675333	5.5917425	1.5395785	3.6319958	0.0002812
PCSK2	97.187225	2.2956069	0.6322221	3.6310134	0.0002823
LINC00896	30.450022	1.7634166	0.4857268	3.6304702	0.0002829
KRT14	4.8413065	3.5528412	0.9805098	3.6234631	0.0002907
COL1A1	21010.997	2.2687693	0.6268904	3.6190843	0.0002956
COL22A1	34.42162	2.3027795	0.6365626	3.6175228	0.0002974
PITX1	37.46803	2.9525979	0.8161686	3.6176323	0.0002973
SPOCK3	8.6582874	2.6537849	0.7340341	3.615343	0.0003
SYN2	55.642661	-1.66403	0.4606878	-3.612056	0.0003038
PMCH	3.1593154	3.0565245	0.8472441	3.6076078	0.000309
WNT3A	302.73436	-1.557723	0.4323882	-3.602602	0.000315
LCN12	47.844816	1.7431262	0.4859539	3.5870197	0.0003345
IYD	298.66184	2.1043092	0.5875971	3.581211	0.000342
KRT16	3.2407815	4.6225217	1.2910369	3.5804721	0.000343
MZB1	336.03793	1.6891218	0.4725054	3.5748199	0.0003505
RNASE2	29.34503	2.4165834	0.6762954	3.5732661	0.0003526
IGLL5	3758.0375	1.8450191	0.5168153	3.5699776	0.000357

TRIM54	14.442269	2.4333063	0.6821331	3.5672017	0.0003608
CDO1	227.30641	-1.517195	0.4255355	-3.565377	0.0003633
HTR3C	26.832704	-1.999829	0.5611335	-3.563909	0.0003654
SSTR3	4.9957889	2.2193703	0.6237101	3.5583365	0.0003732
CLEC3B	4217.8254	-1.738434	0.4893601	-3.552465	0.0003816
NETO1	5.5886948	4.3883901	1.2359329	3.5506702	0.0003843
ISLR2	52.22928	1.701933	0.4824156	3.5279393	0.0004188
PNOC	17.375417	1.5210507	0.4331035	3.5119802	0.0004448
MYO16-AS1	8.5824022	-2.000577	0.5706811	-3.505595	0.0004556
DGCR5	60.836331	1.6687747	0.476347	3.5032754	0.0004596
SORCS2	852.41432	1.5093415	0.4310252	3.5017476	0.0004622
RBMXL2	5.7765741	-1.635563	0.4673605	-3.499574	0.000466
GDNF	6.7861515	1.7445365	0.4987284	3.4979691	0.0004688
TNFRSF13C	25.659347	2.6510445	0.7579345	3.4977223	0.0004692
FOXA3	40.526777	1.7116796	0.4902115	3.4917166	0.0004799
PHLDA2	510.76831	1.9161889	0.5502062	3.4826741	0.0004964
GPR84	23.743594	1.5200882	0.4366201	3.4814891	0.0004986
MMP9	181.98955	1.9186274	0.5535624	3.4659639	0.0005283
CEACAM8	143.59722	2.5514766	0.7364124	3.4647388	0.0005307
KCNQ2	5.2668337	3.1610662	0.9146863	3.4559022	0.0005485
KCNJ12	68.623619	-1.527591	0.4424058	-3.452919	0.0005546
KCNQ3	578.82059	1.6335129	0.4730121	3.4534273	0.0005535
DNAJB13	92.079389	2.0807846	0.6030557	3.4504021	0.0005598
CCNO	58.076254	2.039498	0.5911793	3.4498808	0.0005608
RHCE	8.7849415	2.529626	0.733472	3.4488376	0.000563
PITX2	5.2040219	4.9856038	1.4490348	3.4406377	0.0005803
MAGED4B	160.17483	1.580053	0.4599899	3.434973	0.0005926
MYOC	77.435532	-2.146002	0.625735	-3.42957	0.0006045
LTB	602.16006	1.5563671	0.4539493	3.428504	0.0006069
SGCG	76.335205	-1.736112	0.5063398	-3.428749	0.0006064
KRT16P2	392.84113	2.5813019	0.7531208	3.4274739	0.0006092
GPIHBP1	689.62058	-1.548188	0.4537731	-3.411811	0.0006453
CD177	26.807052	1.5920414	0.4669797	3.4092302	0.0006515
CGB7	40.098675	1.5058557	0.4425734	3.4024994	0.0006677
BARX1	6.503833	3.4376866	1.0121085	3.3965595	0.0006824
PTBP1P	19.586691	2.9715396	0.8745751	3.3976951	0.0006796
LRRC31	98.387344	1.8708329	0.5512514	3.3937925	0.0006893
STK32B	125.35162	1.6737341	0.4931664	3.3938528	0.0006892
IL12B	10.20025	1.819526	0.5365404	3.3912188	0.0006958
LYZ	14526.064	1.5509949	0.4573588	3.3911994	0.0006959
CXCL14	691.05633	1.9792144	0.584565	3.38579	0.0007097
LINC01082	18.835507	-2.159498	0.638431	-3.382508	0.0007183
MTRNR2L9	2.1631445	-2.574415	0.7614915	-3.380754	0.0007229
MMP1	126.67042	3.2338011	0.9600081	3.3685144	0.0007557

TLR10	44.161496	1.597393	0.474486	3.3665753	0.0007611
AQP2	13.915503	2.4365475	0.7241958	3.3644871	0.0007669
SPINK2	9.4682504	2.6824348	0.7980445	3.3612597	0.0007759
C20orf197	17.807491	1.7101598	0.5093297	3.3576675	0.000786
OLIG1	41.824487	1.8553855	0.5537008	3.3508812	0.0008055
PLAC9	822.09388	-1.534498	0.4583144	-3.348134	0.0008136
GAPDHP55	3.4071967	4.6743431	1.3988019	3.3416763	0.0008327
HRH4	10.071671	2.1298525	0.6375036	3.3409263	0.000835
APOA1	27.935394	-1.6311	0.4890001	-3.335581	0.0008512
CPNE4	18.238694	1.9538325	0.5858162	3.335231	0.0008523
CNFN	35.335011	1.6940263	0.5087899	3.3295205	0.00087
PLD5	65.838665	1.7204625	0.5165567	3.3306362	0.0008665
TM4SF4	15.983912	2.0592474	0.6184366	3.3297629	0.0008692
FCN3	1719.5987	-1.871914	0.5634992	-3.321947	0.0008939
BARX2	26.13738	2.1669171	0.6536308	3.3152003	0.0009158
DLX3	78.057773	1.5826002	0.477508	3.3142906	0.0009188
ABCA12	20.936834	2.7823857	0.8397082	3.3135149	0.0009213
DUX4L4	39.13809	4.6832104	1.4139725	3.3120943	0.000926
ZSCAN4	17.329707	2.4348728	0.7352098	3.3118068	0.000927
FAM135B	10.483395	2.5970997	0.7856397	3.3057136	0.0009473
NPW	33.243595	2.0255757	0.6127213	3.3058678	0.0009468
RAMP3	2210.0445	-1.504867	0.4553395	-3.304934	0.00095
HS6ST3	16.955305	4.0591247	1.2284555	3.3042505	0.0009523
TMEM213	160.89981	2.0279298	0.6138063	3.3038597	0.0009536
LRRC52	13.575431	1.9446207	0.5895012	3.2987559	0.0009711
SLC1A7	232.45143	1.7236507	0.5229282	3.2961515	0.0009802
COL11A1	37.507805	2.6374724	0.8003931	3.2952212	0.0009834
LCT	13.736419	2.8319868	0.8626729	3.282805	0.0010278
BCRP3	98.348929	1.5392757	0.4703702	3.2724768	0.0010661
C12orf42	4.5644562	2.3649951	0.7236567	3.2681176	0.0010827
LINC00162	11.221298	-1.982051	0.6068298	-3.266238	0.0010899
AREG	496.51093	1.5575237	0.4774232	3.2623547	0.0011049
TFAP2A	76.45314	1.7396885	0.534455	3.2550703	0.0011336
DPYS	20.078995	1.5980041	0.4911337	3.2537049	0.0011391
CMA1	24.1453	1.8031133	0.5544037	3.2523469	0.0011446
CLDN3	1359.0119	1.5933585	0.4899838	3.2518592	0.0011465
PTCHD1	180.96174	2.1611136	0.6648944	3.2503111	0.0011528
AMPD1	21.394593	1.622222	0.5023134	3.2295021	0.0012401
CYP19A1	13.074725	2.1029942	0.6525888	3.2225409	0.0012706
ANTXRL	2.0126621	3.0126487	0.9372899	3.2142124	0.001308
GPR112	17.653511	-2.075146	0.6455827	-3.214377	0.0013073
AMY1C	386.09353	2.6307946	0.819239	3.2112662	0.0013215
HOXD4	17.363528	2.2559477	0.7024717	3.211443	0.0013207
TERT	3.1289454	3.883603	1.210168	3.2091437	0.0013313

HBZ	5.3538677	-2.05557	0.6412127	-3.205754	0.0013471
ONECUT2	12.880039	1.7675301	0.5521691	3.2010665	0.0013692
CKMT1B	54.145618	1.7894807	0.5601626	3.1945735	0.0014004
ONECUT1	3.0204253	3.8492019	1.2052092	3.1938038	0.0014041
CACNA1I	28.699258	1.6514566	0.5173792	3.1919657	0.0014131
SLC25A48	2.985677	3.6337011	1.139842	3.187899	0.0014331
FCAMR	7.70637	2.360294	0.7418417	3.1816681	0.0014643
HTR3A	2.6471178	2.7400211	0.8611717	3.1817362	0.001464
APOA5	2.3192313	-1.880996	0.593318	-3.170301	0.0015228
TMEM155	13.023073	1.6861134	0.5318399	3.1703401	0.0015226
PLA2G10	146.17467	1.5297444	0.4830547	3.1668138	0.0015412
CES3	134.05643	1.5221424	0.4815896	3.1606627	0.0015741
DHDH	37.798741	1.595771	0.5048748	3.160726	0.0015738
ADAMDEC1	17.245021	2.0332455	0.6435911	3.1592193	0.0015819
AIM2	23.539715	1.5661281	0.4961917	3.1562965	0.0015979
FAM3B	157.65487	1.5562878	0.4936725	3.1524702	0.001619
SRMS	8.4052057	2.4128837	0.7656815	3.1512891	0.0016255
HECW1	73.479139	2.0339256	0.645762	3.1496523	0.0016346
TNFRSF9	26.518573	2.0265941	0.6449105	3.1424425	0.0016754
HOXD-AS2	9.1076925	2.6844966	0.8579301	3.1290388	0.0017538
CCK	7.5967218	-2.199157	0.7031643	-3.127514	0.0017629
SLC30A3	19.054546	1.5797935	0.5055684	3.1247868	0.0017793
SEPT10P1	3.746247	-1.582521	0.5074466	-3.118596	0.0018172
SYNPO2L	64.126884	-1.763167	0.5656757	-3.116922	0.0018275
OPRK1	15.254362	2.0966613	0.6728215	3.1162224	0.0018318
UGT2B15	3.4927459	3.5977345	1.1553057	3.1140974	0.0018451
SLCO1B3	20.111753	4.9928934	1.6044573	3.1118892	0.0018589
BEX1	21.394149	-1.642244	0.528125	-3.109575	0.0018736
DLK1	5.1108882	4.0727882	1.3112411	3.1060559	0.001896
C16orf89	6125.6781	1.621834	0.5222685	3.1053643	0.0019004
WASIR2	7.3208967	1.7361356	0.56054	3.0972553	0.0019532
C1orf53	39.502099	1.6294719	0.5264044	3.0954756	0.001965
DNAH11	953.43081	2.0841828	0.6732595	3.0956605	0.0019638
LINC00958	5.9907599	1.8489495	0.5974455	3.0947582	0.0019697
LYPD2	15.805591	-1.95886	0.6341758	-3.088827	0.0020095
MIOX	2.0661874	2.9855648	0.968229	3.0835317	0.0020456
MYADML2	2.6518468	2.7179085	0.8827386	3.0789506	0.0020773
ITLN2	494.93011	-1.83246	0.5953584	-3.07791	0.0020846
C10orf55	60.105474	1.7489652	0.5683997	3.0769991	0.002091
CD164L2	104.47237	1.8307667	0.5952041	3.0758636	0.0020989
PTPRH	7.805388	2.2098876	0.7183935	3.076152	0.0020969
CTSG	114.33855	1.5280258	0.4972973	3.0726604	0.0021216
IDO1	158.60747	-1.503129	0.4894452	-3.071087	0.0021328
CTNND2	118.98313	-1.506472	0.4913482	-3.065997	0.0021695

COLEC10	28.525163	-1.637916	0.5360956	-3.055269	0.0022486
CASR	26.639209	1.9197435	0.6286977	3.0535242	0.0022617
GOLT1A	209.86897	1.5398973	0.5044393	3.052691	0.002268
COL10A1	425.38929	1.5981726	0.5239792	3.0500689	0.0022879
RS1	69.058014	-1.522473	0.4998684	-3.045747	0.002321
TRIM46	272.64034	1.8401306	0.6054626	3.0392145	0.002372
HS3ST6	55.506749	2.1433954	0.7057034	3.037247	0.0023875
ALDH3A1	452.72223	1.7966128	0.591929	3.0351828	0.0024039
UBE2Q2P2	24.549782	2.1449581	0.7067942	3.0347706	0.0024072
CCL17	75.56194	2.0005346	0.6600087	3.0310729	0.0024369
TM4SF1-AS1	5.2362003	1.8741581	0.6181705	3.031782	0.0024311
TUBB8	5.3666601	2.3889324	0.7902203	3.023122	0.0025018
ANKRD18B	4.8229519	2.6749312	0.8857466	3.0199733	0.002528
LINC00670	7.0983666	-1.622117	0.5395176	-3.006607	0.0026418
ITGA11	286.60402	1.5625514	0.519887	3.0055597	0.0026509
B3GAT1	212.62384	1.9358937	0.6445784	3.0033485	0.0026703
NAPSA	45079.448	1.6381475	0.5452722	3.0042745	0.0026622
POM121L7	3.4788399	3.0282251	1.0082527	3.0034387	0.0026695
ADAMTS18	15.592107	2.5784734	0.8591444	3.0012108	0.0026891
SIX1	166.80607	1.811505	0.6040932	2.9987178	0.0027112
SLC6A18	4.2566805	3.7957188	1.2659272	2.9983705	0.0027143
PROM1	111.48666	2.043766	0.6827043	2.9936328	0.0027568
WFDC2	4385.0475	1.6785067	0.5616944	2.9882915	0.0028054
IGFBPL1	34.319252	2.0821421	0.6975356	2.9849977	0.0028358
PI15	24.941425	1.5624431	0.5240401	2.9815336	0.0028681
FBXO2	515.20487	1.5120069	0.5074528	2.9796009	0.0028862
IGKV1D-27	2.7031997	2.223063	0.746254	2.9789629	0.0028923
GABBR2	50.515079	1.6268282	0.5465437	2.9765746	0.0029149
ABCG4	6.0924321	1.9517181	0.6562807	2.9739077	0.0029403
PTCSC3	10.941779	1.896709	0.6387878	2.9692319	0.0029855
SLC5A1	113.2852	-1.605107	0.5421222	-2.960784	0.0030686
HOXC10	15.775309	4.9367205	1.6736269	2.9497139	0.0031807
CREG2	3.4915724	1.7803859	0.6052786	2.9414322	0.003267
NPC2	15047.139	1.6642139	0.5662795	2.938856	0.0032943
SLCO4A1	127.1481	-1.599031	0.5444668	-2.936874	0.0033154
ABCC11	11.621609	1.7765295	0.6064201	2.929536	0.0033947
NT5C1A	13.056003	2.7709922	0.9488237	2.92045	0.0034953
TTR	6.8204504	3.1197128	1.0730413	2.9073557	0.003645
FBP2	7.1783245	-1.903116	0.6548886	-2.906015	0.0036606
APOH	58.966113	2.0226199	0.6961386	2.9054844	0.0036669
HCAR1	20.777269	1.7759882	0.6119863	2.9020062	0.0037078
C1QL2	24.330507	2.7074047	0.9345877	2.8968973	0.0037687
MOGAT2	2.0189168	3.2745539	1.1304101	2.8967839	0.0037701
HOXC13	8.855844	6.0597343	2.0921031	2.8964797	0.0037738

IGLVI-70	2.0843504	3.6588774	1.2675752	2.8865169	0.0038953
PLA2G2F	3.545637	3.5198084	1.2211599	2.8823484	0.0039472
PI16	179.87836	-1.757302	0.6099553	-2.881033	0.0039637
GAD1	20.772078	2.0392573	0.7079611	2.880465	0.0039709
DUX4L5	8.607588	5.7205502	1.9904257	2.8740336	0.0040527
LINC00460	5.942427	2.2762898	0.7920658	2.8738645	0.0040548
TRPM5	9.7837312	1.9352358	0.6756403	2.864299	0.0041793
RPRML	24.395489	1.7976332	0.6279674	2.8626217	0.0042015
CHRM1	90.860244	-1.506852	0.527337	-2.857475	0.0042703
KCNJ3	4.0006583	-1.819505	0.6379519	-2.852104	0.0043431
PGLYRP4	8.2886183	2.5680072	0.9003848	2.8521217	0.0043428
KIAA0125	9.0617429	1.8239686	0.6406521	2.8470503	0.0044126
TP63	202.92992	1.6244503	0.5709312	2.8452644	0.0044375
AREGB	30.806827	2.3921866	0.8425157	2.8393377	0.0045207
ECEL1	12.435638	2.3316927	0.8249005	2.8266352	0.004704
LINC00494	5.893	1.9756351	0.6991078	2.8259377	0.0047142
DLEU7-AS1	2.2056001	2.9449824	1.0424827	2.82497	0.0047285
KRT5	63.381407	2.1014247	0.7462902	2.815828	0.0048652
SOSTDC1	267.43816	-1.61868	0.575679	-2.811775	0.0049269
IGFL2	3.3382275	2.1395143	0.7616906	2.8089021	0.0049711
TFAP2B	9.5669834	2.9647796	1.055384	2.8091951	0.0049666
FGL1	7.7317831	1.7213259	0.6142219	2.8024496	0.0050716
SLC24A2	5.9982409	2.1731424	0.7754608	2.8023885	0.0050726
SLC17A8	3.1875489	2.1971239	0.7847304	2.7998456	0.0051127
NTS	15.944369	-2.068007	0.7390625	-2.798149	0.0051396
C12orf74	21.913276	1.8323533	0.6550777	2.7971541	0.0051555
LINC00593	3.2994531	3.1240905	1.1169079	2.7970887	0.0051565
FAM83A-AS1	2.3572989	2.673062	0.9562483	2.795364	0.0051841
CNR2	24.468106	1.6045916	0.57489	2.7911282	0.0052525
ANKS4B	4.9059683	2.81259	1.0089928	2.7875222	0.0053113
FAM129C	84.247106	1.5956168	0.5740285	2.7796823	0.0054412
DUX4L6	13.107351	5.5240012	1.9892316	2.7769523	0.0054871
ANKRD20A8P	6.9028962	2.8259573	1.0205498	2.7690539	0.0056219
GOLGA6L10	3.4626568	2.9280003	1.0574167	2.7690127	0.0056226
KRTAP5-1	10.469779	1.562337	0.5649052	2.7656626	0.0056807
RHOV	63.311885	1.5810098	0.5728976	2.7596726	0.0057859
CLEC4M	44.591497	-1.582044	0.57393	-2.75651	0.0058422
LINC00524	1.7009392	3.3964808	1.2342714	2.7518103	0.0059267
FGFBP2	79.914314	-1.612383	0.5870343	-2.746659	0.0060206
METTL21C	4.5182107	-1.547933	0.563525	-2.746875	0.0060166
HOXC-AS2	3.6240806	3.1026616	1.1300437	2.7456119	0.0060398
SERPIND1	130.94122	1.6166442	0.5892066	2.7437647	0.0060739
BTNL3	5.1136018	-2.722497	0.9938495	-2.739346	0.0061562
OXGR1	4.1316301	-1.520102	0.5549055	-2.73939	0.0061553

MYBPHL	71.823192	1.5271979	0.5589065	2.7324748	0.006286
HNF1A-AS1	9.3162097	2.1501863	0.7870574	2.7319309	0.0062964
UNC13C	38.306222	2.4597544	0.9020038	2.726989	0.0063915
SORCS1	9.6100431	1.639548	0.602103	2.7230359	0.0064685
FOXH1	4.0540719	1.7678485	0.6506203	2.7171741	0.0065842
LMX1B	15.938753	1.8579076	0.6841785	2.7155305	0.006617
B4GALNT4	44.0501	1.8056855	0.6662705	2.7101387	0.0067255
HIST2H2BC	14.742718	1.5864531	0.5859696	2.7073984	0.0067813
TFAP2A-AS1	11.355659	1.6236092	0.6028246	2.6933359	0.0070741
RBFOX1	6.6122495	3.0097392	1.117977	2.6921299	0.0070997
DUSP9	4.7355444	2.3419387	0.8720826	2.6854553	0.0072431
COL2A1	9.18771	2.3889927	0.8900732	2.6840407	0.0072738
GCOM1	563.79558	-1.56903	0.5862114	-2.67656	0.0074382
HS3ST4	8.2647092	2.5262381	0.9439206	2.6763249	0.0074434
TLX2	8.9317969	1.7918094	0.6697938	2.6751658	0.0074692
SLN	6.8881403	-1.997567	0.74704	-2.673976	0.0074958
OLIG2	8.3553728	2.5698729	0.9613417	2.6732149	0.0075128
CCDC54	2.2152303	-1.816854	0.6807823	-2.668774	0.0076129
TMEM132D	181.83532	1.8812046	0.7053844	2.6669211	0.007655
HRASLS	6.0180511	1.6644397	0.6248806	2.6636122	0.0077307
CCDC155	14.794323	1.8316412	0.68801	2.6622304	0.0077625
TMED10P2	5.1305025	-1.589078	0.5974599	-2.659724	0.0078205
SLC6A7	8.6170653	2.0028415	0.7534605	2.6581905	0.0078561
SLCO1A2	101.55329	-1.779895	0.6704638	-2.654722	0.0079374
MYEOV	13.460925	1.5344208	0.5786833	2.6515726	0.0080118
LINC00336	4.1737293	1.8826444	0.7107701	2.6487387	0.0080793
BMP8A	54.954897	2.2139236	0.8363528	2.6471169	0.0081181
CLEC17A	9.622293	2.0503	0.778188	2.6347103	0.0084209
GUCY1B2	57.467768	2.2001423	0.8362513	2.6309584	0.0085144
GPR15	5.3618129	2.9012797	1.1037517	2.6285621	0.0085747
CELA2B	9.5704748	-1.565325	0.5956369	-2.627986	0.0085892
INSM1	8.7870205	1.7783182	0.6802745	2.6141185	0.0089458
SH3GL3	74.977006	-1.544	0.5908972	-2.612975	0.0089758
ABCC12	3.5446871	1.5665072	0.5996582	2.6123335	0.0089926
PGC	11568.256	1.6963836	0.6496572	2.611198	0.0090226
HCN1	2.7462877	2.4821639	0.9533333	2.6036685	0.0092232
GSTA1	230.32628	1.5165158	0.5851302	2.591758	0.0095487
LINC01013	3.8698019	1.6273665	0.6285399	2.5891222	0.0096221
DRGX	10.251526	2.4621229	0.9522814	2.5854992	0.0097238
CRHR2	8.7869542	1.9098914	0.7402183	2.5801731	0.0098751
HBA2	5675.9606	-1.533984	0.5950624	-2.577854	0.0099416
C16orf96	20.79556	1.5033898	0.5838763	2.5748427	0.0100286
PRSS3	1.7156261	2.1824906	0.8489852	2.570705	0.0101492
GUCA2B	3.6359596	-1.997418	0.7775578	-2.568836	0.0102041

SPHKAP	8.5383495	-1.842724	0.718328	-2.565296	0.0103088
MUC22	6.347458	1.666461	0.652575	2.5536699	0.0106594
PLA2G4D	8.1779516	1.5454282	0.6054179	2.5526636	0.0106903
OGDHL	4.9502575	2.2471488	0.8804146	2.5523757	0.0106991
SPATA32	11.955651	1.5814482	0.6239966	2.534386	0.0112645
CHIA	397.67886	1.9174801	0.7568403	2.5335332	0.0112919
SZT2-AS1	13.294272	1.5367972	0.6082313	2.5266657	0.0115151
DNAJB3	2.1118746	3.984245	1.5782219	2.5245151	0.0115858
CACNA1B	8.9212809	2.1181609	0.8398516	2.5220656	0.0116668
TRIM51EP	2.5241719	3.5524445	1.4084458	2.5222444	0.0116609
IL11	10.534611	1.6970225	0.6751948	2.5133821	0.011958
CRYM-AS1	19.312557	1.6341293	0.6503913	2.5125327	0.0119868
C15orf48	132.26845	1.5666765	0.6240837	2.5103629	0.0120607
TIMM8AP1	4.1677904	1.6999329	0.6780004	2.5072741	0.0121666
RARRES2P4	10.554361	2.1025219	0.8387302	2.5067916	0.0121833
EEF1A1P7	1.940755	-1.87539	0.7510862	-2.496903	0.0125283
SALL1	6.8202741	3.4159712	1.3708847	2.4918006	0.0127097
DMRT3	5.4688451	3.0931658	1.2417156	2.491042	0.0127369
IL1RAPL2	1.9553257	3.3244004	1.3345539	2.49102	0.0127377
GUCA2A	7.5935556	-1.554577	0.6241323	-2.49078	0.0127463
ADAMTS16	45.998135	1.6962847	0.6818321	2.4878335	0.0128524
OR6K3	2.9634186	-1.577976	0.6348873	-2.485443	0.012939
FAT3	1113.4394	-1.797989	0.7242837	-2.482438	0.0130487
ANKRD1	694.6023	-1.753049	0.7066946	-2.480632	0.013115
NMU	3.4465243	2.0388672	0.8233153	2.4764113	0.0132711
CCR8	8.3604636	1.6471955	0.6655351	2.4749941	0.0133238
DGKK	6.2824552	-1.581493	0.6402465	-2.470131	0.0135063
DCC	85.46914	-1.800145	0.7297848	-2.466679	0.0136372
SOST	7.0975728	-2.050973	0.8326211	-2.463273	0.0137675
SNORA11D	2.3663941	3.3165686	1.3518275	2.4533964	0.0141514
SNORA11E	2.3663941	3.3165686	1.3518275	2.4533964	0.0141514
FAM25C	3.0779609	-1.896313	0.7757066	-2.444627	0.0145002
WNT6	12.55352	1.5465001	0.6334294	2.441472	0.0146275
PCP4	11.995619	1.9467127	0.7978705	2.4398855	0.0146919
HOXC11	8.8538633	6.0539983	2.4854277	2.4357974	0.014859
CKMT1A	40.133427	1.6755216	0.6891218	2.4313867	0.0150412
LEMD1-AS1	7.040087	2.000918	0.8228762	2.4316148	0.0150317
UNGP3	2.8031664	-1.587449	0.6529698	-2.431122	0.0150521
LHX9	31.013402	2.1157367	0.8725931	2.4246544	0.015323
KIRREL2	2.0306767	2.3325125	0.9642156	2.4190776	0.0155599
SCG5	10.13459	1.5335083	0.6339091	2.4191296	0.0155577
DCST1	3.5992274	1.7892814	0.7411503	2.414195	0.01577
HSD3BP5	21.491845	-1.560383	0.6494816	-2.402505	0.0162832
HHLA2	286.06111	1.980991	0.8251231	2.400843	0.0163574

SMIM17	2.3245646	1.6359497	0.682092	2.3984296	0.0164655
ZAR1	26.493536	1.8508991	0.7715629	2.3988959	0.0164446
XAGE3	2.5120597	2.2969381	0.9579843	2.3976783	0.0164994
TNFSF11	2.7535576	1.7881146	0.7464977	2.3953384	0.016605
ARX	66.648673	2.0915059	0.8740071	2.3930078	0.0167109
HOXC-AS5	2.1543867	4.0339139	1.6858476	2.3928105	0.0167199
XAGE1B	30.276137	2.357131	0.9857419	2.3912252	0.0167922
DKFZP434E1119	4.5109654	1.7032368	0.7149554	2.382298	0.017205
VGF	7.2128393	1.5252801	0.6402287	2.3823989	0.0172003
TMEM75	30.631847	2.9705702	1.253059	2.3706547	0.0177566
BRDT	7.6155017	3.6364287	1.5348197	2.3692871	0.0178224
KCNA7	7.5688726	2.2222252	0.9423531	2.3581662	0.0183655
POTEG	2.7777961	3.7794194	1.6032499	2.3573489	0.0184059
NKAIN4	3.566385	1.7602182	0.7475322	2.3547054	0.0185374
SCGB3A2	13808.383	1.6228696	0.6915198	2.3468157	0.0189346
STEAP3-AS1	29.218619	1.7399033	0.7421028	2.3445581	0.0190496
MTND5P31	1.8240431	3.4481536	1.4731297	2.3406992	0.0192477
UBE2Q2P3	27.486324	1.5162418	0.6479235	2.3401556	0.0192757
PLAC1	1.7720885	2.7406784	1.1717219	2.3390178	0.0193345
C5orf46	7.5905427	2.3955547	1.0251204	2.3368519	0.0194469
RANBP20P	6.4200047	2.2207572	0.952063	2.3325738	0.0196705
HMGN2P15	2.9818447	-1.589726	0.6835	-2.325861	0.020026
WISP1-UT1	1.8046783	-2.288213	0.9862064	-2.320217	0.0203291
MOGAT1	2.5177505	-1.53093	0.6607153	-2.31708	0.0204994
HNF4G	16.41459	1.6122671	0.6971626	2.3126128	0.0207439
NPHS1	2.4619609	2.5470244	1.1030292	2.3091179	0.020937
PRAMENP	1.7319635	2.7914398	1.2117073	2.3037245	0.0212381
XAGE1D	5.5789204	1.857849	0.8068429	2.3026155	0.0213005
OXT	9.1826346	1.8411469	0.8015398	2.2970126	0.021618
BFSP2	2.5788831	1.5665579	0.6833206	2.2925666	0.021873
HECW1-IT1	1.7369229	2.9891387	1.3055798	2.2895106	0.0220497
LHX2	1.9999448	1.9801098	0.8648914	2.2894315	0.0220543
RPS6P12	2.5900175	2.9229495	1.2765265	2.289768	0.0220348
RN7SL506P	3.5497218	3.0633202	1.3451174	2.2773627	0.0227646
RGR	1.8057936	-1.832332	0.8052555	-2.275467	0.0228779
POM121L3P	1.7047785	2.5065102	1.1028499	2.2727574	0.0230408
CDC42P4	1.7282349	-1.587042	0.7005971	-2.26527	0.0234961
MFAP5	107.62016	-1.563693	0.6914395	-2.261503	0.0237281
C17orf99	2.1387823	1.5441641	0.6835993	2.2588733	0.0238913
SNORD105B	2.0620039	3.5912606	1.5899359	2.2587455	0.0238992
PTMAP1	3.2780046	2.3992974	1.0655527	2.2516928	0.0243417
KRT8P41	3.2496271	2.237341	0.9950583	2.2484523	0.0245474
BMS1P8	1.967324	2.0763735	0.9241225	2.2468597	0.024649

LINC00520	2.1123498	-1.922208	0.8583195	-2.239502	0.0251233
SLC10A2	12.70182	2.3503622	1.0496997	2.2390807	0.0251507
FKSG48	2.2335426	1.6707584	0.7481352	2.2332307	0.0255337
CICP13	1.7499244	2.6141542	1.1744943	2.22577	0.0260296
PGAM1P2	2.6054146	-2.212124	0.9947546	-2.223788	0.0261627
EMX1	5.3435088	1.9072288	0.8612229	2.2145588	0.0267904
USH2A	19.579194	-1.53889	0.6952499	-2.213434	0.0268677
CNOT6LP1	7.3493259	-1.501729	0.6815673	-2.203346	0.0275704
OMP	2.3508117	2.282486	1.0360702	2.2030225	0.0275932
POU4F1	2.4613338	2.5915521	1.1754033	2.2048194	0.0274668
TMSB15A	34.962865	-1.639776	0.7441084	-2.203679	0.027547
KCNJ6	31.497915	1.5284681	0.6976588	2.1908533	0.0284624
MIR4500HG	1.8702783	-1.728817	0.7927044	-2.18091	0.0291901
DDX50P2	1.912718	-1.935466	0.8903575	-2.173808	0.0297196
BPIFA2	2.1662444	2.248927	1.0351284	2.1726067	0.0298099
GPAA1P1	15.232945	1.8985513	0.8737909	2.1727753	0.0297972
MMP3	3.6145345	1.6688452	0.7776096	2.1461221	0.0318632
FGB	2.1429308	2.9977471	1.4004341	2.1405841	0.0323076
OPRD1	3.5030647	3.2912081	1.5378379	2.1401528	0.0323424
GALR3	2.0964092	1.7322654	0.8101183	2.1382871	0.0324935
NHLH2	28.855939	1.5415478	0.724003	2.129201	0.0332376
LINC00443	3.7142042	-1.542758	0.725847	-2.125459	0.0335484
SYCE3	4.1687663	1.5160321	0.7137479	2.1240444	0.0336664
AMER2	32.828334	-1.684869	0.798277	-2.110632	0.0348039
PRTN3	15.751458	1.5156359	0.7192247	2.107319	0.0350899
BTBD17	1.8061502	2.1168394	1.0086625	2.0986597	0.0358469
PHEX-AS1	2.2611954	2.1538093	1.0270559	2.0970711	0.0359873
PAX1	1.8318501	2.8213932	1.3461982	2.0958231	0.0360979
FAM83B	7.0056467	1.8414516	0.8818157	2.08825	0.0367753
HNRNPDP1	1.974244	2.4787541	1.1893904	2.0840543	0.0371552
SPRR2D	2.5917409	2.5154321	1.2089281	2.0807127	0.0374602
SNX18P13	19.655988	1.704121	0.8206621	2.0765196	0.0378459
ZNF888	3.4069274	1.7108094	0.8242051	2.0757083	0.0379209
DSC3	5.6808237	1.5050682	0.725509	2.0744997	0.0380329
ENO1-IT1	5.7434083	2.5641681	1.2420236	2.0645084	0.0389695
LINC00880	2.7116677	1.9601603	0.9526939	2.0574923	0.0396389
PNMA6B	5.6995634	5.3977687	2.6235492	2.05743	0.0396449
ENO1-AS1	2.0748221	1.5778404	0.7682305	2.0538632	0.0399889
EGR4	6.3844284	1.9033593	0.9315748	2.0431632	0.0410363
XAGE1A	3.4969896	1.679241	0.8252985	2.0347074	0.0418803
LY6H	29.041546	1.5437514	0.759737	2.031955	0.0421582
XAGE1E	3.4457086	1.7203773	0.8494762	2.0252212	0.0428447
ERN2	182.13509	1.6393819	0.8155187	2.0102321	0.0444066
RNU4-25P	4.8680542	2.3381604	1.1686439	2.0007467	0.0454197

ATP13A4-AS1	93.530782	1.5084085	0.7570537	1.9924723	0.0463193
SLC6A5	1.7847557	3.031454	1.5244406	1.9885681	0.0467489
ACTBL2	2.5606796	1.5638651	0.7868	1.9876272	0.0468529
HNRNPA1P29	1.9009964	-2.286514	1.1552545	-1.97923	0.0477902
BMS1P18	6.0781782	2.0223622	1.0258272	1.9714452	0.048673
IL17REL	9.1699181	1.7463285	0.8904831	1.9611024	0.0498671
ACA64	1.1706874	2.1715741	1.0540314	2.0602556	0.0393741
AGXT	1.5257391	-2.587853	0.9257701	-2.795352	0.0051843
ALOXE3	1.511415	1.8820667	0.8369507	2.2487186	0.0245304
ANKRD30BL	1.6232625	3.29677	1.4850976	2.2199012	0.0264255
AVPR1B	1.559164	2.4038132	1.1349245	2.1180379	0.0341719
BSN-AS2	1.277294	-1.882455	0.6206908	-3.032838	0.0024227
C11orf86	1.1559563	3.1109756	1.3628141	2.2827586	0.0224446
C4BPAP1	1.5668796	2.2837963	0.9166452	2.4914726	0.0127215
C5orf60	1.6788946	1.7564508	0.7883882	2.2279008	0.0258871
C6orf100	1.3543503	2.3375173	0.9838769	2.375823	0.0175099
CCKBR	1.3964686	-2.708482	0.7872246	-3.440545	0.0005805
CHRNB3	1.5898443	2.9027652	1.159627	2.5031888	0.012308
CLEC2L	1.2642132	-1.549006	0.6134595	-2.525034	0.0115687
CPSF4L	1.1406586	-2.66465	1.1156901	-2.388343	0.0169246
CTAGE11P	0.4663812	-2.505018	1.1940993	-2.09783	0.0359201
CTAGE12P	1.4447379	2.734936	1.2180551	2.2453303	0.0247469
DLL3	1.5462708	2.2883665	0.9992401	2.2901068	0.0220151
DMBX1	1.1638002	3.1261542	1.4676811	2.1299955	0.033172
DPYD-IT1	1.2898702	2.9472616	1.4839715	1.9860635	0.0470263
DUXA	0.5480643	-2.51815	1.0972913	-2.294878	0.0217401
FABP2	1.2144557	2.4886928	1.1429504	2.1774285	0.0294486
FABP7	1.2048728	2.6522005	1.2037487	2.2032843	0.0275747
FCRL4	1.6519758	2.8146438	1.1415099	2.4657201	0.0136738
FHP1	0.6991309	-1.563906	0.7688551	-2.034072	0.0419444
FSTL5	1.4934293	2.1901187	0.930898	2.3526945	0.0186379
FUT9	1.6436949	2.3889584	1.1982187	1.9937583	0.0461785
GABRB1	1.5951208	2.5637447	1.1507029	2.2279815	0.0258817
GDF2	0.8202768	-2.175348	0.9451902	-2.301492	0.0213639
GSTM5P1	0.914644	-2.712685	1.3459096	-2.015503	0.043852
HLA-DRB9	1.3694246	2.2727933	0.8479998	2.6801816	0.0073582
IFNA1	1.4299041	-3.166434	1.1203468	-2.826298	0.0047089
IFNA21	0.4956959	-2.133431	1.0738845	-1.986648	0.0469614
IGKV1-13	0.6727097	2.3249237	1.0481555	2.2181095	0.0265474
KRT85	1.4490822	-1.620111	0.7516338	-2.155452	0.0311265
LGI1	1.1675391	-1.515796	0.6704433	-2.260887	0.0237663
LIM2	0.917509	-1.733843	0.6613477	-2.621681	0.0087497
LINC00935	1.0917455	-2.283774	0.844095	-2.705589	0.0068183
LINC00948	0.8702674	-2.314135	1.0705204	-2.161692	0.0306419

LOR	1.0037872	2.5934977	0.921797	2.8135236	0.0049002
LRRC3C	1.4742054	2.1564029	0.9624092	2.2406301	0.02505
LRRC53	1.5716526	-1.872591	0.9146632	-2.047301	0.0406285
MED28P8	1.4654402	-3.22376	0.8116456	-3.971881	7.13E-05
MEIS1-AS1	1.4015381	-2.580826	1.0505954	-2.456537	0.0140284
MGC4294	1.6365351	2.3788554	0.8680761	2.7403764	0.0061369
MIR650	1.011729	2.1557146	1.0993508	1.9608978	0.0498909
MS4A18	1.5081705	2.6696475	1.0729244	2.4881971	0.0128393
MT2P1	0.5872035	-2.028946	1.0209263	-1.987358	0.0468827
MYH4	1.1912914	2.0655936	0.9113087	2.2666234	0.0234132
NANOGP7	1.60001	2.4512301	1.1734748	2.0888647	0.0367199
NPM1P24	1.5128265	2.4441552	1.0609253	2.3037958	0.0212341
NR2E1	1.1658893	3.1437733	1.5624906	2.012027	0.0442171
OACYLP	0.9431008	2.0901143	0.9994347	2.0912965	0.0365015
OR13G1	1.1288677	-3.169999	1.0934914	-2.89897	0.0037439
OR52B2	0.8048047	-2.316475	1.1514657	-2.011762	0.044245
OR6K4P	1.4093282	-1.999718	0.8260745	-2.420747	0.0154886
PLSCR2	1.6770324	3.3091891	1.064294	3.1092811	0.0018754
PPBPP2	0.6616003	-2.085015	1.0532289	-1.979641	0.0477439
PRDM12	1.1404138	2.2366628	0.8458442	2.6442964	0.0081861
PRL	1.524539	-2.431472	0.723422	-3.36107	0.0007764
PRSS44	1.0557131	2.3081965	1.0573286	2.1830456	0.0290325
RN7SL166P	1.3745216	3.0206481	1.3589502	2.2227806	0.0262306
RN7SL798P	1.0751189	2.7802085	1.1777952	2.3605194	0.0182494
RNU1-106P	0.8429337	2.0477781	1.0235523	2.0006581	0.0454292
RPS18P1	1.4877987	-1.968181	0.9528852	-2.065496	0.0388761
RPS20P1	1.432519	2.7160264	1.2870006	2.1103537	0.0348279
RPS7P6	1.093835	-2.45025	1.241723	-1.973266	0.0484653
SLC22A11	1.2049751	2.2926516	0.85516	2.6809621	0.0073411
SLC9A4	1.1879589	-2.404968	1.0145715	-2.370427	0.0177675
SNX18P14	1.5156994	2.7623116	1.3931614	1.9827649	0.0473937
SNX31	1.545201	2.0857021	1.0091024	2.0668883	0.0387447
SPRR1B	1.1404961	3.1453198	1.55304	2.0252665	0.04284
STMN4	0.5452464	-1.829747	0.8041901	-2.275267	0.0228899
SYNPR	1.32633	2.9648668	1.2763391	2.322946	0.0201821
TDPX2	1.6837429	-1.532247	0.5780986	-2.650494	0.0080374
TMPRSS11E	1.322179	1.6114647	0.8049608	2.0019169	0.0452937
TRAV1-1	1.5998055	2.012321	1.0098743	1.9926451	0.0463003
TRAV36DV7	1.6756179	2.3011109	0.7641546	3.011316	0.0026012
TRPM8	1.6108642	1.8518995	0.9222314	2.0080638	0.0446365
TUBB4AP1	1.1938295	1.9845514	1.0034587	1.9777112	0.0479613
UBE2V2P3	0.8468959	2.6493919	1.3255124	1.9987681	0.0456334
ZNF444P1	1.1655067	-1.887258	0.7780781	-2.425539	0.0152857

Supplementary Table 7 Immune cell abundance estimated by ImmunCellAI

Sample	Cell type	Value
P1N	CD4_naive	6.40E-02
P1T1	CD4_naive	4.60E-02
P1T2	CD4_naive	5.90E-02
P1T3	CD4_naive	5.80E-02
P2N	CD4_naive	1.00E-03
P2T1	CD4_naive	0
P2T2	CD4_naive	0
P2T3	CD4_naive	3.50E-02
P3N	CD4_naive	0
P3T1	CD4_naive	4.70E-02
P3T2	CD4_naive	0
P3T3	CD4_naive	0
P4N	CD4_naive	0
P4T1	CD4_naive	6.20E-02
P4T2	CD4_naive	4.30E-02
P4T3	CD4_naive	3.70E-02
P5N	CD4_naive	4.90E-02
P5T1	CD4_naive	0
P5T2	CD4_naive	3.30E-02
P6N	CD4_naive	0
P6T1	CD4_naive	0
P6T2	CD4_naive	0
P6T3	CD4_naive	0
P7N	CD4_naive	6.30E-02
P7T1	CD4_naive	4.20E-02
P7T2	CD4_naive	0
P8N	CD4_naive	0.01
P8T1	CD4_naive	2.50E-02
P8T2	CD4_naive	0
P8T3	CD4_naive	2.00E-03
P9N	CD4_naive	5.20E-02
P9T1	CD4_naive	3.40E-02
P9T2	CD4_naive	0
P9T3	CD4_naive	2.00E-03
P10N	CD4_naive	5.90E-02
P10T1	CD4_naive	0
P10T2	CD4_naive	6.90E-02
P1N	CD8_naive	0.13
P1T1	CD8_naive	0.128

P1T2	CD8_naive	0.123
P1T3	CD8_naive	7.00E-02
P2N	CD8_naive	6.90E-02
P2T1	CD8_naive	0.116
P2T2	CD8_naive	0.109
P2T3	CD8_naive	0.1
P3N	CD8_naive	0.111
P3T1	CD8_naive	3.40E-02
P3T2	CD8_naive	0.114
P3T3	CD8_naive	0.117
P4N	CD8_naive	0.138
P4T1	CD8_naive	0.06
P4T2	CD8_naive	0.109
P4T3	CD8_naive	0.132
P5N	CD8_naive	0.141
P5T1	CD8_naive	7.60E-02
P5T2	CD8_naive	0.1
P6N	CD8_naive	0.11
P6T1	CD8_naive	9.30E-02
P6T2	CD8_naive	0.118
P6T3	CD8_naive	0.158
P7N	CD8_naive	0.06
P7T1	CD8_naive	7.40E-02
P7T2	CD8_naive	0.147
P8N	CD8_naive	9.50E-02
P8T1	CD8_naive	2.20E-02
P8T2	CD8_naive	0.187
P8T3	CD8_naive	7.10E-02
P9N	CD8_naive	5.70E-02
P9T1	CD8_naive	7.30E-02
P9T2	CD8_naive	4.00E-03
P9T3	CD8_naive	0.137
P10N	CD8_naive	0
P10T1	CD8_naive	7.60E-02
P10T2	CD8_naive	7.70E-02
P1N	Cytotoxic	0.132
P1T1	Cytotoxic	0.259
P1T2	Cytotoxic	0.272
P1T3	Cytotoxic	0.263
P2N	Cytotoxic	0.278
P2T1	Cytotoxic	0.306
P2T2	Cytotoxic	0.285
P2T3	Cytotoxic	0.207
P3N	Cytotoxic	0.289

P3T1	Cytotoxic	8.50E-02
P3T2	Cytotoxic	0.213
P3T3	Cytotoxic	0.261
P4N	Cytotoxic	0.312
P4T1	Cytotoxic	0.131
P4T2	Cytotoxic	0.113
P4T3	Cytotoxic	0.18
P5N	Cytotoxic	0.283
P5T1	Cytotoxic	0.3
P5T2	Cytotoxic	0.274
P6N	Cytotoxic	0.309
P6T1	Cytotoxic	0.282
P6T2	Cytotoxic	0.3
P6T3	Cytotoxic	0.256
P7N	Cytotoxic	0.141
P7T1	Cytotoxic	7.40E-02
P7T2	Cytotoxic	0.266
P8N	Cytotoxic	0.239
P8T1	Cytotoxic	0.195
P8T2	Cytotoxic	0.287
P8T3	Cytotoxic	0.192
P9N	Cytotoxic	0.185
P9T1	Cytotoxic	6.70E-02
P9T2	Cytotoxic	6.30E-02
P9T3	Cytotoxic	0.286
P10N	Cytotoxic	0
P10T1	Cytotoxic	8.10E-02
P10T2	Cytotoxic	0
P1N	Exhausted	0
P1T1	Exhausted	7.70E-02
P1T2	Exhausted	7.60E-02
P1T3	Exhausted	0.115
P2N	Exhausted	3.80E-02
P2T1	Exhausted	0.133
P2T2	Exhausted	7.30E-02
P2T3	Exhausted	0.125
P3N	Exhausted	0.116
P3T1	Exhausted	0.167
P3T2	Exhausted	9.20E-02
P3T3	Exhausted	9.80E-02
P4N	Exhausted	2.50E-02
P4T1	Exhausted	0.16
P4T2	Exhausted	0.122
P4T3	Exhausted	0.164

P5N	Exhausted	7.00E-03
P5T1	Exhausted	0.165
P5T2	Exhausted	0.107
P6N	Exhausted	3.30E-02
P6T1	Exhausted	0.104
P6T2	Exhausted	0.102
P6T3	Exhausted	0.143
P7N	Exhausted	5.10E-02
P7T1	Exhausted	0.206
P7T2	Exhausted	0.186
P8N	Exhausted	2.30E-02
P8T1	Exhausted	0.114
P8T2	Exhausted	0.12
P8T3	Exhausted	0.144
P9N	Exhausted	8.80E-02
P9T1	Exhausted	0.205
P9T2	Exhausted	5.10E-02
P9T3	Exhausted	2.60E-02
P10N	Exhausted	0
P10T1	Exhausted	6.40E-02
P10T2	Exhausted	0.186
P1N	Tr1	0.207
P1T1	Tr1	0.375
P1T2	Tr1	0.302
P1T3	Tr1	0.372
P2N	Tr1	0.315
P2T1	Tr1	0.296
P2T2	Tr1	0.224
P2T3	Tr1	0.411
P3N	Tr1	0.177
P3T1	Tr1	0.369
P3T2	Tr1	0.108
P3T3	Tr1	0.186
P4N	Tr1	3.30E-02
P4T1	Tr1	0.28
P4T2	Tr1	0.232
P4T3	Tr1	0.428
P5N	Tr1	0.334
P5T1	Tr1	0.243
P5T2	Tr1	0.432
P6N	Tr1	7.10E-02
P6T1	Tr1	0.332
P6T2	Tr1	0.178
P6T3	Tr1	0.371

P7N	Tr1	0.39
P7T1	Tr1	0.426
P7T2	Tr1	0.363
P8N	Tr1	0.21
P8T1	Tr1	0.334
P8T2	Tr1	0.345
P8T3	Tr1	0.274
P9N	Tr1	0.365
P9T1	Tr1	0.351
P9T2	Tr1	0.137
P9T3	Tr1	0.127
P10N	Tr1	0
P10T1	Tr1	4.30E-02
P10T2	Tr1	0.3
P1N	nTreg	0.118
P1T1	nTreg	0.252
P1T2	nTreg	0.198
P1T3	nTreg	0.288
P2N	nTreg	0.239
P2T1	nTreg	0.291
P2T2	nTreg	0.245
P2T3	nTreg	0.236
P3N	nTreg	0.21
P3T1	nTreg	0.237
P3T2	nTreg	0.334
P3T3	nTreg	0.302
P4N	nTreg	0.192
P4T1	nTreg	0.215
P4T2	nTreg	0.194
P4T3	nTreg	0.238
P5N	nTreg	6.90E-02
P5T1	nTreg	0.241
P5T2	nTreg	0.153
P6N	nTreg	0.276
P6T1	nTreg	0.265
P6T2	nTreg	0.283
P6T3	nTreg	0.312
P7N	nTreg	0.128
P7T1	nTreg	0.218
P7T2	nTreg	0.215
P8N	nTreg	7.00E-02
P8T1	nTreg	0.23
P8T2	nTreg	0.414
P8T3	nTreg	0.344

P9N	nTreg	0.151
P9T1	nTreg	0.308
P9T2	nTreg	7.00E-02
P9T3	nTreg	0.208
P10N	nTreg	0
P10T1	nTreg	0.228
P10T2	nTreg	0.306
P1N	iTreg	0.118
P1T1	iTreg	0.259
P1T2	iTreg	0.192
P1T3	iTreg	0.255
P2N	iTreg	0.108
P2T1	iTreg	0.123
P2T2	iTreg	7.50E-02
P2T3	iTreg	0.24
P3N	iTreg	4.50E-02
P3T1	iTreg	0.238
P3T2	iTreg	4.40E-02
P3T3	iTreg	7.00E-02
P4N	iTreg	0
P4T1	iTreg	0.144
P4T2	iTreg	0.159
P4T3	iTreg	0.259
P5N	iTreg	0.182
P5T1	iTreg	6.50E-02
P5T2	iTreg	0.214
P6N	iTreg	2.10E-02
P6T1	iTreg	0.226
P6T2	iTreg	0.102
P6T3	iTreg	0.182
P7N	iTreg	0.2
P7T1	iTreg	0.212
P7T2	iTreg	0.112
P8N	iTreg	0.122
P8T1	iTreg	0.232
P8T2	iTreg	0.148
P8T3	iTreg	0.135
P9N	iTreg	0.198
P9T1	iTreg	0.276
P9T2	iTreg	8.80E-02
P9T3	iTreg	3.80E-02
P10N	iTreg	0
P10T1	iTreg	0
P10T2	iTreg	0.261

P1N	Th1	0.161
P1T1	Th1	0.181
P1T2	Th1	0.14
P1T3	Th1	0.158
P2N	Th1	0.21
P2T1	Th1	0.209
P2T2	Th1	0.172
P2T3	Th1	0.16
P3N	Th1	0.113
P3T1	Th1	0.178
P3T2	Th1	0.171
P3T3	Th1	0.178
P4N	Th1	8.90E-02
P4T1	Th1	0.144
P4T2	Th1	0.2
P4T3	Th1	0.142
P5N	Th1	0.145
P5T1	Th1	0.275
P5T2	Th1	0.186
P6N	Th1	0.15
P6T1	Th1	0.208
P6T2	Th1	0.196
P6T3	Th1	0.205
P7N	Th1	0.179
P7T1	Th1	0.198
P7T2	Th1	0.194
P8N	Th1	0
P8T1	Th1	9.60E-02
P8T2	Th1	0.252
P8T3	Th1	0.254
P9N	Th1	0.157
P9T1	Th1	0.141
P9T2	Th1	7.10E-02
P9T3	Th1	0.175
P10N	Th1	9.10E-02
P10T1	Th1	0.219
P10T2	Th1	0.188
P1N	Th2	0.494
P1T1	Th2	0.485
P1T2	Th2	0.395
P1T3	Th2	0.392
P2N	Th2	0.515
P2T1	Th2	0.496
P2T2	Th2	0.487

P2T3	Th2	0.391
P3N	Th2	0.468
P3T1	Th2	9.80E-02
P3T2	Th2	0.407
P3T3	Th2	0.412
P4N	Th2	0.434
P4T1	Th2	0
P4T2	Th2	0.239
P4T3	Th2	0.362
P5N	Th2	0.238
P5T1	Th2	0.389
P5T2	Th2	0.23
P6N	Th2	0.43
P6T1	Th2	0.492
P6T2	Th2	0.453
P6T3	Th2	0.488
P7N	Th2	7.70E-02
P7T1	Th2	9.70E-02
P7T2	Th2	0.354
P8N	Th2	0.169
P8T1	Th2	4.30E-02
P8T2	Th2	0.234
P8T3	Th2	0.422
P9N	Th2	0.168
P9T1	Th2	8.40E-02
P9T2	Th2	0.122
P9T3	Th2	0.377
P10N	Th2	0.193
P10T1	Th2	0.343
P10T2	Th2	4.20E-02
P1N	Th17	0
P1T1	Th17	0
P1T2	Th17	0
P1T3	Th17	0
P2N	Th17	0.04
P2T1	Th17	3.70E-02
P2T2	Th17	0.11
P2T3	Th17	5.40E-02
P3N	Th17	8.70E-02
P3T1	Th17	4.90E-02
P3T2	Th17	7.20E-02
P3T3	Th17	9.20E-02
P4N	Th17	7.90E-02
P4T1	Th17	0

P4T2	Th17	0.105
P4T3	Th17	8.90E-02
P5N	Th17	1.50E-02
P5T1	Th17	9.70E-02
P5T2	Th17	3.20E-02
P6N	Th17	8.80E-02
P6T1	Th17	0.118
P6T2	Th17	0.103
P6T3	Th17	0.126
P7N	Th17	4.80E-02
P7T1	Th17	5.90E-02
P7T2	Th17	7.80E-02
P8N	Th17	8.30E-02
P8T1	Th17	8.70E-02
P8T2	Th17	6.80E-02
P8T3	Th17	0.139
P9N	Th17	6.70E-02
P9T1	Th17	5.10E-02
P9T2	Th17	0.09
P9T3	Th17	7.90E-02
P10N	Th17	6.20E-02
P10T1	Th17	0
P10T2	Th17	0.122
P1N	Tfh	0.134
P1T1	Tfh	0.19
P1T2	Tfh	0.151
P1T3	Tfh	0.227
P2N	Tfh	0.275
P2T1	Tfh	0.283
P2T2	Tfh	0.206
P2T3	Tfh	0.216
P3N	Tfh	0.222
P3T1	Tfh	0.198
P3T2	Tfh	0.135
P3T3	Tfh	0.142
P4N	Tfh	0.233
P4T1	Tfh	0.186
P4T2	Tfh	0.163
P4T3	Tfh	0.233
P5N	Tfh	0.2
P5T1	Tfh	0.154
P5T2	Tfh	0.2
P6N	Tfh	0.238
P6T1	Tfh	0.198

P6T2	Tfh	0.288
P6T3	Tfh	0.217
P7N	Tfh	0.164
P7T1	Tfh	0.141
P7T2	Tfh	0.204
P8N	Tfh	9.80E-02
P8T1	Tfh	5.10E-02
P8T2	Tfh	7.00E-02
P8T3	Tfh	0.216
P9N	Tfh	0.165
P9T1	Tfh	0.158
P9T2	Tfh	0
P9T3	Tfh	0.207
P10N	Tfh	6.10E-02
P10T1	Tfh	0.173
P10T2	Tfh	0.143
P1N	Central_memory	0.132
P1T1	Central_memory	0.117
P1T2	Central_memory	0.127
P1T3	Central_memory	0.136
P2N	Central_memory	5.90E-02
P2T1	Central_memory	4.20E-02
P2T2	Central_memory	3.90E-02
P2T3	Central_memory	0.155
P3N	Central_memory	0
P3T1	Central_memory	7.30E-02
P3T2	Central_memory	2.40E-02
P3T3	Central_memory	3.80E-02
P4N	Central_memory	0
P4T1	Central_memory	5.20E-02
P4T2	Central_memory	0.12
P4T3	Central_memory	0.134
P5N	Central_memory	0.118
P5T1	Central_memory	1.10E-02
P5T2	Central_memory	8.90E-02
P6N	Central_memory	5.50E-02
P6T1	Central_memory	6.30E-02
P6T2	Central_memory	7.40E-02
P6T3	Central_memory	6.70E-02
P7N	Central_memory	0.107
P7T1	Central_memory	0.124
P7T2	Central_memory	0.101
P8N	Central_memory	8.40E-02
P8T1	Central_memory	5.20E-02

P8T2	Central_memory	5.60E-02
P8T3	Central_memory	6.40E-02
P9N	Central_memory	0.103
P9T1	Central_memory	4.20E-02
P9T2	Central_memory	0
P9T3	Central_memory	5.90E-02
P10N	Central_memory	0.106
P10T1	Central_memory	3.60E-02
P10T2	Central_memory	0.131
P1N	Effector_memory	4.90E-02
P1T1	Effector_memory	2.90E-02
P1T2	Effector_memory	1.40E-02
P1T3	Effector_memory	2.80E-02
P2N	Effector_memory	1.90E-02
P2T1	Effector_memory	7.00E-03
P2T2	Effector_memory	3.50E-02
P2T3	Effector_memory	4.00E-03
P3N	Effector_memory	2.50E-02
P3T1	Effector_memory	1.00E-03
P3T2	Effector_memory	3.10E-02
P3T3	Effector_memory	3.50E-02
P4N	Effector_memory	2.30E-02
P4T1	Effector_memory	0
P4T2	Effector_memory	6.50E-02
P4T3	Effector_memory	1.10E-02
P5N	Effector_memory	5.50E-02
P5T1	Effector_memory	5.20E-02
P5T2	Effector_memory	3.40E-02
P6N	Effector_memory	0.04
P6T1	Effector_memory	1.10E-02
P6T2	Effector_memory	2.50E-02
P6T3	Effector_memory	5.00E-03
P7N	Effector_memory	2.70E-02
P7T1	Effector_memory	2.60E-02
P7T2	Effector_memory	3.90E-02
P8N	Effector_memory	1.30E-02
P8T1	Effector_memory	1.30E-02
P8T2	Effector_memory	4.00E-03
P8T3	Effector_memory	4.30E-02
P9N	Effector_memory	2.30E-02
P9T1	Effector_memory	0
P9T2	Effector_memory	0
P9T3	Effector_memory	4.70E-02
P10N	Effector_memory	8.90E-02

P10T1	Effector_memory	5.90E-02
P10T2	Effector_memory	6.80E-02
P1N	NKT	0.1
P1T1	NKT	0.115
P1T2	NKT	7.90E-02
P1T3	NKT	0.131
P2N	NKT	0.248
P2T1	NKT	0.234
P2T2	NKT	0.208
P2T3	NKT	0.16
P3N	NKT	0.143
P3T1	NKT	0.171
P3T2	NKT	0.261
P3T3	NKT	0.273
P4N	NKT	0.175
P4T1	NKT	0.166
P4T2	NKT	0.119
P4T3	NKT	0.102
P5N	NKT	0.06
P5T1	NKT	0.177
P5T2	NKT	8.70E-02
P6N	NKT	0.104
P6T1	NKT	0.237
P6T2	NKT	0.206
P6T3	NKT	0.133
P7N	NKT	0.154
P7T1	NKT	0.114
P7T2	NKT	0.108
P8N	NKT	9.90E-02
P8T1	NKT	2.60E-02
P8T2	NKT	0.08
P8T3	NKT	0.126
P9N	NKT	0.185
P9T1	NKT	0.138
P9T2	NKT	7.20E-02
P9T3	NKT	0.234
P10N	NKT	0.02
P10T1	NKT	0
P10T2	NKT	2.50E-02
P1N	MAIT	0.328
P1T1	MAIT	0.349
P1T2	MAIT	0.312
P1T3	MAIT	0.369
P2N	MAIT	0.287

P2T1	MAIT	0.256
P2T2	MAIT	0.259
P2T3	MAIT	0.295
P3N	MAIT	0.184
P3T1	MAIT	0.326
P3T2	MAIT	0.246
P3T3	MAIT	0.299
P4N	MAIT	0.209
P4T1	MAIT	0.285
P4T2	MAIT	0.336
P4T3	MAIT	0.289
P5N	MAIT	0.264
P5T1	MAIT	0.172
P5T2	MAIT	0.231
P6N	MAIT	0.233
P6T1	MAIT	0.283
P6T2	MAIT	0.297
P6T3	MAIT	0.225
P7N	MAIT	0.246
P7T1	MAIT	0.29
P7T2	MAIT	0.28
P8N	MAIT	0.16
P8T1	MAIT	0.116
P8T2	MAIT	0.298
P8T3	MAIT	0.234
P9N	MAIT	0.242
P9T1	MAIT	0.24
P9T2	MAIT	0
P9T3	MAIT	0.304
P10N	MAIT	0.158
P10T1	MAIT	9.70E-02
P10T2	MAIT	7.20E-02
P1N	DC	0.09
P1T1	DC	0.09
P1T2	DC	0.105
P1T3	DC	0.144
P2N	DC	0.177
P2T1	DC	0.21
P2T2	DC	0.208
P2T3	DC	0.149
P3N	DC	0.217
P3T1	DC	0.145
P3T2	DC	0.217
P3T3	DC	0.204

P4N	DC	0.243
P4T1	DC	0.171
P4T2	DC	0.124
P4T3	DC	0.122
P5N	DC	1.80E-02
P5T1	DC	9.30E-02
P5T2	DC	8.20E-02
P6N	DC	0.214
P6T1	DC	0.157
P6T2	DC	0.191
P6T3	DC	0.198
P7N	DC	0.158
P7T1	DC	0.127
P7T2	DC	0.169
P8N	DC	0.172
P8T1	DC	0.149
P8T2	DC	0.136
P8T3	DC	0.146
P9N	DC	0.136
P9T1	DC	0.107
P9T2	DC	0.197
P9T3	DC	0.214
P10N	DC	0
P10T1	DC	0.114
P10T2	DC	0.118
P1N	Bcell	5.50E-02
P1T1	Bcell	6.20E-02
P1T2	Bcell	6.40E-02
P1T3	Bcell	0.109
P2N	Bcell	5.90E-02
P2T1	Bcell	2.90E-02
P2T2	Bcell	4.40E-02
P2T3	Bcell	5.80E-02
P3N	Bcell	2.50E-02
P3T1	Bcell	3.40E-02
P3T2	Bcell	2.40E-02
P3T3	Bcell	1.10E-02
P4N	Bcell	1.90E-02
P4T1	Bcell	0
P4T2	Bcell	4.30E-02
P4T3	Bcell	3.40E-02
P5N	Bcell	7.60E-02
P5T1	Bcell	2.10E-02
P5T2	Bcell	5.80E-02

P6N	Bcell	4.70E-02
P6T1	Bcell	8.40E-02
P6T2	Bcell	0.04
P6T3	Bcell	4.70E-02
P7N	Bcell	2.80E-02
P7T1	Bcell	8.90E-02
P7T2	Bcell	2.40E-02
P8N	Bcell	7.30E-02
P8T1	Bcell	9.70E-02
P8T2	Bcell	5.30E-02
P8T3	Bcell	6.40E-02
P9N	Bcell	0.113
P9T1	Bcell	0.158
P9T2	Bcell	0.104
P9T3	Bcell	1.50E-02
P10N	Bcell	7.70E-02
P10T1	Bcell	0.17
P10T2	Bcell	0.1
P1N	Monocyte	0.17
P1T1	Monocyte	0.156
P1T2	Monocyte	0.177
P1T3	Monocyte	0.122
P2N	Monocyte	0.135
P2T1	Monocyte	0.12
P2T2	Monocyte	0.124
P2T3	Monocyte	9.40E-02
P3N	Monocyte	0.151
P3T1	Monocyte	0
P3T2	Monocyte	0.117
P3T3	Monocyte	0.113
P4N	Monocyte	0.143
P4T1	Monocyte	0.06
P4T2	Monocyte	6.60E-02
P4T3	Monocyte	0.112
P5N	Monocyte	0.198
P5T1	Monocyte	0.142
P5T2	Monocyte	0.118
P6N	Monocyte	0.174
P6T1	Monocyte	0.161
P6T2	Monocyte	0.141
P6T3	Monocyte	0.127
P7N	Monocyte	2.80E-02
P7T1	Monocyte	7.90E-02
P7T2	Monocyte	0.128

P8N	Monocyte	0.119
P8T1	Monocyte	0.126
P8T2	Monocyte	6.90E-02
P8T3	Monocyte	0.11
P9N	Monocyte	0.148
P9T1	Monocyte	4.70E-02
P9T2	Monocyte	3.40E-02
P9T3	Monocyte	0.133
P10N	Monocyte	8.20E-02
P10T1	Monocyte	0.133
P10T2	Monocyte	1.60E-02
P1N	Macrophage	6.30E-02
P1T1	Macrophage	5.30E-02
P1T2	Macrophage	2.50E-02
P1T3	Macrophage	0
P2N	Macrophage	0.116
P2T1	Macrophage	0.139
P2T2	Macrophage	0.148
P2T3	Macrophage	0.105
P3N	Macrophage	0.114
P3T1	Macrophage	0.05
P3T2	Macrophage	0.205
P3T3	Macrophage	0.185
P4N	Macrophage	0.149
P4T1	Macrophage	0.118
P4T2	Macrophage	6.60E-02
P4T3	Macrophage	8.60E-02
P5N	Macrophage	0.131
P5T1	Macrophage	0.204
P5T2	Macrophage	0.123
P6N	Macrophage	0.11
P6T1	Macrophage	7.30E-02
P6T2	Macrophage	7.80E-02
P6T3	Macrophage	0.13
P7N	Macrophage	7.40E-02
P7T1	Macrophage	9.10E-02
P7T2	Macrophage	0.121
P8N	Macrophage	0.113
P8T1	Macrophage	9.40E-02
P8T2	Macrophage	0.229
P8T3	Macrophage	0.146
P9N	Macrophage	0.03
P9T1	Macrophage	8.80E-02
P9T2	Macrophage	0.149

P9T3	Macrophage	0.119
P10N	Macrophage	0.156
P10T1	Macrophage	0.157
P10T2	Macrophage	0.179
P1N	NK	0.12
P1T1	NK	8.60E-02
P1T2	NK	0.104
P1T3	NK	8.30E-02
P2N	NK	0.134
P2T1	NK	0.147
P2T2	NK	0.146
P2T3	NK	9.70E-02
P3N	NK	0.167
P3T1	NK	3.10E-02
P3T2	NK	0.125
P3T3	NK	0.13
P4N	NK	0.179
P4T1	NK	6.10E-02
P4T2	NK	4.80E-02
P4T3	NK	7.90E-02
P5N	NK	9.90E-02
P5T1	NK	8.30E-02
P5T2	NK	7.20E-02
P6N	NK	0.165
P6T1	NK	0.106
P6T2	NK	0.151
P6T3	NK	0.128
P7N	NK	7.70E-02
P7T1	NK	4.50E-02
P7T2	NK	0.158
P8N	NK	9.30E-02
P8T1	NK	7.60E-02
P8T2	NK	0.1
P8T3	NK	7.60E-02
P9N	NK	8.50E-02
P9T1	NK	0
P9T2	NK	6.30E-02
P9T3	NK	0.173
P10N	NK	3.50E-02
P10T1	NK	9.60E-02
P10T2	NK	5.10E-02
P1N	Neutrophil	0.106
P1T1	Neutrophil	0.115
P1T2	Neutrophil	8.80E-02

P1T3	Neutrophil	0.112
P2N	Neutrophil	0.252
P2T1	Neutrophil	0.168
P2T2	Neutrophil	0.199
P2T3	Neutrophil	0.137
P3N	Neutrophil	0.109
P3T1	Neutrophil	8.10E-02
P3T2	Neutrophil	0.155
P3T3	Neutrophil	0.14
P4N	Neutrophil	0.144
P4T1	Neutrophil	0.02
P4T2	Neutrophil	1.10E-02
P4T3	Neutrophil	2.10E-02
P5N	Neutrophil	0.133
P5T1	Neutrophil	0.112
P5T2	Neutrophil	0.135
P6N	Neutrophil	0.122
P6T1	Neutrophil	0.181
P6T2	Neutrophil	0.164
P6T3	Neutrophil	5.90E-02
P7N	Neutrophil	0.14
P7T1	Neutrophil	4.10E-02
P7T2	Neutrophil	0.03
P8N	Neutrophil	0.159
P8T1	Neutrophil	0.188
P8T2	Neutrophil	0
P8T3	Neutrophil	6.90E-02
P9N	Neutrophil	0.155
P9T1	Neutrophil	1.70E-02
P9T2	Neutrophil	0.132
P9T3	Neutrophil	0.116
P10N	Neutrophil	1.90E-02
P10T1	Neutrophil	3.10E-02
P10T2	Neutrophil	4.60E-02
P1N	Gamma_delta	0.113
P1T1	Gamma_delta	0.126
P1T2	Gamma_delta	0.175
P1T3	Gamma_delta	0.154
P2N	Gamma_delta	0.192
P2T1	Gamma_delta	0.181
P2T2	Gamma_delta	0.175
P2T3	Gamma_delta	0.12
P3N	Gamma_delta	0.19
P3T1	Gamma_delta	6.40E-02

P3T2	Gamma_delta	0.13
P3T3	Gamma_delta	0.166
P4N	Gamma_delta	0.204
P4T1	Gamma_delta	9.20E-02
P4T2	Gamma_delta	9.30E-02
P4T3	Gamma_delta	0.08
P5N	Gamma_delta	0.198
P5T1	Gamma_delta	0.205
P5T2	Gamma_delta	0.224
P6N	Gamma_delta	0.218
P6T1	Gamma_delta	0.143
P6T2	Gamma_delta	0.172
P6T3	Gamma_delta	0.135
P7N	Gamma_delta	0.194
P7T1	Gamma_delta	6.70E-02
P7T2	Gamma_delta	0.145
P8N	Gamma_delta	0.174
P8T1	Gamma_delta	0.144
P8T2	Gamma_delta	0.137
P8T3	Gamma_delta	5.90E-02
P9N	Gamma_delta	0.163
P9T1	Gamma_delta	2.40E-02
P9T2	Gamma_delta	0.117
P9T3	Gamma_delta	0.164
P10N	Gamma_delta	5.70E-02
P10T1	Gamma_delta	9.30E-02
P10T2	Gamma_delta	0
P1N	CD4_T	0.172
P1T1	CD4_T	0.199
P1T2	CD4_T	0.209
P1T3	CD4_T	0.259
P2N	CD4_T	0.222
P2T1	CD4_T	0.222
P2T2	CD4_T	0.197
P2T3	CD4_T	0.241
P3N	CD4_T	9.10E-02
P3T1	CD4_T	0.203
P3T2	CD4_T	7.70E-02
P3T3	CD4_T	0.132
P4N	CD4_T	0.08
P4T1	CD4_T	0.192
P4T2	CD4_T	0.183
P4T3	CD4_T	0.277
P5N	CD4_T	0.153

P5T1	CD4_T	8.90E-02
P5T2	CD4_T	0.205
P6N	CD4_T	8.90E-02
P6T1	CD4_T	0.199
P6T2	CD4_T	0.135
P6T3	CD4_T	0.204
P7N	CD4_T	0.147
P7T1	CD4_T	0.253
P7T2	CD4_T	0.268
P8N	CD4_T	8.40E-02
P8T1	CD4_T	0.133
P8T2	CD4_T	0.202
P8T3	CD4_T	0.176
P9N	CD4_T	0.185
P9T1	CD4_T	0.201
P9T2	CD4_T	7.30E-02
P9T3	CD4_T	0.121
P10N	CD4_T	0
P10T1	CD4_T	3.60E-02
P10T2	CD4_T	0.119
P1N	CD8_T	8.40E-02
P1T1	CD8_T	0.136
P1T2	CD8_T	0.143
P1T3	CD8_T	0.122
P2N	CD8_T	2.80E-02
P2T1	CD8_T	0.06
P2T2	CD8_T	1.70E-02
P2T3	CD8_T	5.30E-02
P3N	CD8_T	9.50E-02
P3T1	CD8_T	0.04
P3T2	CD8_T	6.60E-02
P3T3	CD8_T	8.70E-02
P4N	CD8_T	8.10E-02
P4T1	CD8_T	2.90E-02
P4T2	CD8_T	7.40E-02
P4T3	CD8_T	7.10E-02
P5N	CD8_T	7.60E-02
P5T1	CD8_T	7.30E-02
P5T2	CD8_T	3.80E-02
P6N	CD8_T	0.08
P6T1	CD8_T	0.1
P6T2	CD8_T	0.126
P6T3	CD8_T	6.70E-02
P7N	CD8_T	8.10E-02

P7T1	CD8_T	9.00E-03
P7T2	CD8_T	6.90E-02
P8N	CD8_T	0.101
P8T1	CD8_T	6.90E-02
P8T2	CD8_T	5.10E-02
P8T3	CD8_T	8.10E-02
P9N	CD8_T	9.60E-02
P9T1	CD8_T	4.90E-02
P9T2	CD8_T	6.00E-03
P9T3	CD8_T	0.08
P10N	CD8_T	0
P10T1	CD8_T	6.50E-02
P10T2	CD8_T	4.20E-02

Supplementary Table 8 Immune cell abundance estimated by Cibersort

Sample	Cell type	Value
P1N	B.cells.naive	3.82E-02
P1T1	B.cells.naive	2.02E-02
P1T2	B.cells.naive	2.49E-02
P1T3	B.cells.naive	3.62E-02
P2N	B.cells.naive	3.59E-04
P2T1	B.cells.naive	0
P2T2	B.cells.naive	0
P2T3	B.cells.naive	2.80E-02
P3N	B.cells.naive	0
P3T1	B.cells.naive	3.13E-02
P3T2	B.cells.naive	0
P3T3	B.cells.naive	0
P4N	B.cells.naive	0
P4T1	B.cells.naive	5.39E-02
P4T2	B.cells.naive	5.47E-02
P4T3	B.cells.naive	6.15E-02
P5N	B.cells.naive	1.05E-02
P5T1	B.cells.naive	0
P5T2	B.cells.naive	1.18E-02
P6N	B.cells.naive	0
P6T1	B.cells.naive	3.96E-03
P6T2	B.cells.naive	0
P6T3	B.cells.naive	3.85E-03
P7N	B.cells.naive	0.1076578
P7T1	B.cells.naive	0.1055318
P7T2	B.cells.naive	0
P8N	B.cells.naive	0
P8T1	B.cells.naive	4.11E-03
P8T2	B.cells.naive	0
P8T3	B.cells.naive	4.91E-02
P9N	B.cells.naive	5.16E-02
P9T1	B.cells.naive	0.1129797
P9T2	B.cells.naive	7.64E-02
P9T3	B.cells.naive	0
P10N	B.cells.naive	2.25E-02
P10T1	B.cells.naive	8.66E-03
P10T2	B.cells.naive	5.12E-02
P1N	B.cells.memory	0
P1T1	B.cells.memory	0

P1T2	B.cells.memory	0
P1T3	B.cells.memory	0
P2N	B.cells.memory	0
P2T1	B.cells.memory	1.92E-02
P2T2	B.cells.memory	0
P2T3	B.cells.memory	0
P3N	B.cells.memory	2.21E-02
P3T1	B.cells.memory	0
P3T2	B.cells.memory	1.80E-02
P3T3	B.cells.memory	2.06E-02
P4N	B.cells.memory	1.14E-02
P4T1	B.cells.memory	0
P4T2	B.cells.memory	0
P4T3	B.cells.memory	0
P5N	B.cells.memory	0
P5T1	B.cells.memory	2.20E-02
P5T2	B.cells.memory	0
P6N	B.cells.memory	0
P6T1	B.cells.memory	1.40E-02
P6T2	B.cells.memory	6.59E-03
P6T3	B.cells.memory	3.73E-02
P7N	B.cells.memory	0
P7T1	B.cells.memory	0
P7T2	B.cells.memory	1.62E-02
P8N	B.cells.memory	0
P8T1	B.cells.memory	3.49E-03
P8T2	B.cells.memory	4.59E-02
P8T3	B.cells.memory	7.45E-04
P9N	B.cells.memory	0
P9T1	B.cells.memory	0.1655664
P9T2	B.cells.memory	0
P9T3	B.cells.memory	5.95E-03
P10N	B.cells.memory	0
P10T1	B.cells.memory	9.50E-02
P10T2	B.cells.memory	6.25E-02
P1N	Plasma.cells	0
P1T1	Plasma.cells	1.67E-03
P1T2	Plasma.cells	0
P1T3	Plasma.cells	0
P2N	Plasma.cells	4.65E-03
P2T1	Plasma.cells	0
P2T2	Plasma.cells	0
P2T3	Plasma.cells	0
P3N	Plasma.cells	0

P3T1	Plasma.cells	0
P3T2	Plasma.cells	0
P3T3	Plasma.cells	1.47E-03
P4N	Plasma.cells	0
P4T1	Plasma.cells	1.03E-03
P4T2	Plasma.cells	0
P4T3	Plasma.cells	4.05E-04
P5N	Plasma.cells	0
P5T1	Plasma.cells	0
P5T2	Plasma.cells	0
P6N	Plasma.cells	0
P6T1	Plasma.cells	0
P6T2	Plasma.cells	4.05E-03
P6T3	Plasma.cells	4.68E-05
P7N	Plasma.cells	8.73E-04
P7T1	Plasma.cells	0
P7T2	Plasma.cells	5.15E-03
P8N	Plasma.cells	0
P8T1	Plasma.cells	0
P8T2	Plasma.cells	0
P8T3	Plasma.cells	2.00E-03
P9N	Plasma.cells	1.21E-03
P9T1	Plasma.cells	0
P9T2	Plasma.cells	1.18E-02
P9T3	Plasma.cells	6.28E-03
P10N	Plasma.cells	0
P10T1	Plasma.cells	0
P10T2	Plasma.cells	0
P1N	T.cells.CD8	5.79E-02
P1T1	T.cells.CD8	7.79E-02
P1T2	T.cells.CD8	8.60E-02
P1T3	T.cells.CD8	7.32E-02
P2N	T.cells.CD8	7.62E-02
P2T1	T.cells.CD8	0.1334661
P2T2	T.cells.CD8	6.51E-02
P2T3	T.cells.CD8	4.56E-02
P3N	T.cells.CD8	0.1292752
P3T1	T.cells.CD8	0.1464026
P3T2	T.cells.CD8	5.40E-02
P3T3	T.cells.CD8	6.09E-02
P4N	T.cells.CD8	0.1090997
P4T1	T.cells.CD8	0.1556779
P4T2	T.cells.CD8	7.13E-02
P4T3	T.cells.CD8	5.22E-02

P5N	T.cells.CD8	0.1457474
P5T1	T.cells.CD8	0.1130839
P5T2	T.cells.CD8	6.88E-02
P6N	T.cells.CD8	0.1330335
P6T1	T.cells.CD8	6.97E-02
P6T2	T.cells.CD8	0.1426765
P6T3	T.cells.CD8	4.50E-02
P7N	T.cells.CD8	0.185962
P7T1	T.cells.CD8	0.1305782
P7T2	T.cells.CD8	0.1092206
P8N	T.cells.CD8	0.1110271
P8T1	T.cells.CD8	0.1020287
P8T2	T.cells.CD8	1.60E-02
P8T3	T.cells.CD8	8.27E-02
P9N	T.cells.CD8	4.34E-02
P9T1	T.cells.CD8	0.1209117
P9T2	T.cells.CD8	0.1921513
P9T3	T.cells.CD8	7.98E-02
P10N	T.cells.CD8	8.21E-02
P10T1	T.cells.CD8	3.67E-02
P10T2	T.cells.CD8	4.38E-02
P1N	T.cells.CD4.naive	0
P1T1	T.cells.CD4.naive	0
P1T2	T.cells.CD4.naive	0
P1T3	T.cells.CD4.naive	0
P2N	T.cells.CD4.naive	0
P2T1	T.cells.CD4.naive	0
P2T2	T.cells.CD4.naive	0
P2T3	T.cells.CD4.naive	0
P3N	T.cells.CD4.naive	0
P3T1	T.cells.CD4.naive	0
P3T2	T.cells.CD4.naive	0
P3T3	T.cells.CD4.naive	0
P4N	T.cells.CD4.naive	0
P4T1	T.cells.CD4.naive	0
P4T2	T.cells.CD4.naive	1.45E-02
P4T3	T.cells.CD4.naive	0
P5N	T.cells.CD4.naive	0
P5T1	T.cells.CD4.naive	0
P5T2	T.cells.CD4.naive	0
P6N	T.cells.CD4.naive	0
P6T1	T.cells.CD4.naive	0
P6T2	T.cells.CD4.naive	0
P6T3	T.cells.CD4.naive	0

P7N	T.cells.CD4.naive	0
P7T1	T.cells.CD4.naive	0
P7T2	T.cells.CD4.naive	0
P8N	T.cells.CD4.naive	0
P8T1	T.cells.CD4.naive	0
P8T2	T.cells.CD4.naive	0
P8T3	T.cells.CD4.naive	0
P9N	T.cells.CD4.naive	0
P9T1	T.cells.CD4.naive	0
P9T2	T.cells.CD4.naive	0
P9T3	T.cells.CD4.naive	0
P10N	T.cells.CD4.naive	0
P10T1	T.cells.CD4.naive	0
P10T2	T.cells.CD4.naive	0
P1N	T.cells.CD4.memory.resting	0.2078578
P1T1	T.cells.CD4.memory.resting	0.2572786
P1T2	T.cells.CD4.memory.resting	0.2035769
P1T3	T.cells.CD4.memory.resting	0.3428495
P2N	T.cells.CD4.memory.resting	0.2029973
P2T1	T.cells.CD4.memory.resting	0.1120581
P2T2	T.cells.CD4.memory.resting	0.1451423
P2T3	T.cells.CD4.memory.resting	0.2693118
P3N	T.cells.CD4.memory.resting	0.1109961
P3T1	T.cells.CD4.memory.resting	0.2492519
P3T2	T.cells.CD4.memory.resting	7.77E-02
P3T3	T.cells.CD4.memory.resting	6.45E-02
P4N	T.cells.CD4.memory.resting	5.68E-02
P4T1	T.cells.CD4.memory.resting	0
P4T2	T.cells.CD4.memory.resting	0.1554247
P4T3	T.cells.CD4.memory.resting	0.1448786
P5N	T.cells.CD4.memory.resting	0.1656064
P5T1	T.cells.CD4.memory.resting	5.54E-02
P5T2	T.cells.CD4.memory.resting	0.2512214
P6N	T.cells.CD4.memory.resting	0.1533495
P6T1	T.cells.CD4.memory.resting	0.1884488
P6T2	T.cells.CD4.memory.resting	0.1949377
P6T3	T.cells.CD4.memory.resting	9.72E-02
P7N	T.cells.CD4.memory.resting	0.3329663
P7T1	T.cells.CD4.memory.resting	0.1880891
P7T2	T.cells.CD4.memory.resting	7.73E-02
P8N	T.cells.CD4.memory.resting	0.1900759
P8T1	T.cells.CD4.memory.resting	8.56E-02
P8T2	T.cells.CD4.memory.resting	4.21E-03
P8T3	T.cells.CD4.memory.resting	3.95E-02

P9N	T.cells.CD4.memory.resting	0.328039
P9T1	T.cells.CD4.memory.resting	2.96E-02
P9T2	T.cells.CD4.memory.resting	6.56E-02
P9T3	T.cells.CD4.memory.resting	5.76E-02
P10N	T.cells.CD4.memory.resting	0.2299151
P10T1	T.cells.CD4.memory.resting	9.32E-02
P10T2	T.cells.CD4.memory.resting	0.1869537
P1N	T.cells.CD4.memory.activated	0
P1T1	T.cells.CD4.memory.activated	0
P1T2	T.cells.CD4.memory.activated	0
P1T3	T.cells.CD4.memory.activated	0
P2N	T.cells.CD4.memory.activated	0
P2T1	T.cells.CD4.memory.activated	0
P2T2	T.cells.CD4.memory.activated	0
P2T3	T.cells.CD4.memory.activated	0
P3N	T.cells.CD4.memory.activated	0
P3T1	T.cells.CD4.memory.activated	0
P3T2	T.cells.CD4.memory.activated	0
P3T3	T.cells.CD4.memory.activated	0
P4N	T.cells.CD4.memory.activated	0
P4T1	T.cells.CD4.memory.activated	0
P4T2	T.cells.CD4.memory.activated	0
P4T3	T.cells.CD4.memory.activated	0
P5N	T.cells.CD4.memory.activated	0
P5T1	T.cells.CD4.memory.activated	0
P5T2	T.cells.CD4.memory.activated	0
P6N	T.cells.CD4.memory.activated	0
P6T1	T.cells.CD4.memory.activated	0
P6T2	T.cells.CD4.memory.activated	0
P6T3	T.cells.CD4.memory.activated	0
P7N	T.cells.CD4.memory.activated	0
P7T1	T.cells.CD4.memory.activated	0
P7T2	T.cells.CD4.memory.activated	0
P8N	T.cells.CD4.memory.activated	0
P8T1	T.cells.CD4.memory.activated	0
P8T2	T.cells.CD4.memory.activated	0
P8T3	T.cells.CD4.memory.activated	0
P9N	T.cells.CD4.memory.activated	0
P9T1	T.cells.CD4.memory.activated	0
P9T2	T.cells.CD4.memory.activated	0
P9T3	T.cells.CD4.memory.activated	0
P10N	T.cells.CD4.memory.activated	0
P10T1	T.cells.CD4.memory.activated	0
P10T2	T.cells.CD4.memory.activated	0

P1N	T.cells.follicular.helper	9.03E-03
P1T1	T.cells.follicular.helper	7.32E-03
P1T2	T.cells.follicular.helper	0
P1T3	T.cells.follicular.helper	0
P2N	T.cells.follicular.helper	0
P2T1	T.cells.follicular.helper	1.24E-02
P2T2	T.cells.follicular.helper	6.63E-03
P2T3	T.cells.follicular.helper	0
P3N	T.cells.follicular.helper	5.65E-03
P3T1	T.cells.follicular.helper	2.39E-02
P3T2	T.cells.follicular.helper	3.66E-02
P3T3	T.cells.follicular.helper	4.66E-02
P4N	T.cells.follicular.helper	0
P4T1	T.cells.follicular.helper	6.07E-02
P4T2	T.cells.follicular.helper	0
P4T3	T.cells.follicular.helper	3.88E-02
P5N	T.cells.follicular.helper	0
P5T1	T.cells.follicular.helper	4.16E-02
P5T2	T.cells.follicular.helper	1.02E-02
P6N	T.cells.follicular.helper	0
P6T1	T.cells.follicular.helper	1.38E-02
P6T2	T.cells.follicular.helper	0
P6T3	T.cells.follicular.helper	6.11E-02
P7N	T.cells.follicular.helper	0
P7T1	T.cells.follicular.helper	5.03E-02
P7T2	T.cells.follicular.helper	3.86E-02
P8N	T.cells.follicular.helper	1.26E-02
P8T1	T.cells.follicular.helper	4.79E-02
P8T2	T.cells.follicular.helper	2.14E-02
P8T3	T.cells.follicular.helper	7.11E-02
P9N	T.cells.follicular.helper	0
P9T1	T.cells.follicular.helper	0.1087075
P9T2	T.cells.follicular.helper	4.46E-02
P9T3	T.cells.follicular.helper	1.41E-02
P10N	T.cells.follicular.helper	0
P10T1	T.cells.follicular.helper	5.28E-02
P10T2	T.cells.follicular.helper	2.68E-02
P1N	T.cells.regulatory..Tregs.	0
P1T1	T.cells.regulatory..Tregs.	0
P1T2	T.cells.regulatory..Tregs.	0
P1T3	T.cells.regulatory..Tregs.	0
P2N	T.cells.regulatory..Tregs.	0
P2T1	T.cells.regulatory..Tregs.	7.83E-03
P2T2	T.cells.regulatory..Tregs.	0

P2T3	T.cells.regulatory..Tregs.	0
P3N	T.cells.regulatory..Tregs.	0
P3T1	T.cells.regulatory..Tregs.	0
P3T2	T.cells.regulatory..Tregs.	1.85E-02
P3T3	T.cells.regulatory..Tregs.	2.21E-02
P4N	T.cells.regulatory..Tregs.	0
P4T1	T.cells.regulatory..Tregs.	4.77E-03
P4T2	T.cells.regulatory..Tregs.	0
P4T3	T.cells.regulatory..Tregs.	0
P5N	T.cells.regulatory..Tregs.	0
P5T1	T.cells.regulatory..Tregs.	6.98E-03
P5T2	T.cells.regulatory..Tregs.	0
P6N	T.cells.regulatory..Tregs.	0
P6T1	T.cells.regulatory..Tregs.	0
P6T2	T.cells.regulatory..Tregs.	0
P6T3	T.cells.regulatory..Tregs.	1.44E-02
P7N	T.cells.regulatory..Tregs.	0
P7T1	T.cells.regulatory..Tregs.	0
P7T2	T.cells.regulatory..Tregs.	0
P8N	T.cells.regulatory..Tregs.	0
P8T1	T.cells.regulatory..Tregs.	6.75E-03
P8T2	T.cells.regulatory..Tregs.	1.74E-02
P8T3	T.cells.regulatory..Tregs.	2.29E-02
P9N	T.cells.regulatory..Tregs.	0
P9T1	T.cells.regulatory..Tregs.	0
P9T2	T.cells.regulatory..Tregs.	0
P9T3	T.cells.regulatory..Tregs.	2.38E-02
P10N	T.cells.regulatory..Tregs.	0
P10T1	T.cells.regulatory..Tregs.	0
P10T2	T.cells.regulatory..Tregs.	2.55E-02
P1N	T.cells.gamma.delta	0
P1T1	T.cells.gamma.delta	0
P1T2	T.cells.gamma.delta	0
P1T3	T.cells.gamma.delta	0
P2N	T.cells.gamma.delta	0
P2T1	T.cells.gamma.delta	0
P2T2	T.cells.gamma.delta	0
P2T3	T.cells.gamma.delta	0
P3N	T.cells.gamma.delta	0
P3T1	T.cells.gamma.delta	0
P3T2	T.cells.gamma.delta	0
P3T3	T.cells.gamma.delta	0
P4N	T.cells.gamma.delta	0
P4T1	T.cells.gamma.delta	0

P4T2	T.cells.gamma.delta	0
P4T3	T.cells.gamma.delta	0
P5N	T.cells.gamma.delta	0
P5T1	T.cells.gamma.delta	0
P5T2	T.cells.gamma.delta	0
P6N	T.cells.gamma.delta	0
P6T1	T.cells.gamma.delta	0
P6T2	T.cells.gamma.delta	0
P6T3	T.cells.gamma.delta	0
P7N	T.cells.gamma.delta	0
P7T1	T.cells.gamma.delta	0
P7T2	T.cells.gamma.delta	0
P8N	T.cells.gamma.delta	0
P8T1	T.cells.gamma.delta	0
P8T2	T.cells.gamma.delta	0
P8T3	T.cells.gamma.delta	0
P9N	T.cells.gamma.delta	0
P9T1	T.cells.gamma.delta	0
P9T2	T.cells.gamma.delta	0
P9T3	T.cells.gamma.delta	0
P10N	T.cells.gamma.delta	0
P10T1	T.cells.gamma.delta	0
P10T2	T.cells.gamma.delta	0
P1N	NK.cells.resting	0
P1T1	NK.cells.resting	0
P1T2	NK.cells.resting	7.95E-02
P1T3	NK.cells.resting	7.84E-02
P2N	NK.cells.resting	3.73E-02
P2T1	NK.cells.resting	0
P2T2	NK.cells.resting	1.94E-02
P2T3	NK.cells.resting	2.84E-02
P3N	NK.cells.resting	2.56E-02
P3T1	NK.cells.resting	0
P3T2	NK.cells.resting	0
P3T3	NK.cells.resting	0
P4N	NK.cells.resting	2.41E-02
P4T1	NK.cells.resting	1.82E-02
P4T2	NK.cells.resting	3.34E-02
P4T3	NK.cells.resting	0
P5N	NK.cells.resting	0.1323708
P5T1	NK.cells.resting	0
P5T2	NK.cells.resting	7.29E-02
P6N	NK.cells.resting	5.18E-02
P6T1	NK.cells.resting	0

P6T2	NK.cells.resting	1.61E-02
P6T3	NK.cells.resting	0
P7N	NK.cells.resting	8.33E-02
P7T1	NK.cells.resting	0
P7T2	NK.cells.resting	0
P8N	NK.cells.resting	9.11E-02
P8T1	NK.cells.resting	2.19E-02
P8T2	NK.cells.resting	0
P8T3	NK.cells.resting	0
P9N	NK.cells.resting	8.04E-02
P9T1	NK.cells.resting	0
P9T2	NK.cells.resting	1.66E-02
P9T3	NK.cells.resting	0
P10N	NK.cells.resting	4.04E-02
P10T1	NK.cells.resting	0
P10T2	NK.cells.resting	1.57E-03
P1N	NK.cells.activated	5.26E-02
P1T1	NK.cells.activated	2.81E-02
P1T2	NK.cells.activated	2.32E-02
P1T3	NK.cells.activated	5.89E-02
P2N	NK.cells.activated	4.80E-02
P2T1	NK.cells.activated	5.58E-02
P2T2	NK.cells.activated	4.30E-02
P2T3	NK.cells.activated	1.97E-02
P3N	NK.cells.activated	0.1196577
P3T1	NK.cells.activated	0.1120018
P3T2	NK.cells.activated	5.54E-02
P3T3	NK.cells.activated	5.52E-02
P4N	NK.cells.activated	5.51E-02
P4T1	NK.cells.activated	5.05E-02
P4T2	NK.cells.activated	0
P4T3	NK.cells.activated	2.52E-02
P5N	NK.cells.activated	6.02E-03
P5T1	NK.cells.activated	6.79E-02
P5T2	NK.cells.activated	1.57E-02
P6N	NK.cells.activated	9.77E-02
P6T1	NK.cells.activated	5.23E-02
P6T2	NK.cells.activated	6.30E-02
P6T3	NK.cells.activated	4.03E-02
P7N	NK.cells.activated	5.36E-02
P7T1	NK.cells.activated	2.49E-02
P7T2	NK.cells.activated	6.07E-02
P8N	NK.cells.activated	5.81E-03
P8T1	NK.cells.activated	0.1014876

P8T2	NK.cells.activated	6.61E-03
P8T3	NK.cells.activated	5.08E-02
P9N	NK.cells.activated	4.36E-02
P9T1	NK.cells.activated	6.60E-02
P9T2	NK.cells.activated	8.92E-02
P9T3	NK.cells.activated	7.14E-02
P10N	NK.cells.activated	9.72E-03
P10T1	NK.cells.activated	3.49E-02
P10T2	NK.cells.activated	2.95E-02
P1N	Monocytes	4.99E-02
P1T1	Monocytes	8.23E-02
P1T2	Monocytes	3.73E-02
P1T3	Monocytes	0.1099511
P2N	Monocytes	0.1485251
P2T1	Monocytes	5.20E-02
P2T2	Monocytes	8.31E-02
P2T3	Monocytes	0.1043832
P3N	Monocytes	9.17E-02
P3T1	Monocytes	0.118409
P3T2	Monocytes	9.96E-02
P3T3	Monocytes	4.53E-02
P4N	Monocytes	5.99E-02
P4T1	Monocytes	3.29E-02
P4T2	Monocytes	7.58E-03
P4T3	Monocytes	1.73E-02
P5N	Monocytes	4.95E-03
P5T1	Monocytes	6.65E-02
P5T2	Monocytes	2.72E-02
P6N	Monocytes	7.70E-02
P6T1	Monocytes	4.54E-02
P6T2	Monocytes	8.06E-02
P6T3	Monocytes	0.1354225
P7N	Monocytes	5.07E-02
P7T1	Monocytes	7.65E-02
P7T2	Monocytes	8.42E-03
P8N	Monocytes	4.77E-02
P8T1	Monocytes	9.93E-02
P8T2	Monocytes	0
P8T3	Monocytes	0.1514651
P9N	Monocytes	3.90E-02
P9T1	Monocytes	2.12E-02
P9T2	Monocytes	0.1069865
P9T3	Monocytes	5.27E-02
P10N	Monocytes	1.87E-02

P10T1	Monocytes	4.69E-03
P10T2	Monocytes	1.82E-02
P1N	Macrophages.M0	2.85E-02
P1T1	Macrophages.M0	8.64E-02
P1T2	Macrophages.M0	5.62E-02
P1T3	Macrophages.M0	5.65E-02
P2N	Macrophages.M0	0
P2T1	Macrophages.M0	0
P2T2	Macrophages.M0	7.13E-03
P2T3	Macrophages.M0	0.16075
P3N	Macrophages.M0	4.41E-03
P3T1	Macrophages.M0	0.1068956
P3T2	Macrophages.M0	1.79E-02
P3T3	Macrophages.M0	7.44E-02
P4N	Macrophages.M0	0.1617835
P4T1	Macrophages.M0	0.1987994
P4T2	Macrophages.M0	0.117941
P4T3	Macrophages.M0	0.1079597
P5N	Macrophages.M0	0.1041571
P5T1	Macrophages.M0	2.02E-02
P5T2	Macrophages.M0	0.100403
P6N	Macrophages.M0	1.37E-02
P6T1	Macrophages.M0	1.52E-02
P6T2	Macrophages.M0	0
P6T3	Macrophages.M0	8.31E-04
P7N	Macrophages.M0	8.06E-02
P7T1	Macrophages.M0	8.26E-02
P7T2	Macrophages.M0	2.17E-02
P8N	Macrophages.M0	0.2273551
P8T1	Macrophages.M0	0.1816788
P8T2	Macrophages.M0	0.4012691
P8T3	Macrophages.M0	0
P9N	Macrophages.M0	0.1111812
P9T1	Macrophages.M0	0.1047711
P9T2	Macrophages.M0	3.47E-02
P9T3	Macrophages.M0	8.55E-02
P10N	Macrophages.M0	0
P10T1	Macrophages.M0	0
P10T2	Macrophages.M0	0.2062848
P1N	Macrophages.M1	4.48E-03
P1T1	Macrophages.M1	1.95E-03
P1T2	Macrophages.M1	1.68E-03
P1T3	Macrophages.M1	1.26E-02
P2N	Macrophages.M1	3.75E-02

P2T1	Macrophages.M1	2.92E-02
P2T2	Macrophages.M1	3.91E-03
P2T3	Macrophages.M1	1.09E-02
P3N	Macrophages.M1	7.24E-03
P3T1	Macrophages.M1	0
P3T2	Macrophages.M1	0
P3T3	Macrophages.M1	1.56E-02
P4N	Macrophages.M1	0
P4T1	Macrophages.M1	9.92E-03
P4T2	Macrophages.M1	4.39E-03
P4T3	Macrophages.M1	5.61E-03
P5N	Macrophages.M1	0
P5T1	Macrophages.M1	6.45E-05
P5T2	Macrophages.M1	0
P6N	Macrophages.M1	1.42E-03
P6T1	Macrophages.M1	8.98E-04
P6T2	Macrophages.M1	1.51E-02
P6T3	Macrophages.M1	0
P7N	Macrophages.M1	5.09E-04
P7T1	Macrophages.M1	5.67E-03
P7T2	Macrophages.M1	5.64E-03
P8N	Macrophages.M1	0
P8T1	Macrophages.M1	6.25E-04
P8T2	Macrophages.M1	5.85E-03
P8T3	Macrophages.M1	0
P9N	Macrophages.M1	1.11E-02
P9T1	Macrophages.M1	0
P9T2	Macrophages.M1	0
P9T3	Macrophages.M1	4.69E-03
P10N	Macrophages.M1	7.03E-04
P10T1	Macrophages.M1	2.29E-02
P10T2	Macrophages.M1	3.21E-02
P1N	Macrophages.M2	0.3181475
P1T1	Macrophages.M2	0.2562537
P1T2	Macrophages.M2	0.3343036
P1T3	Macrophages.M2	0.1521745
P2N	Macrophages.M2	0.2857125
P2T1	Macrophages.M2	0.4433436
P2T2	Macrophages.M2	0.4316263
P2T3	Macrophages.M2	0.1846256
P3N	Macrophages.M2	0.3477643
P3T1	Macrophages.M2	0.1230066
P3T2	Macrophages.M2	0.4139846
P3T3	Macrophages.M2	0.4299487

P4N	Macrophages.M2	0.3002909
P4T1	Macrophages.M2	0.3588125
P4T2	Macrophages.M2	0.4557086
P4T3	Macrophages.M2	0.3742419
P5N	Macrophages.M2	0.295799
P5T1	Macrophages.M2	0.4038356
P5T2	Macrophages.M2	0.3031363
P6N	Macrophages.M2	0.3770331
P6T1	Macrophages.M2	0.4299659
P6T2	Macrophages.M2	0.3558587
P6T3	Macrophages.M2	0.4756655
P7N	Macrophages.M2	8.50E-02
P7T1	Macrophages.M2	0.2552713
P7T2	Macrophages.M2	0.5322683
P8N	Macrophages.M2	0.2350391
P8T1	Macrophages.M2	0.2008128
P8T2	Macrophages.M2	0.353775
P8T3	Macrophages.M2	0.3245354
P9N	Macrophages.M2	0.2555
P9T1	Macrophages.M2	0.2537567
P9T2	Macrophages.M2	0.2646919
P9T3	Macrophages.M2	0.3869134
P10N	Macrophages.M2	0.3699386
P10T1	Macrophages.M2	0.3894833
P10T2	Macrophages.M2	0.2794249
P1N	Dendritic.cells.resting	1.09E-04
P1T1	Dendritic.cells.resting	0
P1T2	Dendritic.cells.resting	0
P1T3	Dendritic.cells.resting	0
P2N	Dendritic.cells.resting	0
P2T1	Dendritic.cells.resting	4.44E-04
P2T2	Dendritic.cells.resting	0
P2T3	Dendritic.cells.resting	0
P3N	Dendritic.cells.resting	1.02E-03
P3T1	Dendritic.cells.resting	0
P3T2	Dendritic.cells.resting	1.55E-02
P3T3	Dendritic.cells.resting	0
P4N	Dendritic.cells.resting	0
P4T1	Dendritic.cells.resting	0
P4T2	Dendritic.cells.resting	0
P4T3	Dendritic.cells.resting	0
P5N	Dendritic.cells.resting	0
P5T1	Dendritic.cells.resting	3.92E-02
P5T2	Dendritic.cells.resting	0

P6N	Dendritic.cells.resting	0
P6T1	Dendritic.cells.resting	0
P6T2	Dendritic.cells.resting	1.89E-02
P6T3	Dendritic.cells.resting	7.08E-03
P7N	Dendritic.cells.resting	0
P7T1	Dendritic.cells.resting	0
P7T2	Dendritic.cells.resting	8.74E-03
P8N	Dendritic.cells.resting	0
P8T1	Dendritic.cells.resting	0
P8T2	Dendritic.cells.resting	0.1078447
P8T3	Dendritic.cells.resting	7.59E-02
P9N	Dendritic.cells.resting	0
P9T1	Dendritic.cells.resting	0
P9T2	Dendritic.cells.resting	0
P9T3	Dendritic.cells.resting	0
P10N	Dendritic.cells.resting	0
P10T1	Dendritic.cells.resting	0.1150763
P10T2	Dendritic.cells.resting	0
P1N	Dendritic.cells.activated	0
P1T1	Dendritic.cells.activated	1.16E-02
P1T2	Dendritic.cells.activated	0
P1T3	Dendritic.cells.activated	1.76E-02
P2N	Dendritic.cells.activated	6.52E-02
P2T1	Dendritic.cells.activated	8.15E-03
P2T2	Dendritic.cells.activated	4.46E-02
P2T3	Dendritic.cells.activated	6.91E-03
P3N	Dendritic.cells.activated	8.85E-03
P3T1	Dendritic.cells.activated	1.12E-02
P3T2	Dendritic.cells.activated	2.27E-02
P3T3	Dendritic.cells.activated	0
P4N	Dendritic.cells.activated	5.81E-02
P4T1	Dendritic.cells.activated	0
P4T2	Dendritic.cells.activated	3.02E-03
P4T3	Dendritic.cells.activated	7.60E-03
P5N	Dendritic.cells.activated	3.41E-02
P5T1	Dendritic.cells.activated	6.40E-04
P5T2	Dendritic.cells.activated	4.22E-02
P6N	Dendritic.cells.activated	4.29E-02
P6T1	Dendritic.cells.activated	2.72E-02
P6T2	Dendritic.cells.activated	2.46E-02
P6T3	Dendritic.cells.activated	1.45E-02
P7N	Dendritic.cells.activated	1.44E-02
P7T1	Dendritic.cells.activated	3.13E-02
P7T2	Dendritic.cells.activated	1.12E-02

P8N	Dendritic.cells.activated	3.47E-02
P8T1	Dendritic.cells.activated	3.36E-03
P8T2	Dendritic.cells.activated	0
P8T3	Dendritic.cells.activated	1.73E-02
P9N	Dendritic.cells.activated	0
P9T1	Dendritic.cells.activated	7.27E-03
P9T2	Dendritic.cells.activated	1.14E-02
P9T3	Dendritic.cells.activated	2.55E-02
P10N	Dendritic.cells.activated	5.58E-02
P10T1	Dendritic.cells.activated	2.26E-02
P10T2	Dendritic.cells.activated	3.86E-03
P1N	Mast.cells.resting	0.2331965
P1T1	Mast.cells.resting	0.1690734
P1T2	Mast.cells.resting	0.1532778
P1T3	Mast.cells.resting	6.17E-02
P2N	Mast.cells.resting	8.11E-02
P2T1	Mast.cells.resting	0.1112497
P2T2	Mast.cells.resting	0.1394282
P2T3	Mast.cells.resting	0.1414509
P3N	Mast.cells.resting	0.1250132
P3T1	Mast.cells.resting	7.75E-02
P3T2	Mast.cells.resting	0.1699845
P3T3	Mast.cells.resting	0.1598122
P4N	Mast.cells.resting	0.1519794
P4T1	Mast.cells.resting	5.48E-02
P4T2	Mast.cells.resting	8.20E-02
P4T3	Mast.cells.resting	0.1643655
P5N	Mast.cells.resting	8.19E-02
P5T1	Mast.cells.resting	7.33E-02
P5T2	Mast.cells.resting	0
P6N	Mast.cells.resting	1.31E-02
P6T1	Mast.cells.resting	0.105979
P6T2	Mast.cells.resting	5.21E-02
P6T3	Mast.cells.resting	5.13E-02
P7N	Mast.cells.resting	4.37E-03
P7T1	Mast.cells.resting	4.92E-02
P7T2	Mast.cells.resting	0.1049109
P8N	Mast.cells.resting	3.42E-02
P8T1	Mast.cells.resting	0.1265289
P8T2	Mast.cells.resting	1.14E-02
P8T3	Mast.cells.resting	0.1121003
P9N	Mast.cells.resting	2.23E-02
P9T1	Mast.cells.resting	9.25E-03
P9T2	Mast.cells.resting	8.11E-02

P9T3	Mast.cells.resting	0.1763932
P10N	Mast.cells.resting	0.1702253
P10T1	Mast.cells.resting	0.1200879
P10T2	Mast.cells.resting	3.25E-02
P1N	Mast.cells.activated	0
P1T1	Mast.cells.activated	0
P1T2	Mast.cells.activated	0
P1T3	Mast.cells.activated	0
P2N	Mast.cells.activated	0
P2T1	Mast.cells.activated	1.49E-02
P2T2	Mast.cells.activated	0
P2T3	Mast.cells.activated	0
P3N	Mast.cells.activated	0
P3T1	Mast.cells.activated	0
P3T2	Mast.cells.activated	0
P3T3	Mast.cells.activated	0
P4N	Mast.cells.activated	0
P4T1	Mast.cells.activated	0
P4T2	Mast.cells.activated	0
P4T3	Mast.cells.activated	0
P5N	Mast.cells.activated	0
P5T1	Mast.cells.activated	8.59E-02
P5T2	Mast.cells.activated	9.64E-02
P6N	Mast.cells.activated	1.94E-02
P6T1	Mast.cells.activated	0
P6T2	Mast.cells.activated	0
P6T3	Mast.cells.activated	1.58E-02
P7N	Mast.cells.activated	0
P7T1	Mast.cells.activated	0
P7T2	Mast.cells.activated	0
P8N	Mast.cells.activated	0
P8T1	Mast.cells.activated	0
P8T2	Mast.cells.activated	8.11E-03
P8T3	Mast.cells.activated	0
P9N	Mast.cells.activated	0
P9T1	Mast.cells.activated	0
P9T2	Mast.cells.activated	0
P9T3	Mast.cells.activated	0
P10N	Mast.cells.activated	0
P10T1	Mast.cells.activated	3.85E-03
P10T2	Mast.cells.activated	0
P1N	Eosinophils	0
P1T1	Eosinophils	0
P1T2	Eosinophils	0

P1T3	Eosinophils	0
P2N	Eosinophils	0
P2T1	Eosinophils	0
P2T2	Eosinophils	0
P2T3	Eosinophils	0
P3N	Eosinophils	0
P3T1	Eosinophils	0
P3T2	Eosinophils	0
P3T3	Eosinophils	0
P4N	Eosinophils	0
P4T1	Eosinophils	0
P4T2	Eosinophils	0
P4T3	Eosinophils	0
P5N	Eosinophils	0
P5T1	Eosinophils	0
P5T2	Eosinophils	4.91E-06
P6N	Eosinophils	0
P6T1	Eosinophils	0
P6T2	Eosinophils	0
P6T3	Eosinophils	0
P7N	Eosinophils	0
P7T1	Eosinophils	0
P7T2	Eosinophils	0
P8N	Eosinophils	0
P8T1	Eosinophils	0
P8T2	Eosinophils	0
P8T3	Eosinophils	0
P9N	Eosinophils	0
P9T1	Eosinophils	0
P9T2	Eosinophils	0
P9T3	Eosinophils	0
P10N	Eosinophils	0
P10T1	Eosinophils	0
P10T2	Eosinophils	0
P1N	Neutrophils	0
P1T1	Neutrophils	0
P1T2	Neutrophils	0
P1T3	Neutrophils	0
P2N	Neutrophils	1.25E-02
P2T1	Neutrophils	0
P2T2	Neutrophils	1.10E-02
P2T3	Neutrophils	0
P3N	Neutrophils	7.07E-04
P3T1	Neutrophils	0

P3T2	Neutrophils	0
P3T3	Neutrophils	3.68E-03
P4N	Neutrophils	1.14E-02
P4T1	Neutrophils	0
P4T2	Neutrophils	0
P4T3	Neutrophils	0
P5N	Neutrophils	1.89E-02
P5T1	Neutrophils	3.40E-03
P5T2	Neutrophils	0
P6N	Neutrophils	1.97E-02
P6T1	Neutrophils	3.32E-02
P6T2	Neutrophils	2.55E-02
P6T3	Neutrophils	1.70E-04
P7N	Neutrophils	0
P7T1	Neutrophils	0
P7T2	Neutrophils	0
P8N	Neutrophils	1.04E-02
P8T1	Neutrophils	1.44E-02
P8T2	Neutrophils	3.10E-04
P8T3	Neutrophils	0
P9N	Neutrophils	1.27E-02
P9T1	Neutrophils	0
P9T2	Neutrophils	4.76E-03
P9T3	Neutrophils	9.43E-03
P10N	Neutrophils	0
P10T1	Neutrophils	0
P10T2	Neutrophils	0
